# Supplementary material for: Mn-catalysed acceptorless dehydrogenative condensation of ureas with 1,2-diols for synthesizing imidazolones
Source: Commun Chem. 2025 Nov 6;8:333. doi: 10.1038/s42004-025-01710-z (PMC12592549; doi:10.1038/s42004-025-01710-z)
Supplement: Supplementary file 2 — Supplemental Information [file 42004_2025_1710_MOESM2_ESM.pdf]

Supplementary Information

Mn-Catalysed Acceptorless Dehydrogenative Condensation of Ureas with 1,2-Diols for Synthesizing Imidazolones

Ding et al.

Supplementary methods

General Information: The synthesis of ligands and preparation of catalysts **[Mn]-I**, **[Mn]-II** and **[Mn]-IV** in this work were reported in our previous publication[1]. Air and moisture sensitive reactions were carried out in glovebox or in over-dried glassware sealed with rubber septa using standard schlenk techniques. Most solvents used were dried over solvent purification system (Innovative Technology PS-MD-5) and alcohol solvents were dried over calcium hydride. Deuterated solvents were purchased from Cambridge Isotope Laboratories, vented and distilled over calcium hydride. All chemicals were purchased from commercial sources with purity over 95% and used without further purification. NMR spectra were received using a Bruker 400 MHz spectrometer. Chemical shifts are reported in ppm relative to the deuterated solvent. GC analysis were carried out on SHIMADAZU GC 2010 PLUS system. (Column: SH-Rtx-200, 30 m x 0.25 mm x 0.25 μm). GC/MS analyses were carried out on an GC-MS-QP2010 SE W system equipped with aSH-Rxi-5Sil MS 30 meter, 0.25 mmID, 0.25 um df. High resolution exact mass measurements (HRMS) were performed on Thermo SCIENTIFIC Q EXACTIVE.

Supplementary Table S1. Optimization of Reaction Conditions.

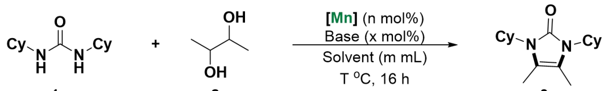

1a 0.5 mmol; 2a 2 equiv.; 3a

Entry	[Mn]	Base	Solvent	n [mol]	m [mL]	x [mol%]	T [°C]	Y_{3a} [%]
1	[Mn]-I	^{t}BuOK	dioxane	1	0.5	10	160	14
2	[Mn]-II	^{t}BuOK	dioxane	1	0.5	10	160	9
3	[Mn]-III	^{t}BuOK	dioxane	1	0.5	10	160	62
4	[Mn]-IV	^{t}BuOK	dioxane	1	0.5	10	160	6
5	none	^{t}BuOK	dioxane	1	0.5	10	160	trace
6	$MnCl_2$	^{t}BuOK	dioxane	1	0.5	10	160	trace
7	$Mn(CO)_5Br$	^{t}BuOK	dioxane	1	0.5	10	160	trace
8	[Mn]-III	^{t}BuONa	dioxane	1	0.5	10	160	79
9	[Mn]-III	NaOH	dioxane	1	0.5	10	160	83
10	[Mn]-III	KOH	dioxane	1	0.5	10	160	67
11	[Mn]-III	EtONa	dioxane	1	0.5	10	160	76
12	[Mn]-III	Na_2CO_3	dioxane	1	0.5	10	160	76
13	[Mn]-III	Cs_2CO_3	dioxane	1	0.5	10	160	42
14	[Mn]-III	Na_2CO_3	toluene	1	0.5	10	160	86
15	[Mn]-III	Na_2CO_3	THF	1	0.5	10	160	50
16	[Mn]-III	Na_2CO_3	toluene	1	0.5	5	160	86
17	[Mn]-III	Na_2CO_3	toluene	1	0.5	15	160	86
18	[Mn]-III	Na_2CO_3	toluene	1	0.2	5	160	93
19	[Mn]-III	Na_2CO_3	toluene	1	0.1	5	160	76
20	[Mn]-III	Na_2CO_3	toluene	1	1	5	160	64
21	[Mn]-III	Na_2CO_3	toluene	0.5	0.2	5	160	76
22	[Mn]-III	Na_2CO_3	toluene	2	0.2	5	160	93
23	[Mn]-III	Na_2CO_3	toluene	1	0.2	5	140	50
24[b]	[Mn]-III	Na_2CO_3	toluene	1	0.2	5	160	70

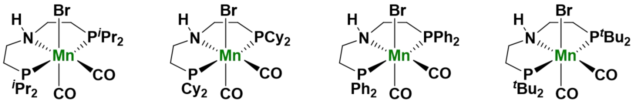

[Mn]-I [Mn]-II [Mn]-III [Mn]-IV

Reaction conditions: Unless otherwise specified, reactions were performed on a 0.5 mmol scale of *N*,*N*'-dicyclohexylurea **1a**, 1 mmol 2,3-butanediol **2a**, using 10 mol% of base, 1 mol% of Mn-precatalyst, in 0.5 mL dioxane at 160 °C for 16 h. [a] The yields were determined by GC using biphenyl as the internal standard. [b] 0.75 mmol 2,3-butanediol **2a** was used.

Supplementary Table S2. Homogenity test for the synthesis of **3a**

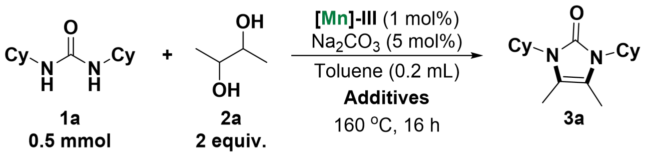

Entry	Additives	Quantity (equiv.)	Yield$_{3a}$ [%]
1	--	--	93
2	PMe_3	0.1	90
3	PMe_3	0.5	90
4	PMe_3	1	88
5	PPh_3	0.1	89
6	PPh_3	0.5	87
7	PPh_3	1	87
8	Hg	A drop	85

Reaction conditions: Unless otherwise specified, reactions were performed on a 0.5 mmol scale of *N*,*N*'-dicyclohexylurea **1a**, 1 mmol 2,3-butanediol **2a**, using 5 mol% of base, 1 mol% of **[Mn]-III**, in 0.2 mL dioxane with corresponding additives (equiv. respect to **[Mn]-III**) at 160 °C for 16 h. The yields were determined by GC using biphenyl as the internal standard.

Supplementary Figure 1. Synthesis of **[Mn]-III**

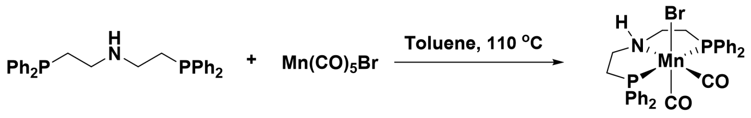

L-III **[Mn]-III**

Supplementary Figure 2. Synthesis of ligand **L-V**

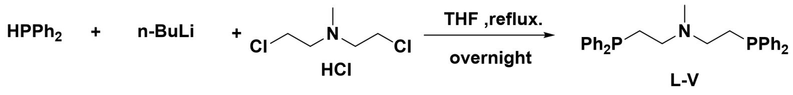

Supplementary Figure 3. Synthesis of **[Mn]-V**

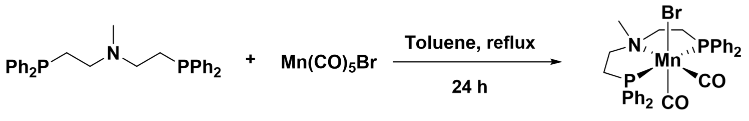

L-V **[Mn]-V**

Synthesis of Bromodicarbonyl(bis(2-(diphenylphosphaneyl)ethyl)amine)manganese [Mn]-III

In a 50 mL Schlenk flask, a solution of [$MnBr(CO)_5$] (274 mg, 1 mmol) in 10 mL of toluene was added to a solution of bis(2-(diphenylphosphaneyl)ethyl)amine **L-III** (463 mg, 1.05 mmol) in 5 mL of toluene at room temperature. The resulting solution was then heated to 110 °C for 20 hours. After that, it was cooled to room temperature and concentrated in vacuo. Hexane (20 mL) was added to the reaction Schlenk flask and then the suspension was filtered. The crude precipitate was extracted with dichloromethane and the resulted filtrate solution was concentrated under reduced pressure and dried to afford the complex **[Mn]-III** as a yellow powder (391 mg, 62%).

^{1}H NMR (400 MHz, DCM-d_2) δ 7.90 (m, J = 5.9 Hz, 4 H), 7.59 (m, 4 H), 7.52 – 7.22 (m, 12 H), 3.67 (m, 2 H), 3.54 (br, 1 H), 3.27 (m, 2 H), 2.77 (m, 2 H), 2.41 (m, 2 H). ^{13}C NMR (100 MHz, DCM-d_2) δ 138.03 (t, J = 18.9 Hz), 135.46 (t, J = 18.8 Hz), 133.88 (t, J = 4.8 Hz), 130.68 (t, J = 5.0 Hz), 130.32, 129.66, 129.06 (t, J = 4.2 Hz), 128.75 (t, J = 4.5 Hz), 53.05 (t, J = 4.8 Hz), 28.50 (t, J = 8.8 Hz). ^{31}P NMR (162 MHz, DCM-d_6) δ 69.65 (s).

Synthesis of 2-(diphenylphosphaneyl)-N-(2-(diphenylphosphaneyl)ethyl)-N-methylethan-1-amine L-V

A 50 mL Schlenk flask was charged with diphenylphosphane (1.50 g, 8 mmol) and degassed dry THF (10 mL) and cooled to –78 °C. To this cooled solution was added nBuLi (4 mL, 2.5 M in hexane, 10 mmol) dropwise and then the resulting solution was reacted for 1 h. After that the solution was cooled to –78 °C again. Another 50 mL Schlenk flask was charged with $MeN(CH_2CH_2Cl)_2$ ·HCl (0.62 g, 3.2 mmol) and THF (10 mL). It is then cooled to –78 °C. To the suspension was added nBuLi (1.5 mL, 2.5 M in hexane, 3.75 mmol) dropwise and stirred for 0.5 h while warming to room temperature. The resulting mixture was cooled to –78 °C and added dropwise to the flask containing the lithium phosphide solution at -78 ºC. The mixture was then stirred at reflux overnight. The solution was subsequently cooled to room temperature before removing the solvent under vacuum. The reaction mixture was quenched with 5 mL of water and extracted with Et_2O (3 ×5mL). The combined organic layer was dried with Na_2SO_4, filtered and concentrated under vacuum. After chromatography on silica-gel column (CH_2Cl_2/MeOH=100/1 to 50/1), the corresponding ligand was obtained as yellowish liqiud (0.71 g, 49%).

^{1}H NMR (400 MHz, $CDCl_3$) δ 7.39 (td, J = 7.2, 3.1 Hz, 8 H), 7.33 – 7.29 (m, 12 H), 2.48 (m, 4 H), 2.24 (s, 3 H), 2.36 – 2.12 (m, 4 H). ^{13}C NMR (100 MHz, $CDCl_3$) δ 138.44 (d, *J* = 12.2 Hz), 132.70 (d, *J* = 19.1 Hz), 128.58(s), 128.45 (s),128.38 (s), 53.38 (d, *J* = 27.3Hz), 41.75 (s) , 25.75 (d, *J* = 12.1Hz). ^{31}P NMR (162 MHz, $CDCl_3$) δ -19.79 (s).

HRMS (ESI) calcd. for $C_{29}H_{31}NP_2$ $[M+H]^+$: 456.2004; found: 456.1988.

Synthesis of {MnBr(CO)$_2$[Me-N(CH$_2$CH$_2$P(Ph)$_2$)$_2$]} [Mn]-V

2-(diphenylphosphaneyl)-*N*-(2-(diphenylphosphaneyl)ethyl)-*N*-methylethan-1-amine (160 mg, 0.35 mmol, 1.06 equivalents) was added to pentacarbonylbromomanganese (I) (92 mg, 0.33 mmol, 1.0 equivalents) in a 50 mL Schlenk flask at ambient temperature. Degassed toluene (10 mL) was added and the mixture was heated to reflux and kept at that temperature for 24 h. After that, it was cooled to room temperature and concentrated in vacuo. The crude material was dissolved in methylene chloride, filtered to remove insoluble material and the product precipitated by addition of n-pentane, collected by filtration and washed with n-hexane and to give the desired product as a yellow powder (175 mg, 0.27 mmol, 82% yield).

^{1}H NMR (400 MHz, DCM-*d$_2$*) δ 7.89 – 7.76 (m, 8 H), 7.49 – 7.35 (m, 12 H), 3.79 – 3.72 (m, 2 H), 3.24 – 3.14 (m, 2 H), 3.01 – 2.93 (m, 2 H), 2.81 – 2.71 (m, 2 H), 2.67 (s, 3 H). ^{13}C NMR (101 MHz, DCM-*d$_2$*) δ 132.34 (t, *J* = 5.0 Hz), 131.50 (t, *J* = 5.1 Hz), 129.50 (s), 129.03 (s), 128.53 (t, *J* = 4.4 Hz), 127.78 (t, *J* = 3.9 Hz), 59.08 (t, *J* = 4.4 Hz), 49.94 (s) , 27.77 (t, *J* = 8.8 Hz). ^{31}P NMR (162 MHz, DCM-*d$_6$*) δ 69.06 (s).

HRMS (ESI) calcd. for $C_{31}H_{31}BrMnNO_2P_2$ [M-Br]$^+$: 566.1205; found: 566.1205.

General Procedure A: Experimental Procedures for the Optimization of Reaction Conditions

All dehydrogenation experiments were carried out in a 15 mL pressure seal tube. In the argon atmosphere glovebox, *N*,*N*'-dicyclohexylurea **1a** (0.5 mmol), 2,3-butanediol **2a** (0.75-1.0 mmol), manganese catalysts (0.5-2.0 mol%), base (5-15 mol%), Solvent (0.1-1.0 mL) were added sequentially to the seal tube equipped with a magnetic stir bar, The reaction mixture was stirred at given temperature for 16 hours and cooled to room temperature. After the gas was released, the yield of product **3a** was determined by GC with biphenyl as the internal standard.

General Procedure B: Experimental Procedures for Mn-catalyzed Dehydrogenative Condensation of *N*,*N*'-dicyclohexylurea with 1,2-diols.

All experiments were carried out in a 15 mL pressure seal tube. In the argon atmosphere glovebox, *N*,*N*'-dicyclohexylurea **1a** (0.5 mmol), 1,2-diols **2** (1.0 mmol), **[Mn]-III** (0.5 mol%), Na_2CO_3 (5 mol%) were added sequentially to the seal tube equipped with a magnetic stir bar. The reaction mixture was stirred at 160 ºC for 16 hours and cooled to room temperature. After the gas was released, the resulting solution was concentrated in vacuum and the residue was purified by chromatography on silica gel, eluting with the mixture of ethyl acetate/petroleum ether to give the corresponding imidazolone products.

General Procedure C: Experimental Procedures for Mn-catalyzed Dehydrogenative Condensation of *N*,*N*'-disubstituted ureas with 1,2-diols.

All experiments were carried out in a 15 mL pressure seal tube. In the argon atmosphere glovebox, *N*,*N*'-disubstituted ureas **1** (0.5 mmol), 1,2-diols **2a** (1.0 mmol), **[Mn]-III** (0.5 mol%), Na_2CO_3 (5 mol%) were added sequentially to the seal tube equipped with a magnetic stir bar. The reaction mixture was stirred at 160 ^{o}C for 16 hours and cooled to room temperature. After the gas was released, the resulting solution was concentrated in vacuum and the residue was purified by chromatography on silica gel, eluting with the mixture of ethyl acetate/petroleum ether to give the corresponding imidazolone products.

General Procedure D: Experimental Procedures of Homogenity Test with Phosphine or Mercury Additives

All dehydrogenation experiments were carried out in a 15 mL pressure seal tube. In the argon atmosphere glovebox, *N*,*N*'-dicyclohexylurea **1a** (0.5 mmol), 2,3-butanediol **2a** (1.0 mmol), **[Mn]-III** (0.5 mol%), Na_2CO_3 (5 mol%) and phosphines or mercury (equiv. respect to **[Mn]-III**) were added sequentially to the seal tube equipped with a magnetic stir bar, The reaction mixture was stirred at 165 ºC for 16 hours and cooled to room temperature. After the gas was released, the yield of product **3a** was determined by GC with biphenyl as the internal standard.

General Procedure E: Experimental Procedures for the Synthesis of *N,N'*-dibenzylureas

A 25 mL round bottom flask containing a stirring bar was sequentially charged with the corresponding benzylamine (4 mmol), dichloromethane (10 mL), DABCO (45 mg, 0.4 mmol, 0.1 eq.) and $(Boc)_2O$ (440 mg, 2 mmol, 0.5 eq.), and this mixture was stirred at room temperature for 12 hours. Then, the solvent was evaporated under a reduced pressure, and the crude of the reaction was purified by neutral-alumina or silica gel column chromatography (PE/AcOEt) to yield the desired cyclic product.

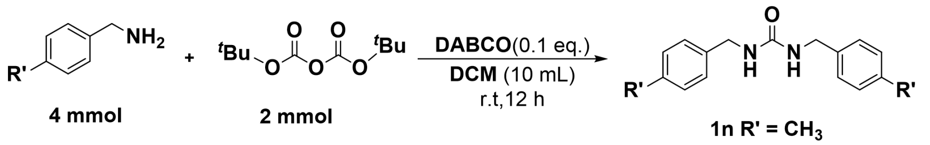

1n R' = CH_3
1o R' = OMe
1p R' = F
1q R' = Cl
1r R' = CF_3

General Procedure F: Experimental Procedures for the Synthesis of Diols via Alkene Oxidation

A 25 mL round bottom flask containing a stirring bar was sequentially charged with the corresponding the styrene derivative (3.0 mmol, 1.0 equiv.), I_2 (30 mg, 16 μmol, 4 mol%), distilled water (1.5 mL) and tert-butylhydroperoxide (70% solution in H_2O, 1.5 mL, 10.8 mmol, 3.6 equiv.) , and this mixture was stirred at 90 °C for 24 h. Then,the reaction was quenched with a 2 M $Na_2S_2O_3$ (4 mL) and the product was extracted with EtOAc (5 x 10 mL). The combined organic layers were dried over $MgSO_4$, and the solvent was removed under reduced pressure. The crude product was purified by flash column chromatography using PE/Acetone as eluent.

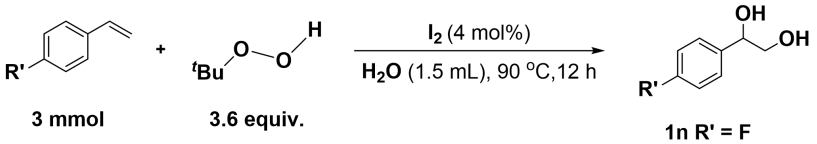

1n R' = F
1o R' = OMe

General Procedure G: Experimental Procedures for the Synthesis of 1,2-Diols

To a solution of 1-phenyl-2-propanedione (300 mg, 2.0 mmol, 1.0 equiv) in ethanol (5 mL) was added $NaBH_4$ (93 mg, 2.4 mmol, 1.2 equiv) slowly at 0 °C. The resulting mixture was stirred at 0 °C for 1 h, quenched with water (2 mL), and extracted with EtOAc (3 × 10 mL). The combined organic layers were dried over Na_2SO_4, filtered, and concentrated under reduced pressure to afford 1-phenylpropanediol as a white solid.

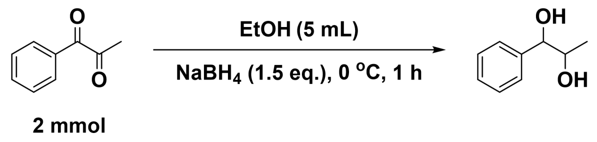

Characterization Data of Products

1,3-dicyclohexyl-4,5-dimethyl-1,3-dihydro-2H-imidazol-2-one **3a**[2]

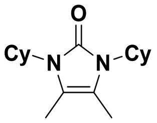

Product was isolated via column chromatography (PE/EA = 1:1) as white solid (128.3 mg, 0.47 mmol, 93 %). ^{1}H NMR (400 MHz, CD_3CN) δ 3.78 – 3.67 (m, 2H), 2.16 – 2.06 (m, 4H), 1.99 (s, 6H), 1.85 – 1.79 (m, 4H), 1.68 – 1.61 (m, 6H), 1.37 – 1.29 (m, 4H), 1.26 – 1.18 (m, 2H). ^{13}C NMR (101 MHz, CD_3CN) δ 152.89 (s), 113.32(s), 53.59(s), 31.12(s), 26.57(s), 25.76(s), 9.17(s).

1,3-dicyclohexyl-4-ethyl-5-methyl-1,3-dihydro-2H-imidazol-2-one **3b**

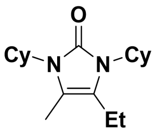

Product was isolated via column chromatography (PE/EA = 1:1) as white solid (98.6 mg, 0.34 mmol, 68 %). ^{1}H NMR (400 MHz, CD_3CN) δ 3.57 (ddt, J = 12.3, 8.4, 3.9 Hz, 1H), 3.37 (tt, J = 12.1, 3.9 Hz, 1H), 2.24 (q, J = 7.4 Hz, 2H), 1.84 (s, 3H), 1.70 – 1.60 (m, 5H), 1.52 – 1.43 (m, 6H), 1.25 – 0.98 (m, 8H), 0.86 (t, J = 7.5 Hz, 3H). ^{13}C NMR (101 MHz, CD_3CN) δ 152.87(s), 119.28(s), 112.85(s), 53.84(s), 53.49(s), 31.10(s), 30.53(s), 26.58(s), 26.51(s), 25.76(s), 25.73(s), 16.55(s), 14.93(s), 9.17(s). HRMS (ESI-TOF, m/z) calcd. for $C18H31N_2O$ $(M+H)^+$ 291.24309; found 291.24191.

1,3-dicyclohexyl-1,3-dihydro-2H-imidazol-2-one **3c**[2]

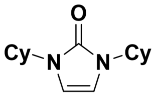

Product was isolated via column chromatography (PE/EA = 1:1) as colorless oil (106.6 mg, 0.43 mmol, 86 %). ^{1}H NMR (400 MHz, CD_3CN) δ 6.27 (s, 2H), 3.67 (s, 2H), 1.75 – 1.69 (m, 8H), 1.61 – 1.56 (m, 3H), 1.36 – 1.19 (m, 8H), 1.13 – 1.08 (m, 2H). ^{13}C NMR (101 MHz, CD_3CN) δ 107.75(s), 52.25(s), 32.84(s), 25.99(s), 25.67(s).

1,3-dicyclohexyl-4-methyl-1,3-dihydro-2H-imidazol-2-one **3d**[2]

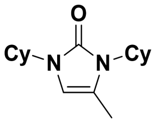

Product was isolated via column chromatography (PE/EA = 1:1) as colorless oil (98.3 mg, 0.38 mmol, 75 %). ^{1}H NMR (400 MHz, CD_3CN) δ 6.06 (d, J = 1.5 Hz, 1H), 3.85 – 3.72 (m, 2H), 2.06 (d, J = 1.4 Hz, 3H), 1.85 – 1.76 (m, 6H), 1.71 – 1.65 (m, 4H), 1.49 – 1.24 (m, 8H), 1.19 (ddt, J = 13.0, 9.5, 3.3 Hz, 2H). ^{13}C NMR (101 MHz, CD_3CN) δ 152.74(s), 118.70(s), 104.08(s), 53.74(s), 51.75(s), 32.81(s), 31.10(s), 26.51(s), 26.03(s), 25.73(s), 11.30(s).

1,3-dicyclohexyl-4-ethyl-1,3-dihydro-2H-imidazol-2-one **3e**[2]

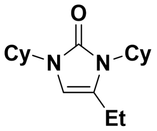

Product was isolated via column chromatography (PE/EA = 1:1) as white solid (122.8 mg, 0.45 mmol, 89 %). ^{1}H NMR (400 MHz, CD_3CN) δ 6.04 (t, J = 1.5 Hz, 1H), 3.83 (ddd, J = 11.5, 7.7, 3.7 Hz, 1H), 3.67 (ddd, J = 12.1, 8.3, 3.9 Hz, 1H), 2.46 – 2.39 (m, 2H), 1.87 – 1.77 (m, 6H), 1.77 – 1.63 (m, 6H), 1.47 – 1.33 (m, 6H), 1.26 – 1.18 (m, 2H), 1.14 (t, J = 7.4 Hz, 3H). ^{13}C NMR (101 MHz, CD_3CN) δ 124.83(s), 102.65(s), 53.83(s), 51.72(s), 32.80(s), 30.70(s), 26.48(s), 26.05(s), 25.73(s), 25.72(s), 18.81(s), 11.84(s).

1,3-dicyclohexyl-4-propyl-1,3-dihydro-2H-imidazol-2-one **3f**[2]

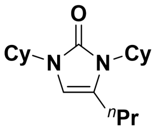

Product was isolated via column chromatography (PE/EA = 1:1) as white solid (111.7 mg, 0.39 mmol, 77 %). ^{1}H NMR (400 MHz, CD_3CN) δ 6.05 (d, J = 1.4 Hz, 1H), 3.82 (ddd, J = 11.6, 7.7, 3.8 Hz, 1H), 3.63 (tt, J = 12.1, 3.9 Hz, 1H), 2.39 – 2.35 (m, 2H), 1.85 – 1.74 (m, 6H), 1.72 – 1.45 (m, 8H), 1.44 – 1.28 (m, 6H), 1.26 – 1.15 (m, 2H), 0.98 (t, J = 7.4 Hz, 3H).. ^{13}C NMR (101 MHz, CD_3CN) δ 152.69(s), 123.00(s), 103.33(s), 53.88(s), 51.67(s), 32.78(s), 30.59(s), 27.47(s), 26.46(s), 26.04(s), 25.73(s), 25.70(s), 21.50(s), 13.64(s).

1,3-dicyclohexyl-4-isopropyl-1,3-dihydro-2H-imidazol-2-one **3g**

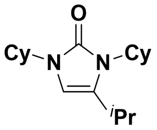

Product was isolated via column chromatography (PE/EA = 1:1) as white solid (143.6 mg, 0.50 mmol, 99 %). ^{1}H NMR (400 MHz, CD_3CN) δ 5.82 (d, J = 1.1 Hz, 1H), 3.63 – 3.56 (m, 1H), 3.38 – 3.30 (m, 1H), 2.18 – 2.09 (m, 2H), 1.63 – 1.37 (m, 12H), 1.24 – 1.09 (m, 6H), 1.06 – 0.97 (m, 2H), 0.92 (d, J = 6.8 Hz, 6H).^{13}C NMR (101 MHz, CD_3CN) δ 152.75(s), 129.42(s), 101.61(s), 53.94(s), 51.65(s), 32.75(s), 30.21(s), 26.43(s), 26.04(s), 25.73(s), 25.68(s), 24.64(s), 21.97(s). HRMS (ESI-TOF, m/z) calcd. for $C_{18}H_{31}N_2O$ $(M+H)^+$ 291.24309; found 291.24215.

1,3-dicyclohexyl-4-phenyl-1,3-dihydro-2H-imidazol-2-one **3h**[2]

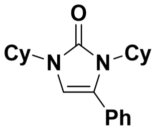

Product was isolated via column chromatography (PE/EA = 1:1) as white solid (105.3 mg, 0.33 mmol, 65 %). ^{1}H NMR (400 MHz, CD_3CN) δ 7.34 (dd, J = 8.2, 6.4 Hz, 2H), 7.30 – 7.27 (m, 1H), 7.24 – 7.20 (m, 2H), 6.28 (s, 1H), 3.80 (ddt, J = 11.6, 7.3, 3.5 Hz, 1H), 3.58 – 3.49 (m, 1H), 2.22 (dd, J = 12.2, 3.9 Hz, 2H), 1.77 – 1.71 (m, 4H), 1.70 – 1.65 (m, 2H), 1.59 – 1.56 (m, 2H), 1.52 – 1.44 (m, 2H), 1.43 – 1.31 (m, 5H), 1.11 – 1.04 (m, 4H). ^{13}C NMR (101 MHz, CD_3CN) δ 152.91(s), 131.15(s), 129.27(s), 129.02(s), 128.32(s), 124.64(s), 106.28(s), 54.89(s), 51.99(s), 32.79(s), 30.28(s), 26.34(s), 26.00(s), 25.69(s), 25.59(s).

1,3-dicyclohexyl-3,4,5,6-tetrahydrocyclopenta[d]imidazol-2(1H)-one **3i**[2]

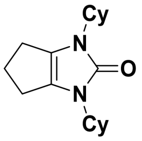

Product was isolated via column chromatography (PE/EA = 1:1) as white solid (82.1 mg, 0.29 mmol, 57 %). ^{1}H NMR (400 MHz, CD_3CN) δ 3.68 (ddd, J = 12.0, 8.3, 3.7 Hz, 2H), 2.43 (t, J = 6.9 Hz, 4H), 2.09 – 2.00 (m, 2H), 1.64 – 1.41 (m, 12H), 1.30 – 1.14 (m, 6H), 0.96 (dt, J = 12.7, 3.5 Hz, 2H). ^{13}C NMR (101 MHz, CD_3CN) δ 155.78(s), 122.65(s), 52.74(s), 32.96(s), 26.56(s), 26.19(s), 26.00(s),

25.74(s).

1,3-dicyclohexyl-1,3,4,5,6,7-hexahydro-2H-benzo[d]imidazol-2-one **3j**[2]

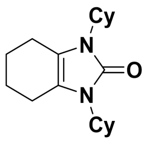

Product was isolated via column chromatography (PE/EA = 1:1) as white solid (128.4 mg, 0.43 mmol, 85 %). ^{1}H NMR (400 MHz, CD_3CN) δ 3.68 (s, 2H), 2.33 (s, 4H), 1.90 – 1.79 (m, 4H), 1.73 – 1.67 (m, 4H), 1.63 (s, 4H), 1.60 – 1.52 (m, 6H), 1.24 (d, J = 13.1 Hz, 4H), 1.08 (d, J = 13.0 Hz, 2H). ^{13}C NMR (101 MHz, CD_3CN) δ 152.80(s), 116.46(s), 53.31(s), 31.67(s), 26.54(s), 25.76(s), 22.87(s), 21.73(s).

1,3-diethyl-1,3-dihydro-2H-imidazol-2-one **4a**

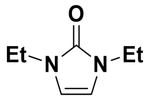

Product was isolated via column chromatography (PE/EA = 1:1) as colorless oil (38.5 mg, 0.28 mmol, 55 %). ^{1}H NMR (400 MHz, CD_3CN) δ 6.23 (s, 2H), 3.47 (d, J = 7.2 Hz, 4H), 1.10 (d, J = 7.2 Hz, 6H).^{13}C NMR (101 MHz, CD_3CN) δ 110.09(s), 38.32(s), 14.65(s). HRMS (ESI-TOF, m/z) calcd. for $C_7H_{13}N_2O$ $(M+H)^+$ 141.10224; found 141.10194.

1,3-diisopropyl-1,3-dihydro-2H-imidazol-2-one **4b**[3]

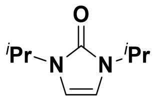

Product was isolated via column chromatography (PE/EA = 1:1) as colorless oil (52.1 mg, 0.31 mmol, 62 %). ^{1}H NMR (400 MHz, CD_3CN) δ 6.28 (s, 2H), 4.20 – 4.13 (m, 2H), 1.15 (d, J = 6.7 Hz, 12H). ^{13}C NMR (101 MHz, CD_3CN) δ 152.15(s), 107.24(s), 44.68(s), 21.70(s).

1,3-dipropyl-1,3-dihydro-2H-imidazol-2-one **4c**

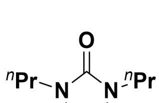

Product was isolated via column chromatography (PE/EA = 1:1) as colorless oil (35.3 mg, 0.21 mmol,

42 %). ^{1}H NMR (400 MHz, CD_3CN) δ 6.22 (s, 2H), 3.42 – 3.38 (m, 4H), 1.54 (q, J = 7.2 Hz, 4H), 0.79 (d, J = 7.4 Hz, 6H). ^{13}C NMR (101 MHz, CD_3CN) δ 110.61(s), 45.06(s), 23.15(s), 10.90(s). HRMS (ESI-TOF, m/z) calcd. for $C_9H_{17}N_2O$ $(M+H)^+$ 169.13354; found 169.13304. HRMS (ESI-TOF, m/z) calcd. for $C_9H_{17}N_2O$ $(M+H)^+$ 169.13354; found 169.13304.

1,3-dibutyl-1,3-dihydro-2H-imidazol-2-one **4d**[2]

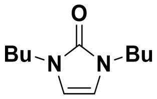

Product was isolated via column chromatography (PE/EA = 1:1) as white solid (45.1 mg, 0.23 mmol, 46 %). ^{1}H NMR (400 MHz, CD_3CN) δ 6.32 (s, 2H), 3.54 (t, J = 7.1 Hz, 4H), 1.63 – 1.57 (m, 4H), 1.33 – 1.28 (m, 4H), 0.93 (t, J = 7.4 Hz, 6H). ^{13}C NMR (101 MHz, CD_3CN) δ 153.41(s), 110.57(s), 43.10(s), 31.93(s), 20.01(s), 13.51(s).

1,3,4,5-tetramethyl-1,3-dihydro-2H-imidazol-2-one **4e**[4]

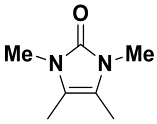

Product was isolated via column chromatography (PE/EA = 1:1) as colorless oil (60.2 mg, 0.43 mmol, 86 %). ^{1}H NMR (400 MHz, CD_3CN) δ 3.08 (s, 6H), 1.97 (s, 6H). ^{13}C NMR (101 MHz, CD_3CN) δ 153.80(s), 113.49(s), 27.05(s), 8.11(s).

1,3-diethyl-4,5-dimethyl-1,3-dihydro-2H-imidazol-2-one **4f**[5]

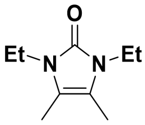

Product was isolated via column chromatography (PE/EA = 1:1) as colorless oil (51.2 mg, 0.31 mmol, 61 %). ^{1}H NMR (400 MHz, CD_3CN) δ 3.47 (q, J = 7.2 Hz, 4H), 1.89 (s, 6H), 1.03 (t, J = 7.2 Hz, 6H). ^{13}C NMR (101 MHz, CD_3CN) δ 113.04(s), 35.77(s), 15.03(s), 7.99(s).

1,3-diisopropyl-4,5-dimethyl-1,3-dihydro-2H-imidazol-2-one **4g**[6]

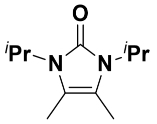

Product was isolated via column chromatography (PE/EA = 1:1) as white solid (62.7 mg, 0.32 mmol, 64 %). ^{1}H NMR (400 MHz, CD_3CN) δ 4.20 (p, J = 6.9 Hz, 2H), 1.99 (s, 6H), 1.37 (d, J = 7.0 Hz, 12H).^{13}C NMR (101 MHz, CD_3CN) δ 152.82(s), 113.25(s), 45.16(s), 20.76(s), 9.04(s).

4,5-dimethyl-1,3-dipropyl-1,3-dihydro-2H-imidazol-2-one **4h**

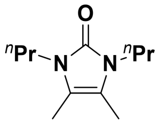

Product was isolated via column chromatography (PE/EA = 1:1) as white solid (77.4 mg, 0.40 mmol, 79 %). ^{1}H NMR (400 MHz, CD_3CN) δ 3.30 – 3.22 (m, 4H), 1.76 (s, 6H), 1.35 (q, J = 7.4 Hz, 4H), 0.65 (t, J = 7.4 Hz, 6H). ^{13}C NMR (101 MHz, CD_3CN) δ 153.71(s), 113.24(s), 42.60(s), 23.43(s), 11.04(s), 8.21(s). HRMS (ESI-TOF, m/z) calcd. for $C_{11}H_{21}N_2O$ $(M+H)^+$ 197.16484; found 197.16401.

1,3-dibutyl-4,5-dimethyl-1,3-dihydro-2H-imidazol-2-one **4i**

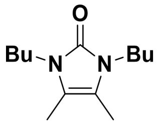

Product was isolated via column chromatography (PE/EA = 1:1) as white solid (104.2 mg, 0.47 mmol, 93 %). ^{1}H NMR (400 MHz, CD_3CN) δ 3.55 – 3.49 (m, 4H), 1.98 (s, 6H), 1.56 – 1.49 (m, 4H), 1.34 – 1.28 (m, 4H), 0.93 (t, J = 7.4 Hz, 6H). ^{13}C NMR (101 MHz, CD_3CN) δ 153.58(s), 113.28(s), 40.82(s), 32.38(s), 20.27(s), 13.71, 8.22(s). HRMS (ESI-TOF, m/z) calcd. for $C_{13}H_{25}N_2O$ $(M+H)^+$ 225.19614; found 225.19508.

4,5-dimethyl-1,3-diphenyl-1,3-dihydro-2H-imidazol-2-one **4j**

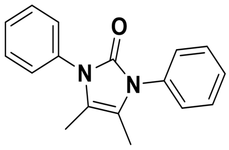

Product was isolated via column chromatography (PE/EA = 1:1) as white solid (70.0 mg, 0.27 mmol, 53 %). ^{1}H NMR (400 MHz, CD_3CN) δ 7.55 – 7.51 (m, 4H), 7.46 – 7.42 (m, 2H), 7.41 – 7.38 (m, 4H),

1.95 (s, 6H). ^{13}C NMR (101 MHz, CD_3CN) δ 152.90(s), 136.38(s), 129.65(s), 128.35(s), 128.10(s), 115.12(s), 9.36(s). HRMS (ESI-TOF, m/z) calcd. for $C_{17}H_{16}N_2O$ $(M+H)^+$ 265.1335; found 265.1340.

1-benzyl-4,5-dimethyl-1,3-dihydro-2H-imidazol-2-one **4k**

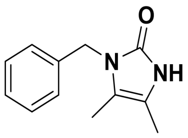

Product was isolated via column chromatography (PE/EA = 1:1) as white solid (46.5 mg, 0.23 mmol, 46 %). ^1H NMR (400 MHz, MeOD) δ 7.32 – 7.30 (m, 5H), 4.84 (s, 2H), 4.31 (s, 1H), 1.98 (d, J = 1.2 Hz, 3H), 1.85 (d, J = 1.2 Hz, 3H). ^{13}C NMR (101 MHz, MeOD) δ 137.75(s), 128.33(s), 128.09(s), 127.02(s), 126.82(s), 126.64(s), 114.57(s), 112.60(s), 43.54(s), 7.59(s), 7.03(s). HRMS (ESI-TOF, m/z) calcd. for $C_{12}H_{14}N_2O$ $(M+H)^+$ 203.1179; found 203.1183.

1,3-dibutyl-4-phenyl-1,3-dihydro-2H-imidazol-2-one **4l**[2]

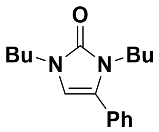

Product was isolated via column chromatography (PE/EA = 1:1) as white solid (126.5 mg, 0.47 mmol, 93 %). ^1H NMR (400 MHz, CD_3CN) δ 7.46 – 7.43 (m, 2H), 7.42 (d, J = 1.9 Hz, 2H), 7.40 (s, 1H), 6.43 (s, 1H), 3.74 (d, J = 7.3 Hz, 2H), 3.63 (d, J = 7.1 Hz, 2H), 1.66 (s, 2H), 1.43 – 1.39 (m, 2H), 1.33 (s, 2H), 1.16 (d, J = 7.6 Hz, 2H), 0.96 (s, 3H), 0.78 (d, J = 7.4 Hz, 3H). ^{13}C NMR (101 MHz, CD_3CN) δ 154.18(s), 131.03(s), 129.66(s), 129.36(s), 128.27(s), 128.14(s), 124.32(s), 109.16(s), 43.08(s), 41.61(s), 31.87(s), 31.65(s), 20.04(s), 19.91(s), 13.53(s), 13.41(s).

4-(4-fluorophenyl)-1,3-dimethyl-1,3-dihydro-2H-imidazol-2-one **4m**

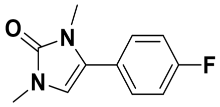

Product was isolated via column chromatography (PE/EA = 1:1) as white solid (81.4 mg, 0.40 mmol, 79 %). ^1H NMR (400 MHz, CD_3CN) δ 7.46 – 7.39 (m, 2H), 7.19 (t, J = 8.9 Hz, 2H), 6.42 (s, 1H), 3.23 (s, 3H). ^{13}C NMR (101 MHz, CD_3CN) δ 164.06(s), 161.63(s), 154.46(s), 130.17(s), 130.09(s), 126.98,

126.95(s), 123.77(s), 116.29(s), 116.07(s), 109.88(s), 30.13(s), 29.02(s). ^{19}F NMR (376 MHz, CD_3CN) δ -115.84(s). HRMS (ESI-TOF, m/z) calcd. for $C_{11}H_{11}FN_2O$ $(M+H)^+$ 207.0928; found 207.0932.

4-(4-methoxyphenyl)-1,3-dimethyl-1,3-dihydro-2H-imidazol-2-one **4n**

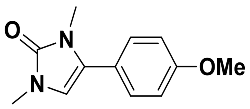

Product was isolated via column chromatography (PE/EA = 1:1) as white solid (90.5 mg, 0.42 mmol, 83 %). ^{1}H NMR (400 MHz, CD_3CN) δ 7.17 (d, J = 8.8 Hz, 2H), 6.82 (d, J = 8.8 Hz, 2H), 6.16 (s, 1H), 3.66 (s, 3H), 3.03 (d, J = 13.5 Hz, 6H).^{13}C NMR (101 MHz, CD_3CN)^{13}C NMR (101 MHz, CD_3CN) δ 159.99(s), 129.58(s), 124.51(s), 122.94(s), 114.72(s), 108.87(s), 55.58(s), 30.03(s), 28.93(s). HRMS (ESI-TOF, m/z) calcd. for $C_{12}H_{14}N_2O_2$ $(M+H)^+$ 219.1128; found 219.1332.

1,3-dimethyl-4-(pyridin-2-yl)-1,3-dihydro-2H-imidazol-2-one **4o**

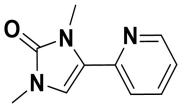

Product was isolated via column chromatography (PE/EA = 1:1) as white solid (75.6 mg, 0.40 mmol, 80 %). ^{1}H NMR (400 MHz, CD_3CN) δ 8.66 – 8.53 (m, 2H), 7.79 (d, J = 8.0 Hz, 1H), 7.41 (dd, J = 8.0, 4.5 Hz, 1H), 6.56 (s, 1H), 3.24 (d, J = 5.0 Hz, 6H). ^{13}C NMR (101 MHz, CD_3CN) δ 149.19(s), 148.65(s), 135.06(s), 126.74(s), 124.13(s), 121.57(s), 111.03(s), 30.24(s), 29.11(s). HRMS (ESI-TOF, m/z) calcd. for $C_{10}H_{11}N_3O$ $(M+H)^+$ 190.0902; found 190.0973.

1,3,4-trimethyl-5-phenyl-1,3-dihydro-2H-imidazol-2-one **4p**

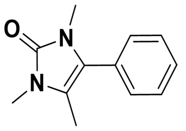

Product was isolated via column chromatography (PE/EA = 1:1) as white solid (88.9 mg, 0.44 mmol, 88 %). ^{1}H NMR (400 MHz, CD_3CN) δ 7.49 – 7.30 (m, 5H), 3.20 (s, 3H), 3.08 (s, 3H), 2.05 (s, 3H).^{13}C NMR (101 MHz, CD_3CN) δ 130.21(s), 130.06(s), 129.17(s), 129.17(s), 128.14(s), 119.73(s), 116.74(s), 28.73(s), 27.34(s), 8.96(s). HRMS (ESI-TOF, m/z) calcd. for $C_{12}H_{14}N_2O$ $(M+H)^+$ 203.1106; found 203.1184.

1,3-dimethyl-1,3,4,5,6,7-hexahydro-2H-benzo[d]imidazol-2-one **4q**[4]

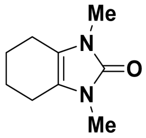

Product was isolated via column chromatography (PE/EA = 1:1) as white solid (63.9 mg, 0.39 mmol, 77 %). ^{1}H NMR (400 MHz, CD_3CN) δ 3.06 (s, 6H), 2.33 (s, 4H), 1.78 (s, 4H). ^{13}C NMR (101 MHz, CD_3CN) δ 153.99(s), 116.94(s), 26.83(s), 22.89(s), 19.83(s).

1,3-dibutyl-1,3,4,5,6,7-hexahydro-2H-benzo[d]imidazol-2-one **4r**[2]

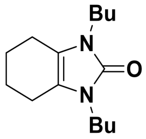

Product was isolated via column chromatography (PE/EA = 1:1) as white solid (123.8 mg, 0.50 mmol, 99 %). ^{1}H NMR (400 MHz, CD_3CN) δ 3.33 (t, J = 7.2 Hz, 4H), 2.19 (td, J = 3.8, 1.9 Hz, 4H), 1.60 (p, J = 3.4 Hz, 4H), 1.37 (tdd, J = 7.8, 7.0, 5.8 Hz, 4H), 1.18 – 1.11 (m, 4H), 0.76 (t, J = 7.4 Hz, 6H). ^{13}C NMR (101 MHz, CD_3CN) δ 153.66(s), 116.58(s), 40.72(s), 32.35(s), 22.95(s), 20.25(s), 20.22(s), 13.67(s).

1,3-dibenzyl-4,5-dimethyl-1,3-dihydro-2H-imidazol-2-one **4s**[7]

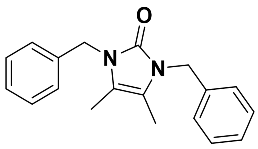

Product was isolated via column chromatography (PE/EA = 1:1) as white solid (90.5 mg, 0.31 mmol, 62 %). ^{1}H NMR (400 MHz, CD_3CN) δ 7.38 – 7.25 (m, 10H), 4.85 (s, 4H), 1.89 (s, 6H). ^{13}C NMR (101 MHz, CD_3CN) δ 154.29(s), 139.37(s), 129.20(s), 127.77(s), 127.31(s), 113.98(s), 44.51(s), 8.38(s).

4,5-dimethyl-1,3-bis(4-methylbenzyl)-1,3-dihydro-2H-imidazol-2-one **4t**

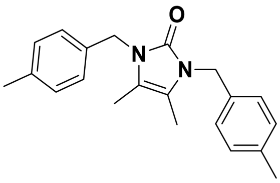

Product was isolated via column chromatography (PE/EA = 1:1) as white solid (86.4 mg, 0.27 mmol, 54 %). ^{1}H NMR (400 MHz, CD_3CN) δ 7.18 (d, *J* = 8.3 Hz, 4H), 7.13 (d, *J* = 8.2 Hz, 4H), 4.79 (s, 4H), 2.33 (s, 6H), 1.87 (s, 6H). ^{13}C NMR (101 MHz, CD_3CN) δ 154.29(s), 137.47(s), 136.42(s), 129.77(s), 127.34(s), 113.89(s), 44.24(s), 20.68(s), 8.36(s).

1,3-bis(4-methoxybenzyl)-4,5-dimethyl-1,3-dihydro-2H-imidazol-2-one **4u**

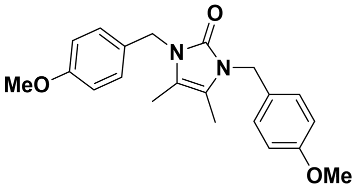

Product was isolated via column chromatography (PE/EA = 1:1) as white solid (107.4 mg, 0.31 mmol, 61 %). ^{1}H NMR (400 MHz, CD_3CN) δ 7.20 – 7.17 (m, 4H), 6.92 – 6.89 (m, 4H), 4.76 (s, 4H), 3.78 (s, 6H), 1.88 (s, 6H). ^{13}C NMR (101 MHz, CD_3CN) δ 159.49(s), 154.27(s), 131.40(s), 128.74(s), 114.51(s), 113.84(s), 55.47(s), 43.93(s), 8.37(s). HRMS (ESI-TOF, m/z) calcd. for $C_{21}H_{25}N_2O_3$ $(M+H)^+$ 353.18597; found 353.21546.

1,3-bis(4-fluorobenzyl)-4,5-dimethyl-1,3-dihydro-2H-imidazol-2-one **4v**

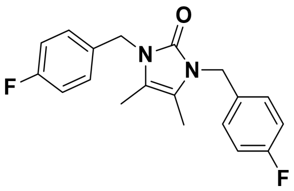

Product was isolated via column chromatography (PE/EA = 1:1) as white solid (86.9 mg, 0.27 mmol, 53 %). ^{1}H NMR (400 MHz, CD_3CN) δ 7.30 – 7.24 (m, 4H), 7.14 – 7.06 (m, 4H), 4.81 (s, 4H), 1.88 (s, 6H). ^{13}C NMR (101 MHz, CD_3CN) δ 163.72(s), 161.30(s), 154.19(s), 135.43(s), 135.40(s), 129.38(s), 129.30(s), 115.96(s), 115.75(s), 113.98(s), 43.86(s), 8.34(s). ^{19}F NMR (376 MHz, CD_3CN) δ -117.17(s). HRMS (ESI-TOF, m/z) calcd. for $C_{19}H_{19}F_2N_2O$ $(M+H)^+$ 329.14600; found 329.17636.

1,3-bis(4-chlorobenzyl)-4,5-dimethyl-1,3-dihydro-2H-imidazol-2-one **4w**

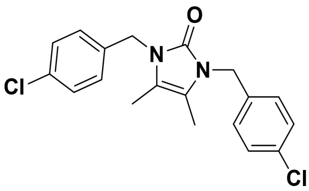

Product was isolated via column chromatography (PE/EA = 1:1) as white solid (90.0 mg, 0.25 mmol, 50 %). ^{1}H NMR (400 MHz, CD_3CN) δ 7.37 (d, J = 8.5 Hz, 4H), 7.22 (d, J = 8.5 Hz, 4H), 4.81 (s, 4H), 1.88 (s, 6H). ^{13}C NMR (101 MHz, CD_3CN) δ 154.18(s), 138.19(s), 133.03(s), 129.20(s), 129.05(s), 114.03(s), 43.91(s), 8.32(s).

4,5-dimethyl-1,3-bis(4-(trifluoromethyl)benzyl)-1,3-dihydro-2H-imidazol-2-one **4x**

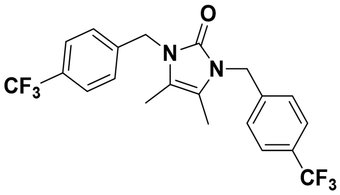

Product was isolated via column chromatography (PE/EA = 1:1) as white solid (102.7 mg, 0.24 mmol, 48 %). ^{1}H NMR (400 MHz, CD_3CN) δ 7.69 (d, J = 8.1 Hz, 4H), 7.44 – 7.40 (m, 4H), 4.93 (s, 4H), 1.89 (s, 6H). ^{13}C NMR (101 MHz, CD_3CN) δ 127.92(s), 126.16(s), 126.13(s), 117.90(s), 114.19(s), 44.23(s), 8.33(s). ^{19}F NMR (376 MHz, CD_3CN) δ -62.95(s).

1,3-bis(4-methylbenzyl)urea **1n**[8]

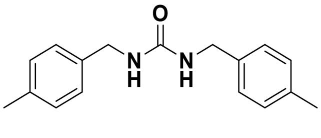

Product was isolated via column chromatography (PE/EA = 1:1) as white solid (257.3 mg, 0.96 mmol, 48 %). ^{1}H NMR (400 MHz, DMSO) δ 7.23 – 7.07 (m, 8H), 4.18 (d, J = 6.0 Hz, 2H), 3.57 (s, 4H), 2.27 (s, 5H). ^{13}C NMR (101 MHz, DMSO) δ 158.56(s), 138.27(s), 136.01(s), 129.21(s), 129.13(s), 127.46(s), 43.18(s), 21.11(s).

1,3-bis(4-methoxybenzyl)urea **1o**[8]

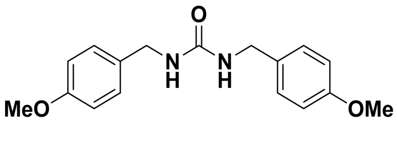

Product was isolated via column chromatography (PE/EA = 1:1) as white solid (468.0 mg, 1.56 mmol,

78 %). ^{1}H NMR (400 MHz, DMSO-d_6) δ 7.17 (d, J = 8.6 Hz, 4H), 6.87 (d, J = 8.6 Hz, 4H), 6.30 (t, J = 6.0 Hz, 2H), 4.15 (d, J = 5.9 Hz, 4H). ^{13}C NMR (101 MHz, DMSO-d_6) δ 158.54(s), 158.47(s), 133.26(s), 128.79(s), 114.09(s), 55.50(s), 42.89(s).

1,3-bis(4-fluorobenzyl)urea **1p**[8]

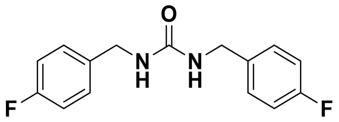

Product was isolated via column chromatography (PE/EA = 1:1) as white solid (358.8 mg, 1.30 mmol, 65 %). ^{1}H NMR (400 MHz, DMSO) δ 7.32 – 7.25 (m, 4H), 7.16 – 7.09 (m, 4H), 6.48 (t, J = 6.1 Hz, 2H), 4.21 (d, J = 6.0 Hz, 4H).^{13}C NMR (101 MHz, DMSO) δ 162.72(s), 160.31(s), 158.49(s), 137.57(s), 137.54(s), 129.41(s), 129.33(s), 115.44(s), 115.23(s), 42.72(s). ^{19}F NMR (376 MHz, DMSO) δ -116.59(s).

1,3-bis(4-chlorobenzyl)urea **1q**[8]

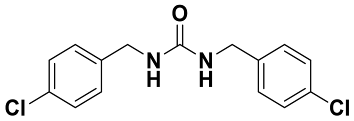

Product was isolated via column chromatography (PE/EA = 1:1) as white solid (388.1 mg, 1.26 mmol, 63 %). ^{1}H NMR (400 MHz, DMSO) δ 7.36 (s, 4H), 7.26 (d, J = 8.3 Hz, 4H), 6.58 (t, J = 6.1 Hz, 2H), 4.21 (d, J = 6.1 Hz, 4H). ^{13}C NMR (101 MHz, DMSO) δ 158.53(s), 140.47(s), 131.50(s), 129.28(s), 128.58(s), 42.76(s).

1,3-bis(4-(trifluoromethyl)benzyl)urea **1r**[8]

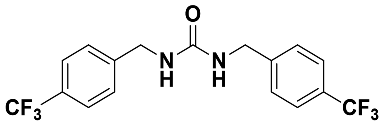

Product was isolated via column chromatography (PE/EA = 1:1) as white solid (421.1 mg, 1.12 mmol, 56 %). ^{1}H NMR (400 MHz, DMSO) δ 7.68 (d, J = 8.1 Hz, 4H), 7.47 (d, J = 8.0 Hz, 4H), 6.71 (t, J = 6.1 Hz, 2H), 4.33 (d, J = 6.1 Hz, 4H). ^{13}C NMR (101 MHz, DMSO) δ 158.56(s), 146.43(s), 146.41(s), 128.00(s), 127.88(s), 127.57(s), 126.21(s), 125.57(s), 125.53(s), 125.49(s), 125.45(s), 123.51(s), 43.09(s). ^{19}F NMR (376 MHz, DMSO) δ -60.81(s).

1-(4-fluorophenyl)ethane-1,2-diol **2m**[9]

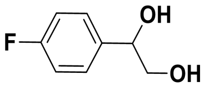

Product was isolated via column chromatography (PE/EA = 5:1) as colorless oil (379.1 mg, 2.43 mmol, 81 %). ^{1}H NMR (400 MHz, $CDCl_3$) δ 7.17 (dd, J = 8.6, 5.5 Hz, 2H), 6.92 (t, J = 8.7 Hz, 2H), 4.64 (dd, J = 8.4, 2.9 Hz, 1H), 3.92 (s, 1H), 3.56 (dd, J = 11.4, 3.4 Hz, 2H), 3.49 - 3.44 (m, 1H). ^{13}C NMR (101 MHz, $CDCl_3$) δ 163.63(s), 161.18(s), 136.18(s), 136.15(s), 127.78(s), 127.69(s), 115.48(s), 115.26(s), 74.07(s), 67.93(s), 67.92(s). ^{19}F NMR (376 MHz, CD_3CN) δ -114.28(s).

1-(4-methoxyphenyl)ethane-1,2-diol **2n**[9]

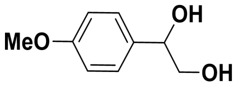

Product was isolated via column chromatography (PE/EA = 5:1) as colorless oil (428.4 mg, 2.55 mmol, 85 %). ^{1}H NMR (400 MHz, $CDCl_3$) δ 7.17 (d, *J* = 8.7 Hz, 2H), 6.79 (d, *J* = 8.7 Hz, 2H), 4.65 (dd, *J* = 8.4, 3.6 Hz, 1H), 3.71 (s, 3H), 3.60 – 3.50 (m, 2H), 3.30 (s, 1H), 2.95 (s, 1H).^{13}C NMR (101 MHz, $CDCl_3$) δ 159.33(s), 132.66(s), 127.37(s), 113.92(s), 74.31(s), 68.03(s), 55.29(s).

1-phenylpropane-1,2-diol **2p**[9]

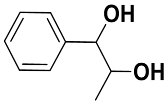

Product was isolated as white solid (261.4 mg, 1.72 mmol, 86 %). ^{1}H NMR (400 MHz, $CDCl_3$) δ 7.24 (d, J = 1.3 Hz, 5H), 4.56 (d, J = 4.1 Hz, 1H), 3.88 (dd, J = 6.4, 4.1 Hz, 1H), 0.94 (s, 3H). ^{13}C NMR (101 MHz, $CDCl_3$) δ 141.10(s), 140.40(s), 128.49(s), 128.31(s), 128.09(s), 127.73(s), 126.91(s), 126.64(s), 77.39(s), 72.21(s), 71.36(s), 18.74(s), 17.00(s).

3-hydroxy-3-methylbutan-2-one **3b**[10]

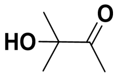

Product was isolated as colorless oil (45.9 mg, 0.45 mmol). ^{1}H NMR (400 MHz, $CDCl_3$) δ 2.05 (s, 3H), 1.17 (s, 6H).^{13}C NMR (101 MHz, $CDCl_3$) δ 212.83(s), 76.26(s), 26.08(s), 23.44(s).

2,5-dimethylbenzene-1,4-diol **3c**[11]

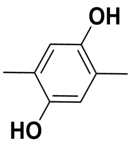

Product was isolated as white solid (52.4 mg, 0.38 mmol). ^{1}H NMR (400 MHz, MeOD) δ 6.50 (s, 2H), 4.88 (s, 2H), 2.10 (s, 6H).^{13}C NMR (101 MHz, MeOD) δ 149.01(s), 123.42(s), 118.21(s), 15.99(s).

NMR Spectra

Supplementary Figure 4. ^{1}H NMR (400 MHz, DCM-d_2) spectrum of [Mn]-III

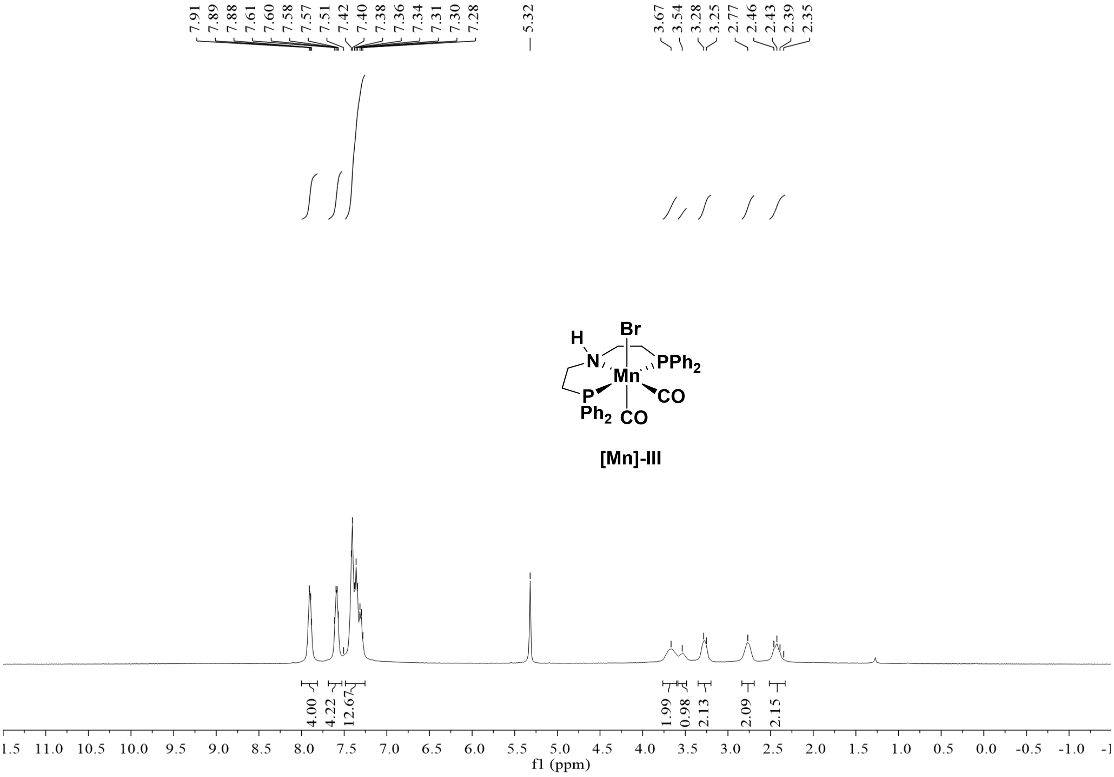

Supplementary Figure 5. ^{13}C NMR (100 MHz, DCM-d_2) spectrum of [Mn]-III

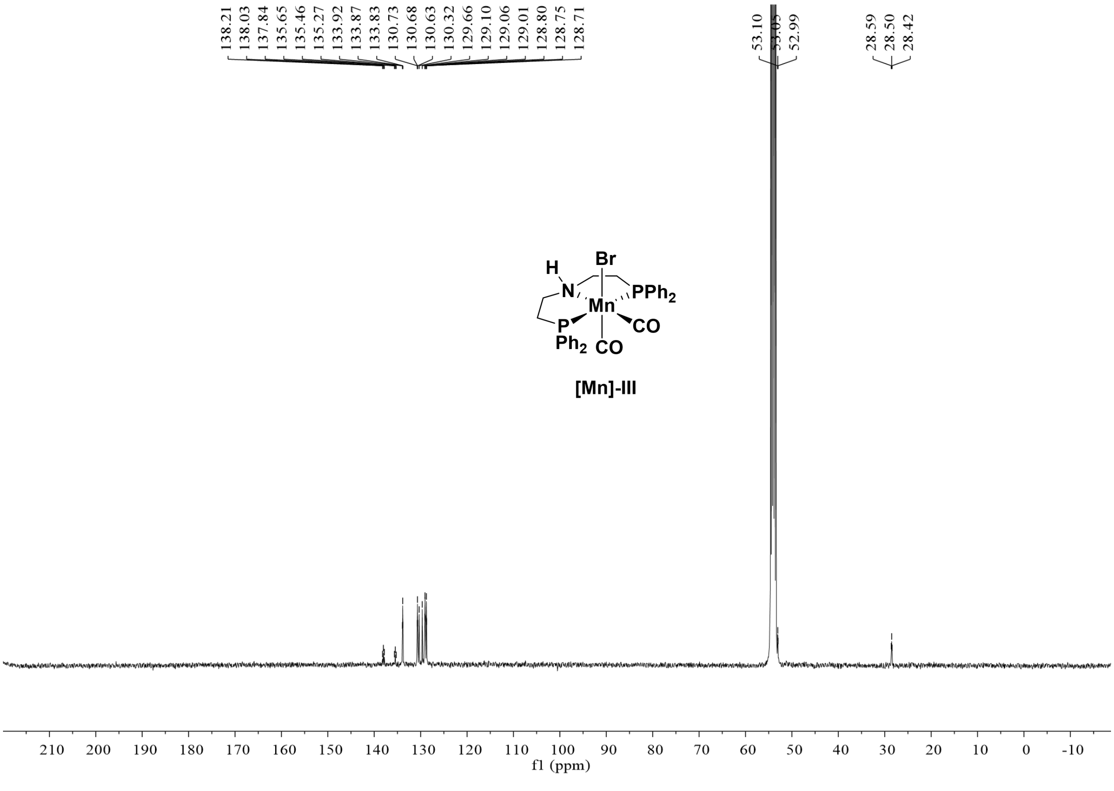

Supplementary Figure 6. ^{31}P NMR (162 MHz, DCM-d_2) spectrum of [Mn]-III

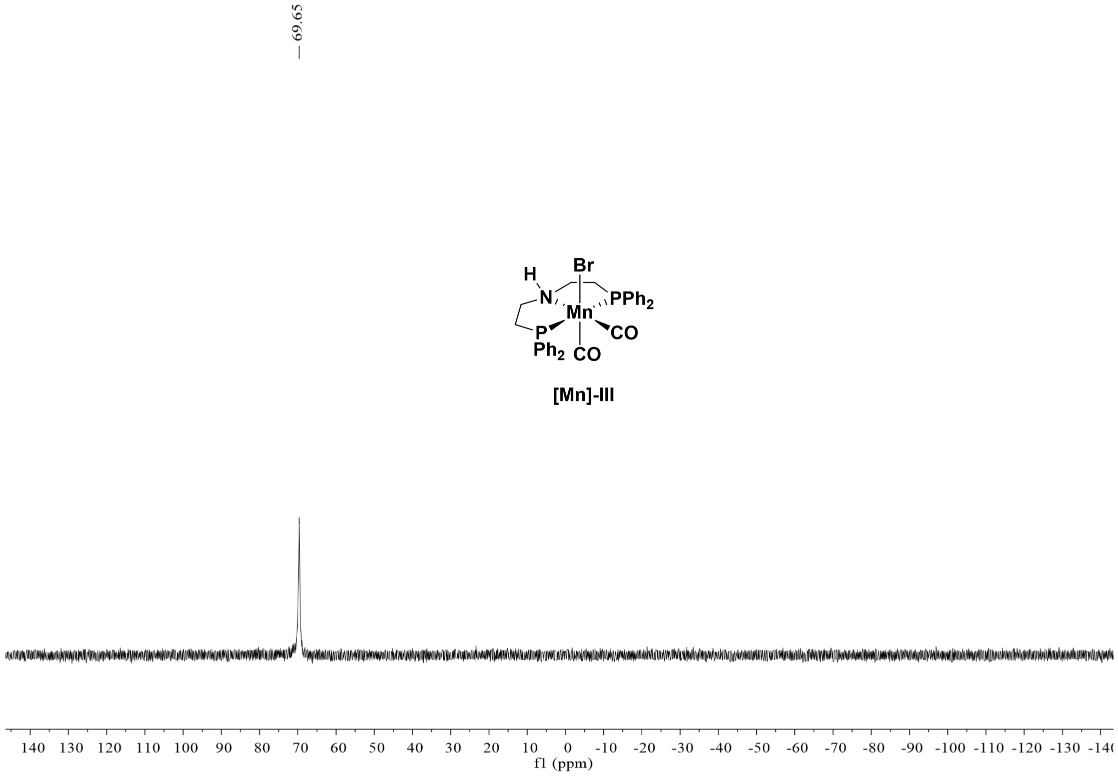

Supplementary Figure 7. ^{1}H NMR (400 MHz, $CDCl_3$) spectrum of L-V

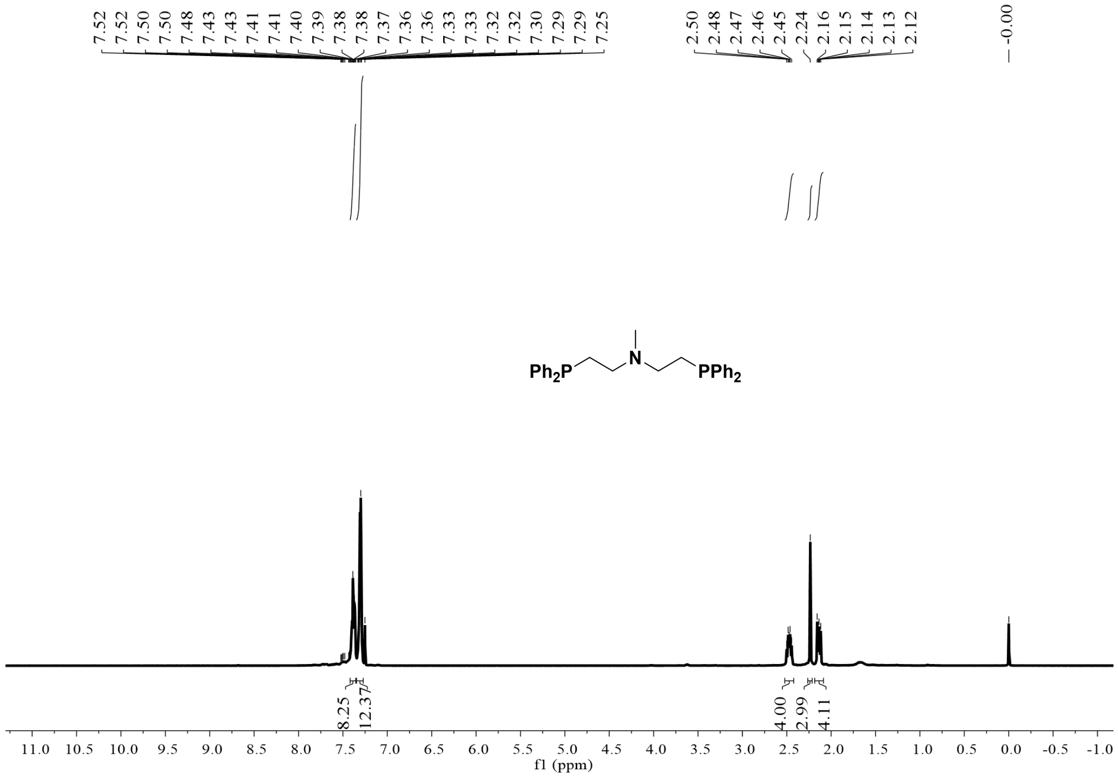

Supplementary Figure 8. ^{13}C NMR (100 MHz, $CDCl_3$) spectrum of L-V

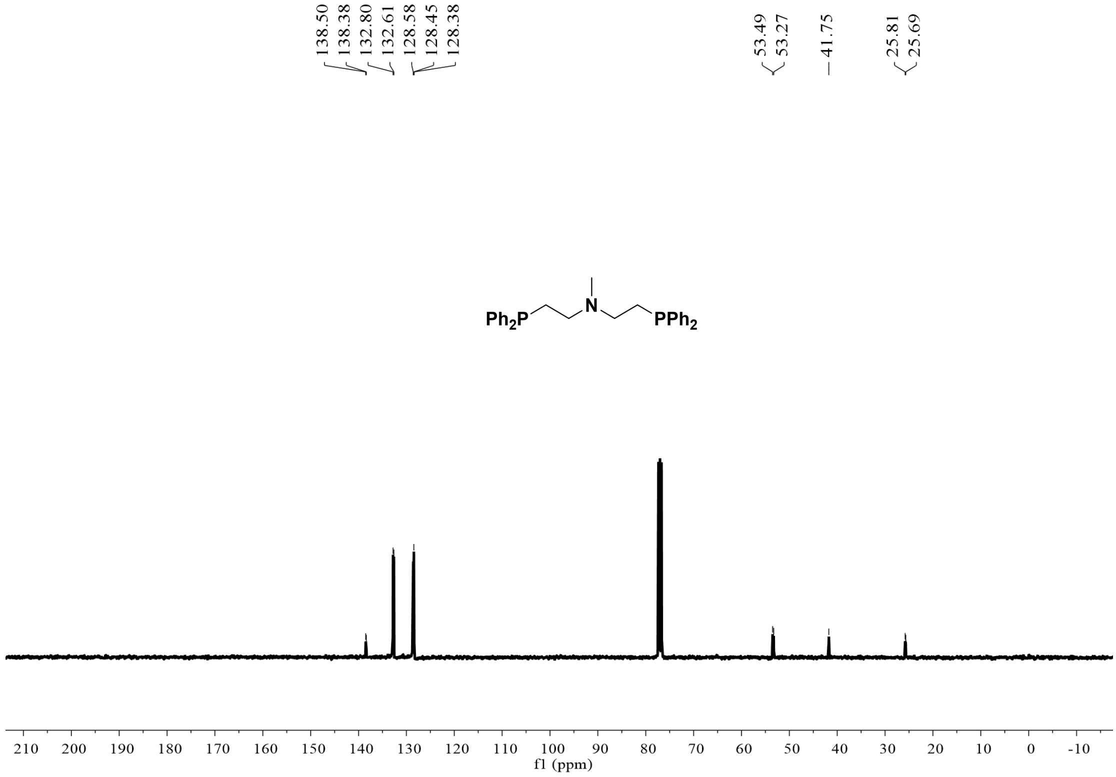

Supplementary Figure 9. ^{31}P NMR (162 MHz, $CDCl_3$) spectrum of L-V

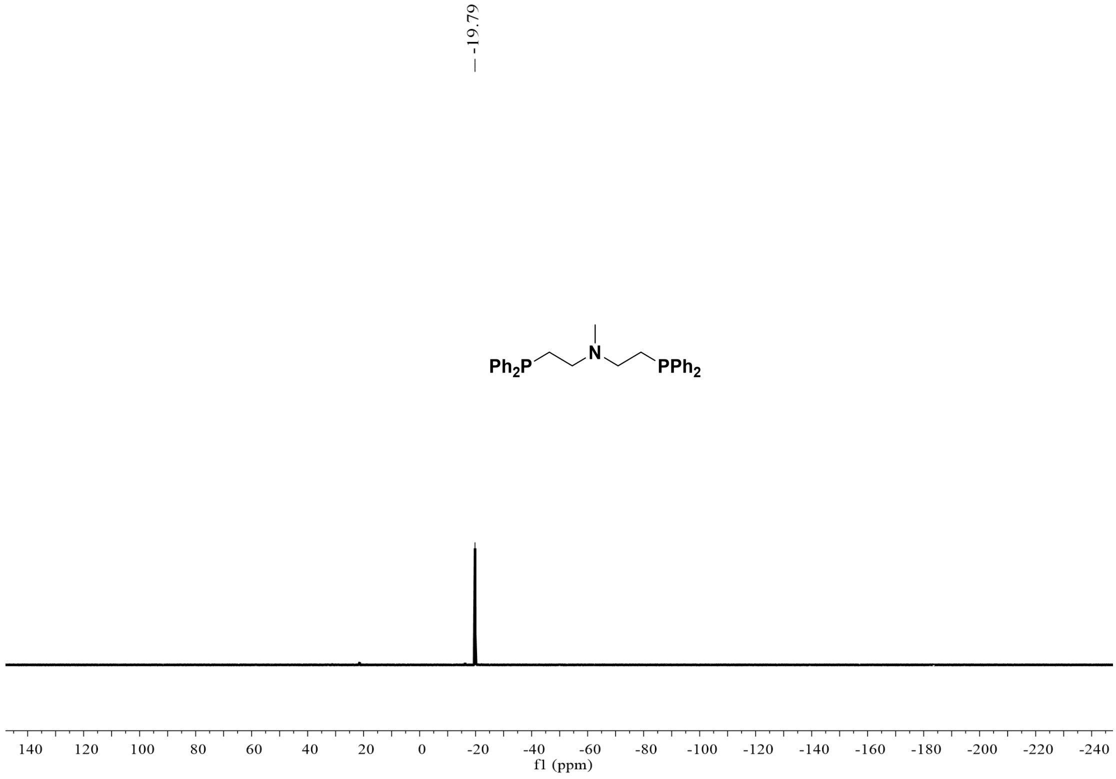

Supplementary Figure 10. ^{1}H **NMR (400 MHz, DCM-*d*$_2$) spectrum of Mn-V**

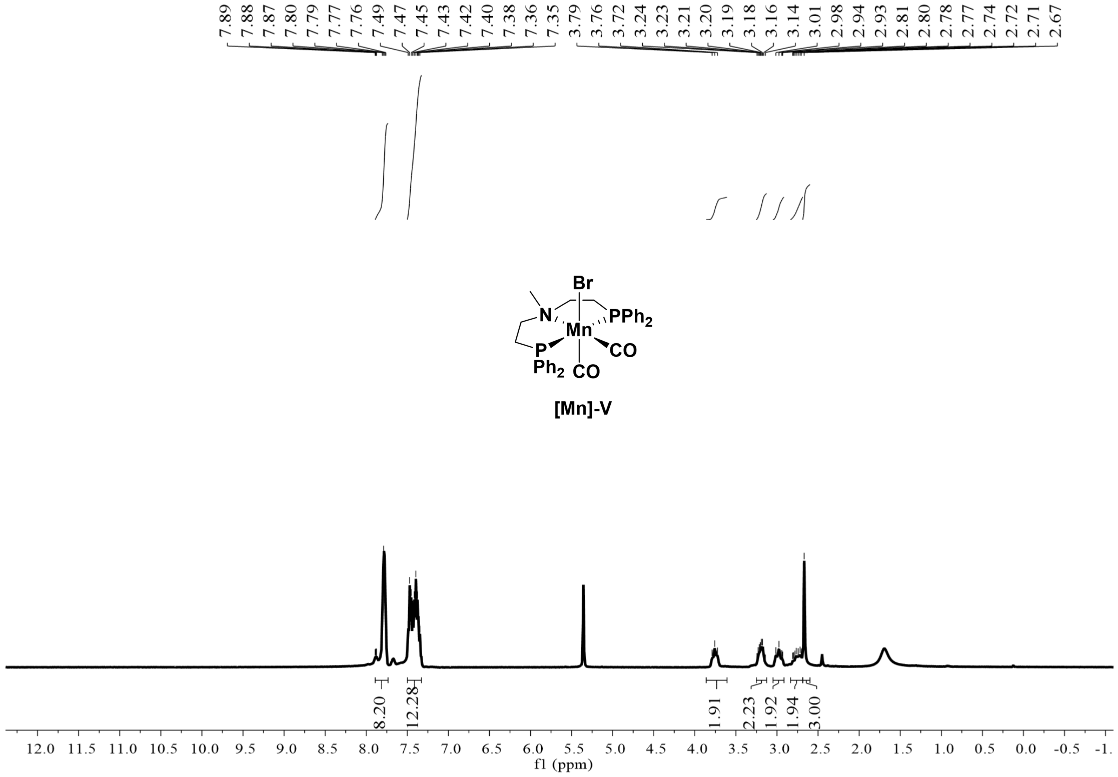

Supplementary Figure 11. ^{13}C **NMR (100 MHz, DCM-*d*$_2$) spectrum of Mn-V**

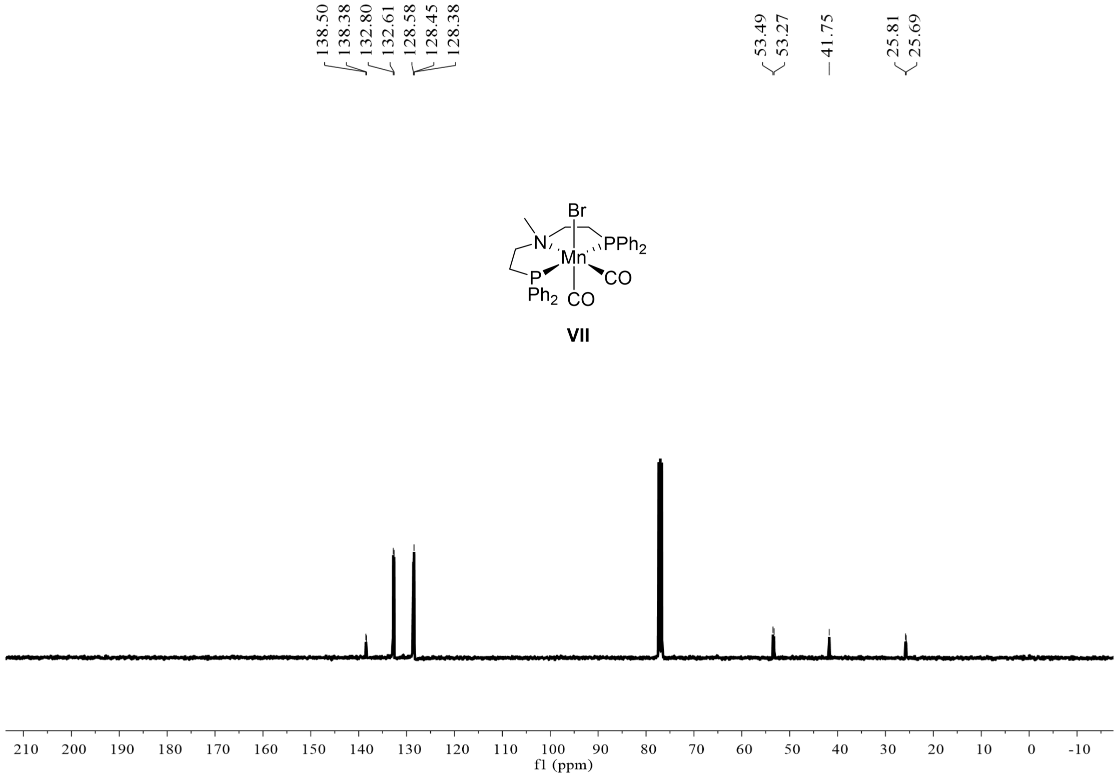

Supplementary Figure 12. ^{31}**P NMR (162 MHz, DCM-*d*$_2$) spectrum of Mn-V**

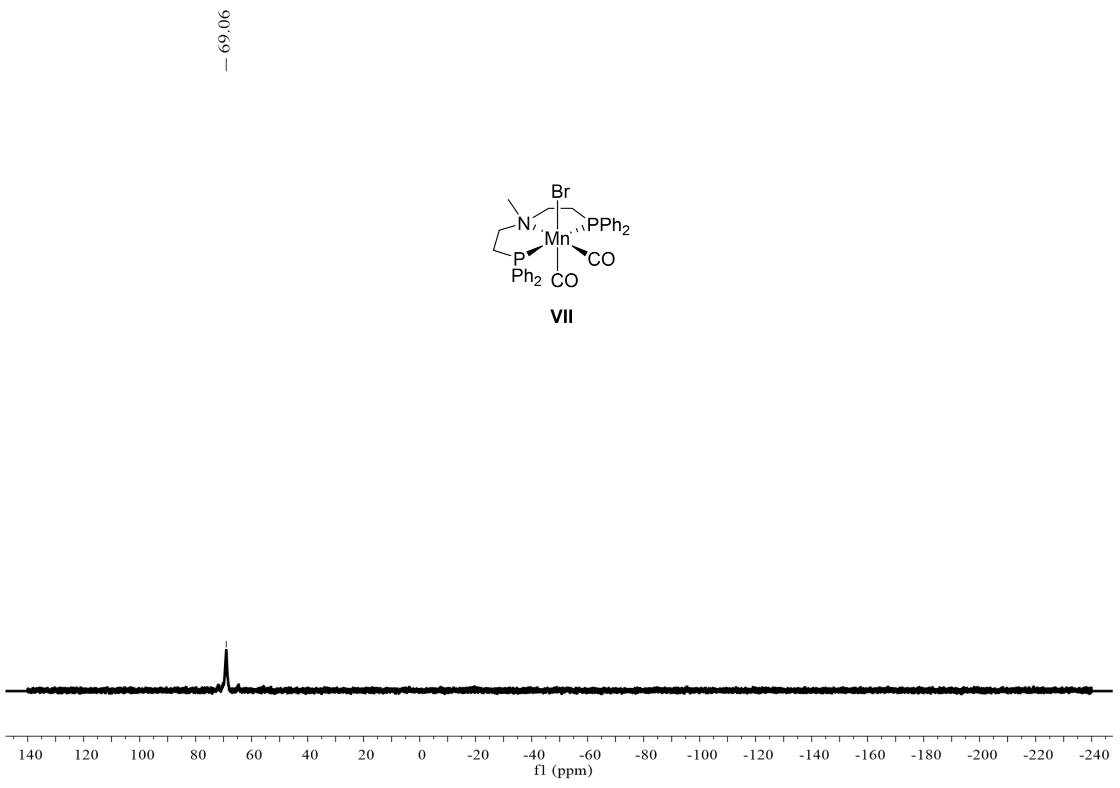

Supplementary Figure 13. ^{1}H NMR (400 MHz, CD_3CN) spectrum of 3a

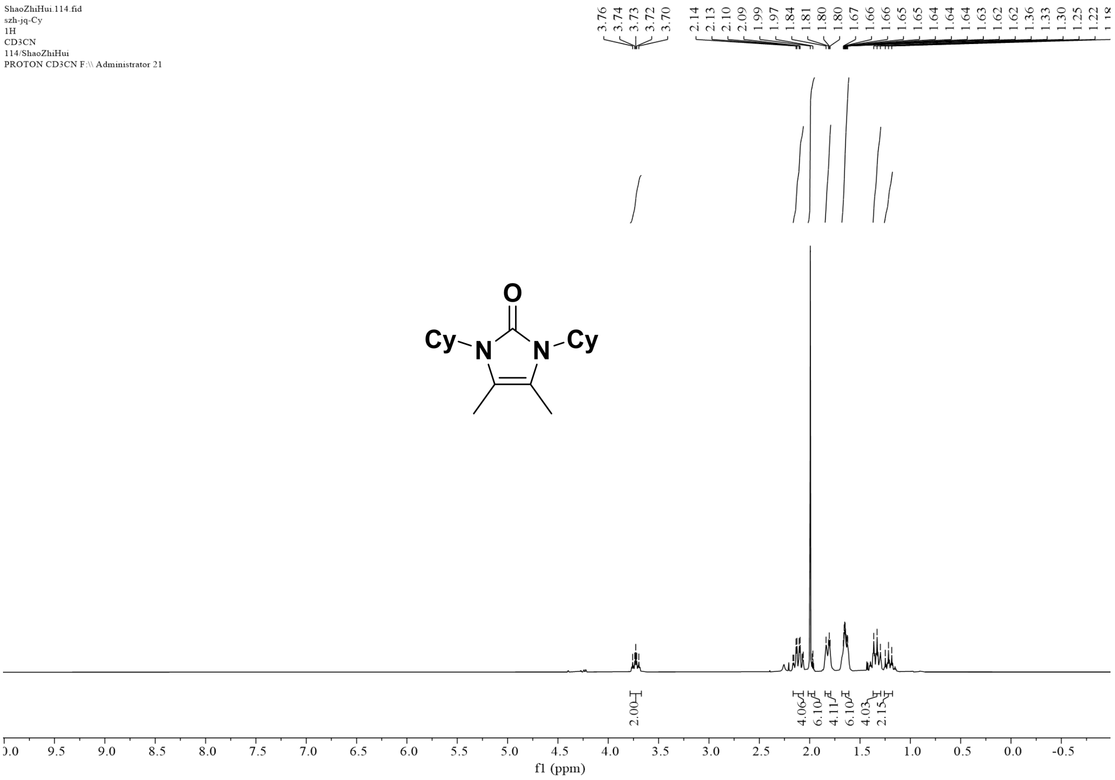

Supplementary Figure 14. ^{13}C NMR (100 MHz, CD_3CN) spectrum of 3a

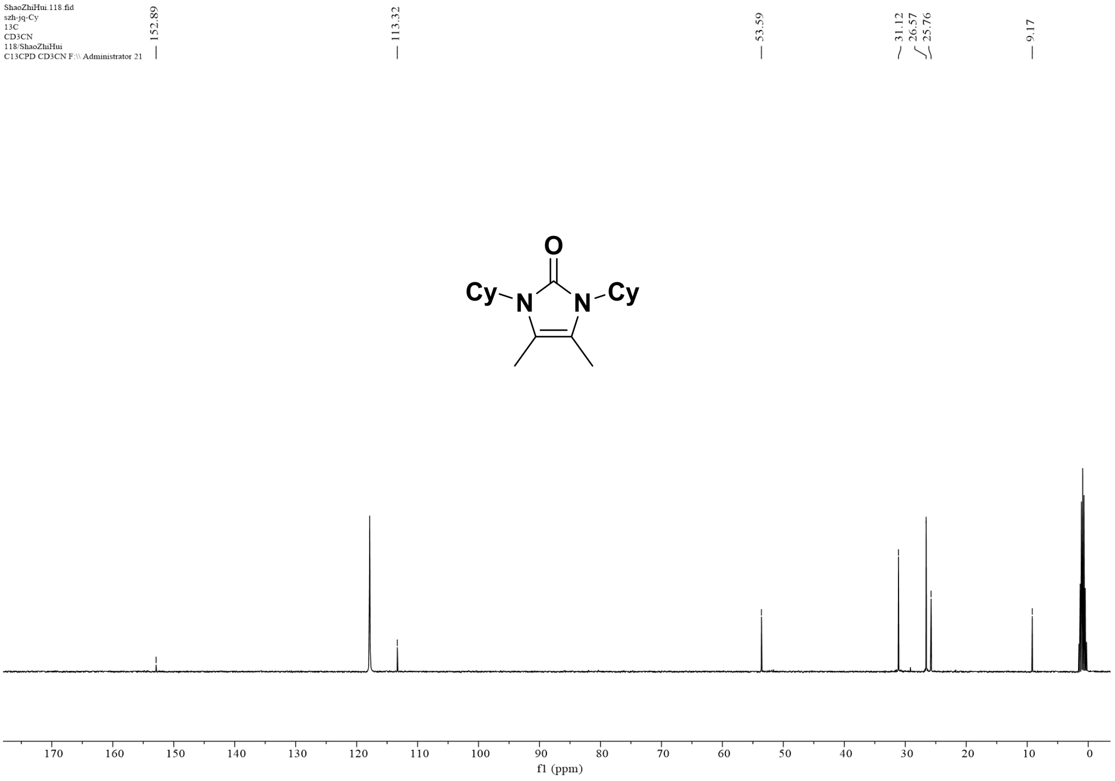

Supplementary Figure 15. ^{1}H NMR (400 MHz, CD_3CN) spectrum of 3b

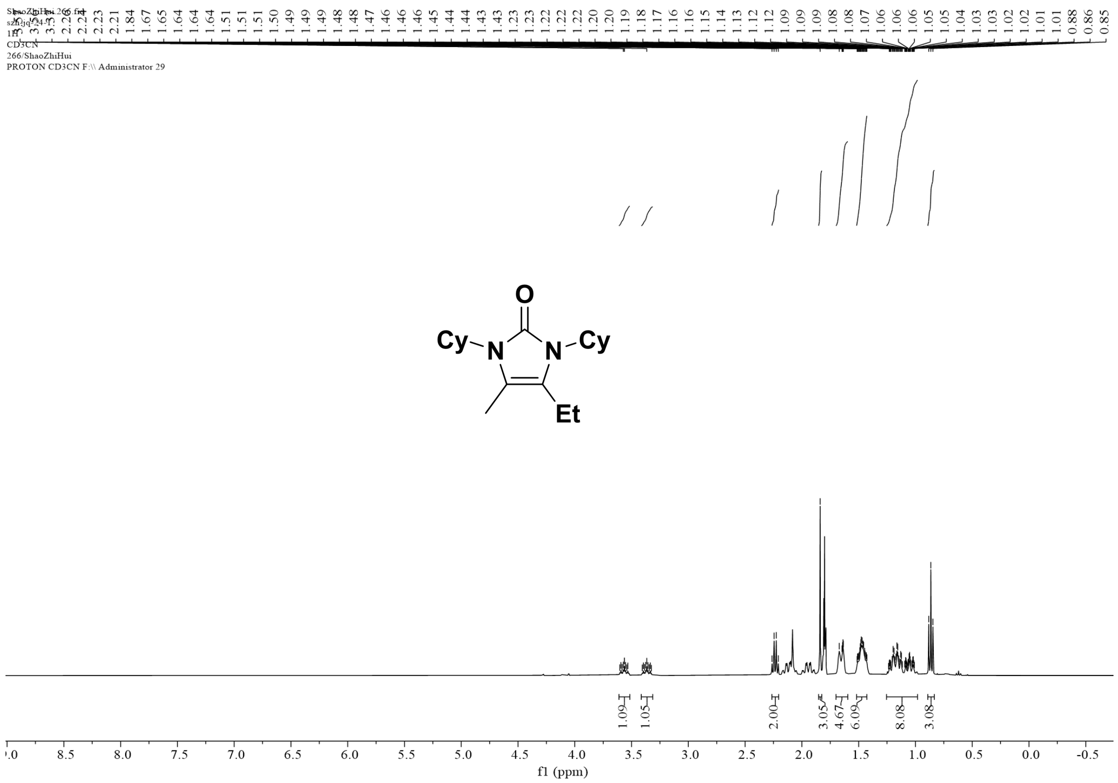

Supplementary Figure 16. ^{13}C NMR (100 MHz, CD_3CN) spectrum of 3b

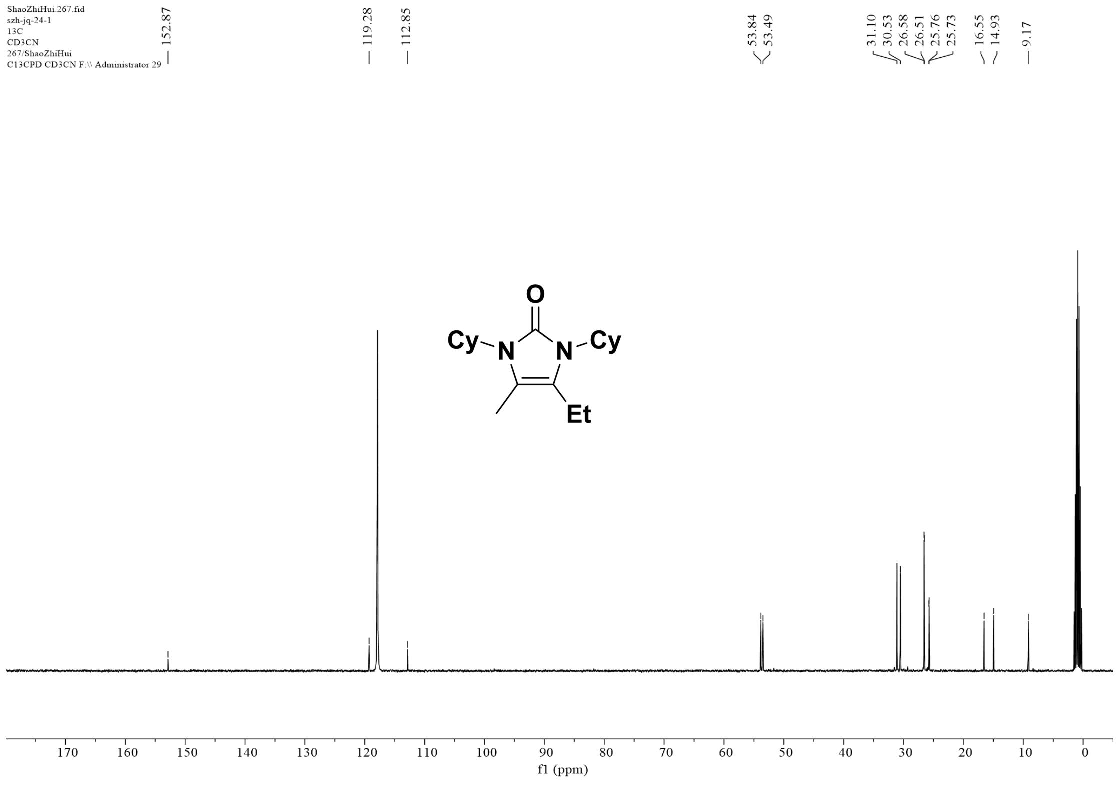

Supplementary Figure 17. ^{1}H NMR (400 MHz, CD_3CN) spectrum of 3c

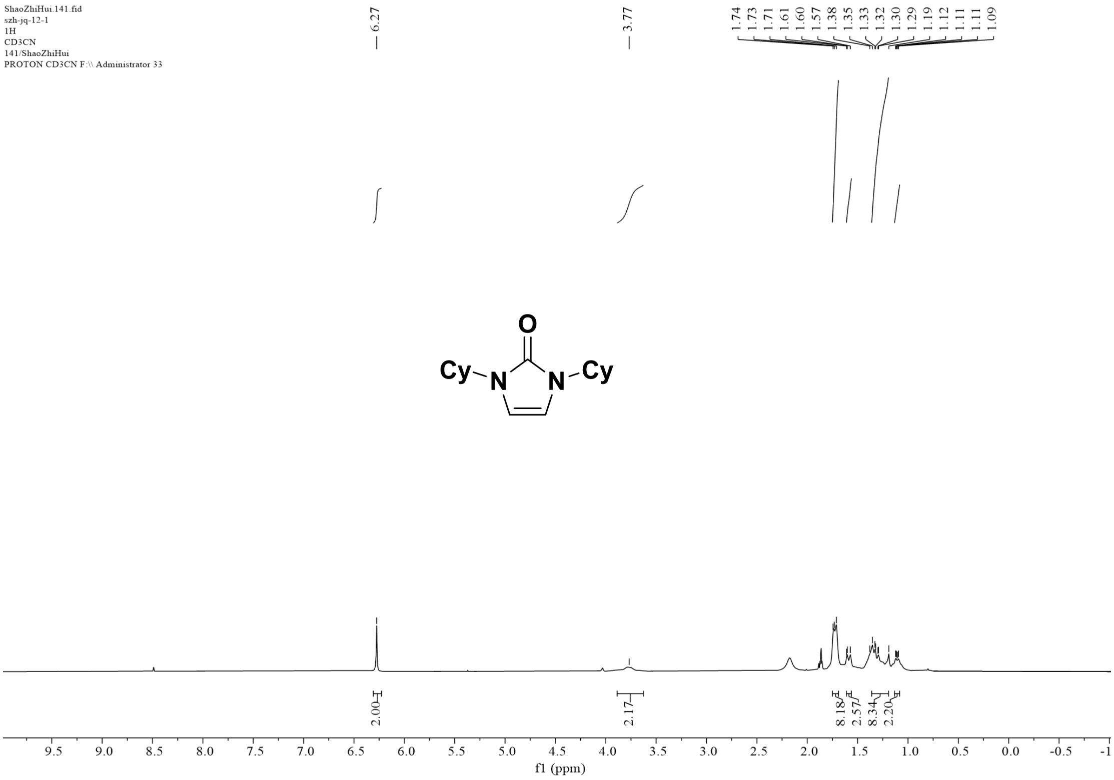

Supplementary Figure 18. ^{13}C NMR (100 MHz, CD_3CN) spectrum of 3c

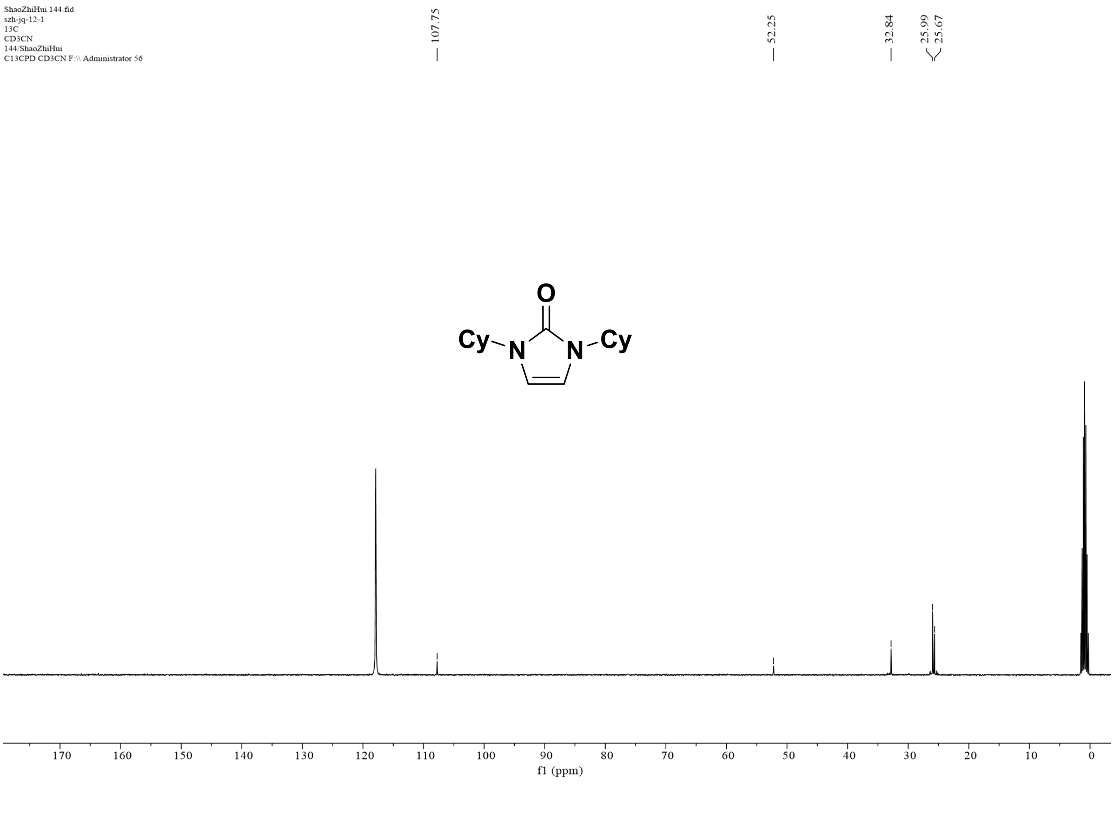

Supplementary Figure 19. ^{1}H NMR (400 MHz, CD_3CN) spectrum of 3d

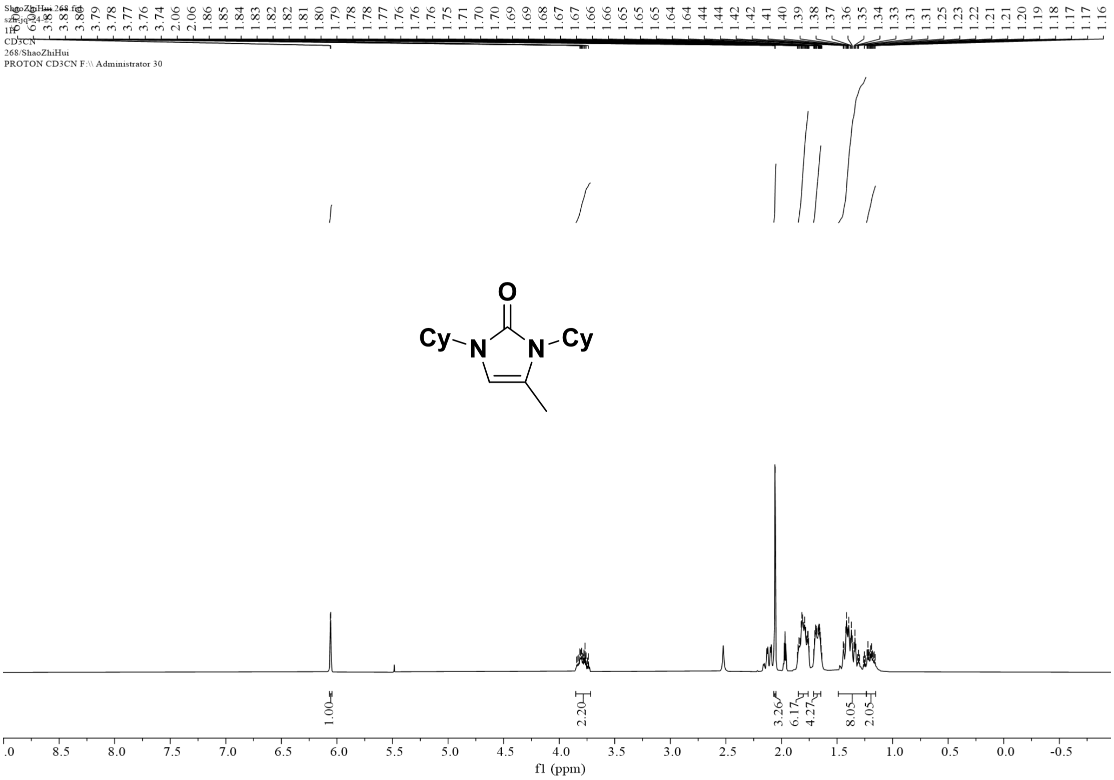

Supplementary Figure 20. ^{13}C NMR (100 MHz, CD_3CN) spectrum of 3d

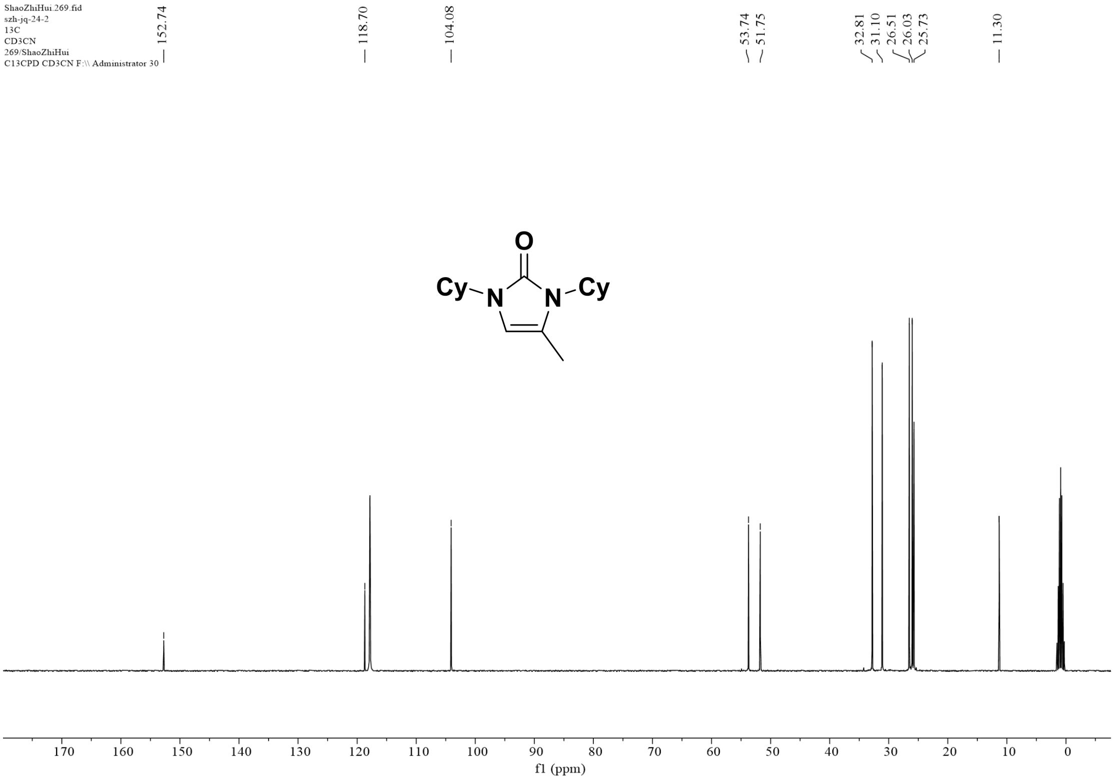

Supplementary Figure 21. ^{1}H NMR (400 MHz, CD_3CN) spectrum of 3e

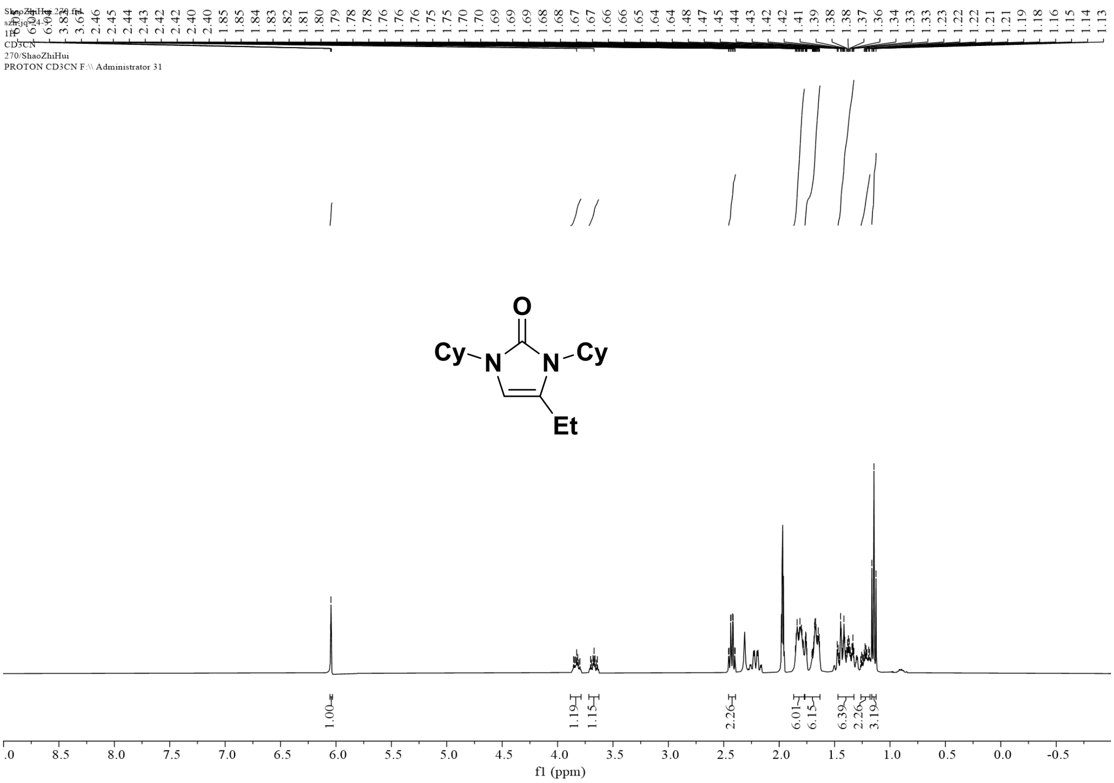

Supplementary Figure 22. ^{13}C NMR (100 MHz, CD_3CN) spectrum of 3e

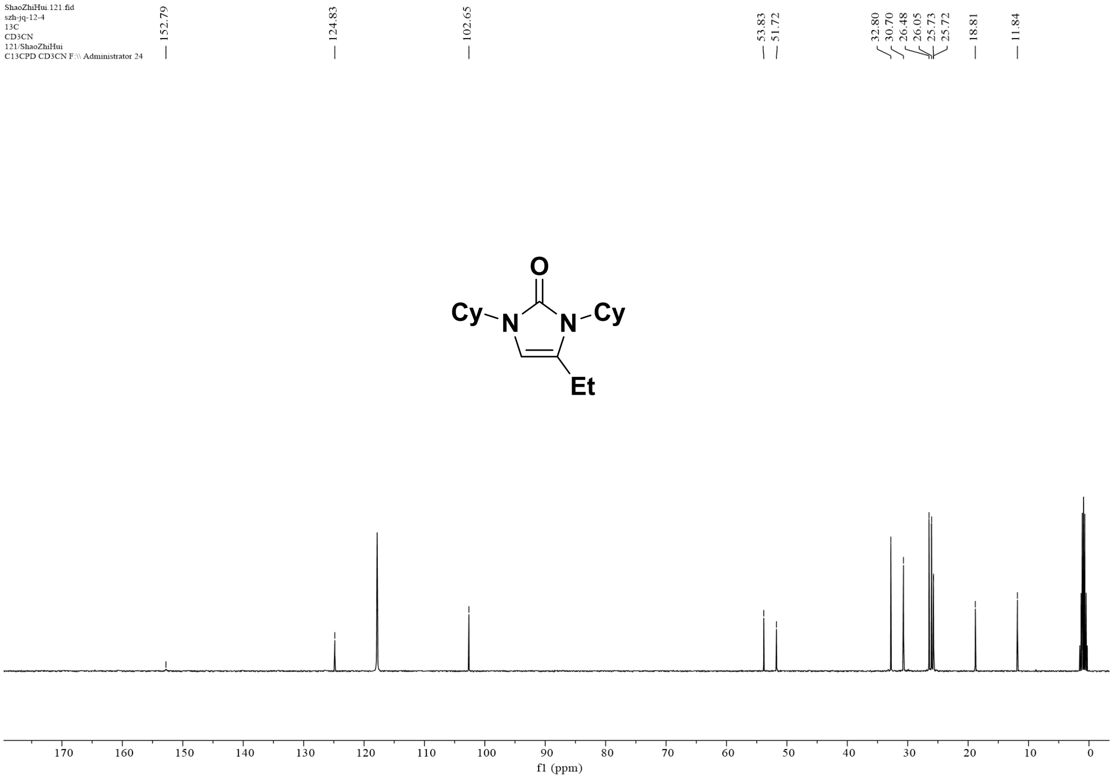

Supplementary Figure 23. ^{1}H NMR (400 MHz, CD_3CN) spectrum of 3f

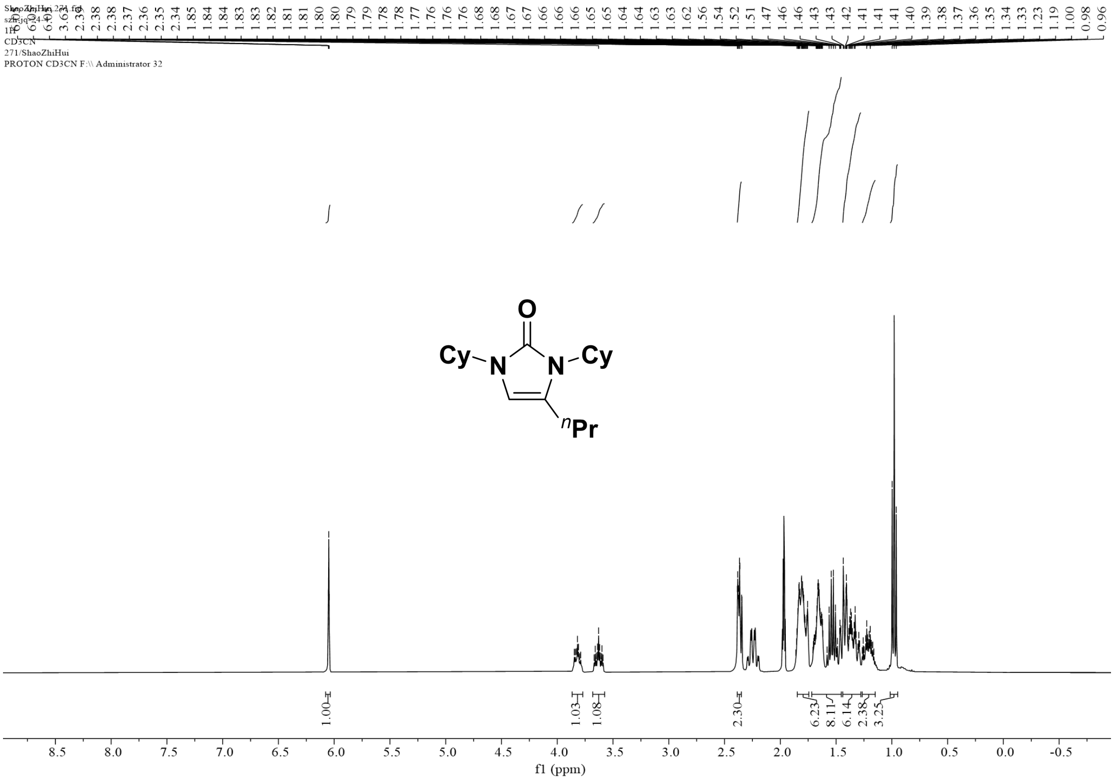

Supplementary Figure 24. ^{13}C NMR (100 MHz, CD_3CN) spectrum of 3f

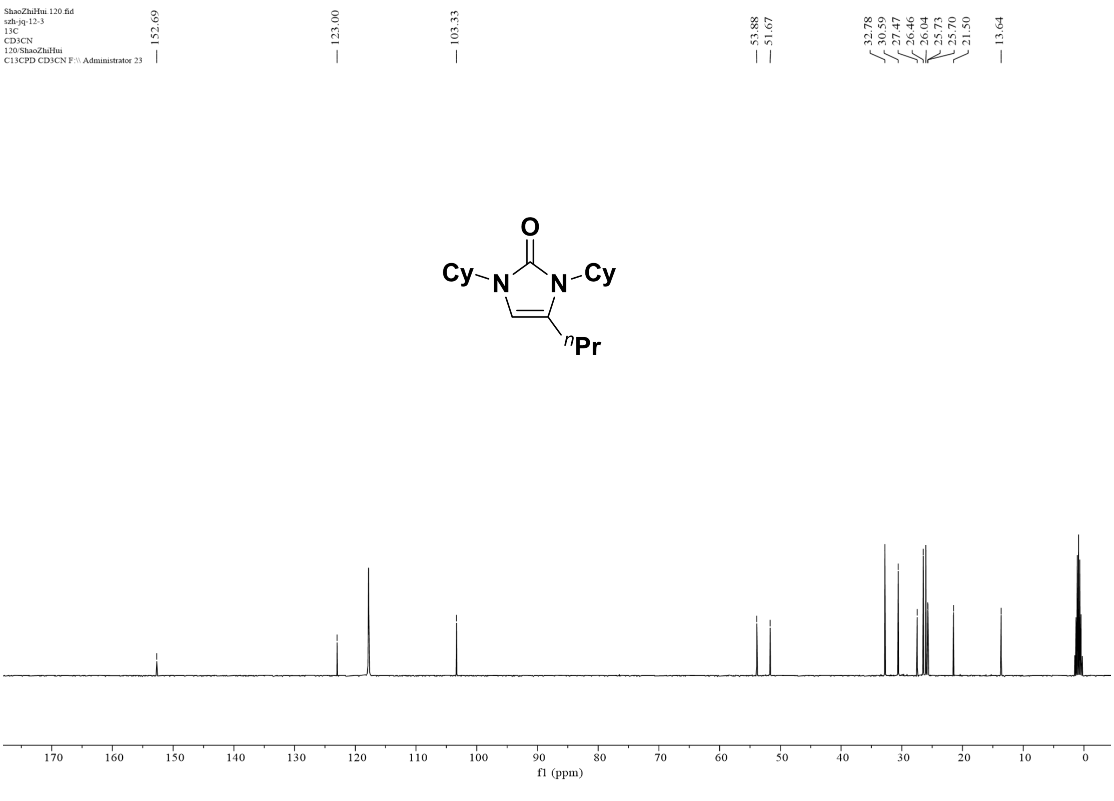

Supplementary Figure 25. ^{1}H NMR (400 MHz, CD_3CN) spectrum of 3g

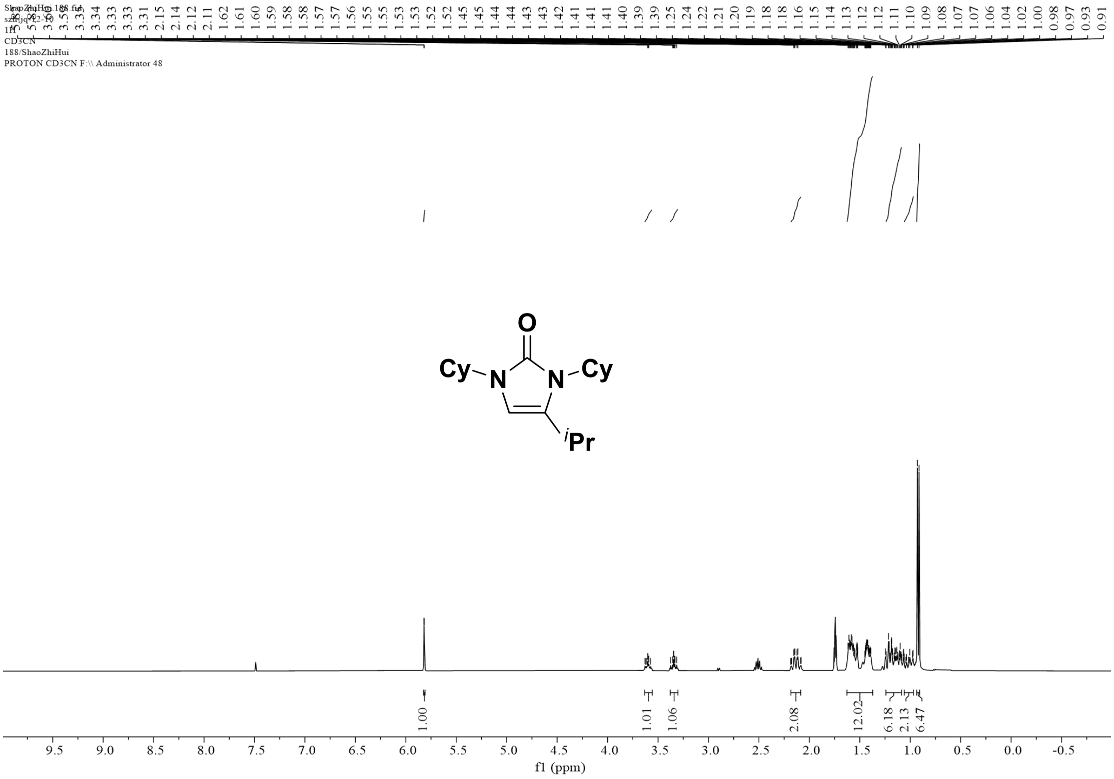

Supplementary Figure 26. ^{13}C NMR (100 MHz, CD_3CN) spectrum of 3g

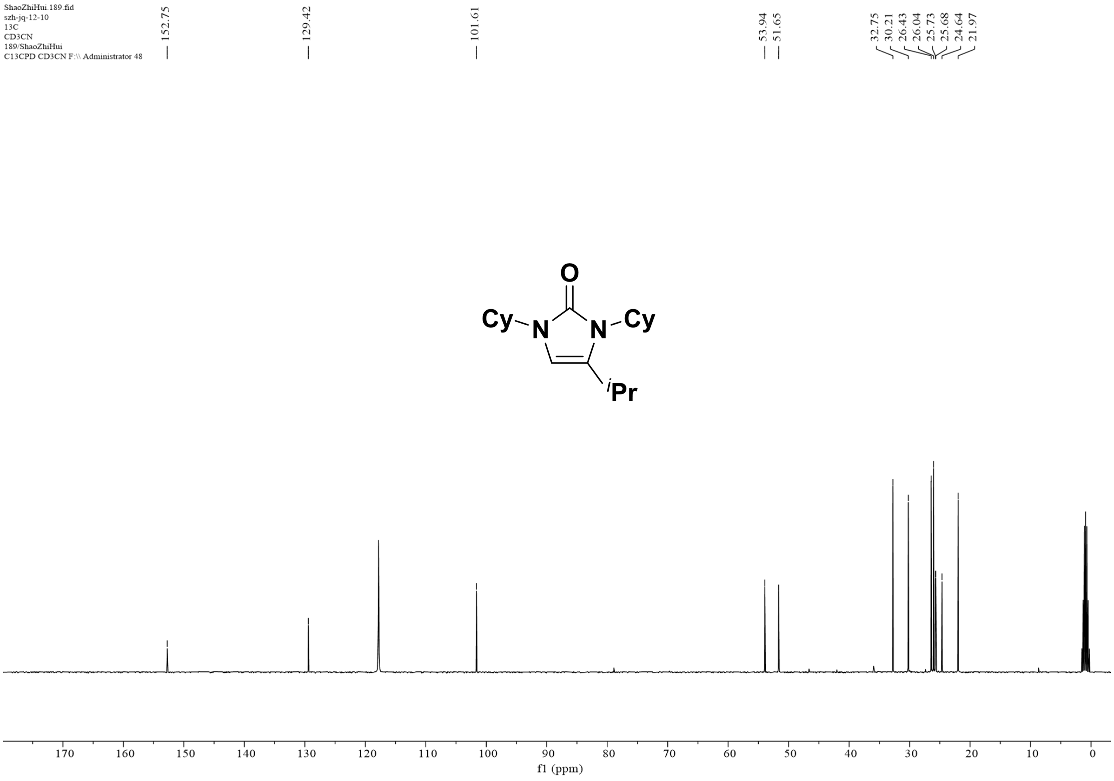

Supplementary Figure 27. ^{1}H **NMR (400 MHz,** CD_3CN**) spectrum of 3h**

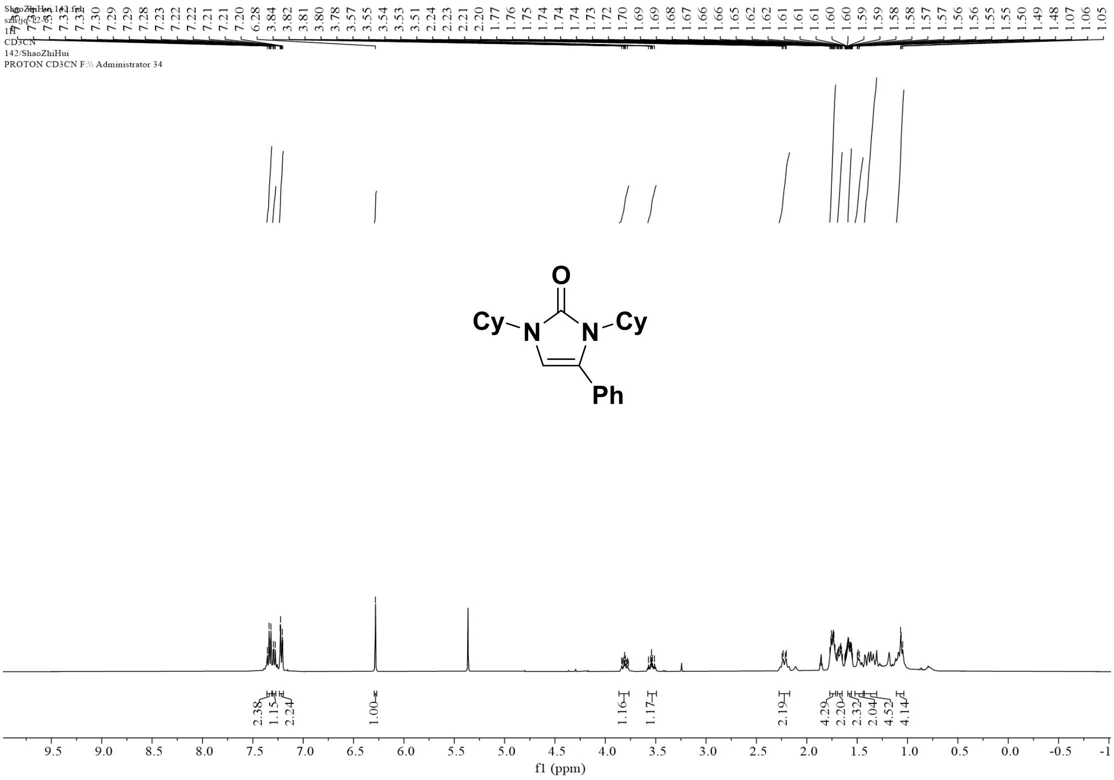

Supplementary Figure 28. ^{13}C **NMR (100 MHz,** CD_3CN**) spectrum of 3h**

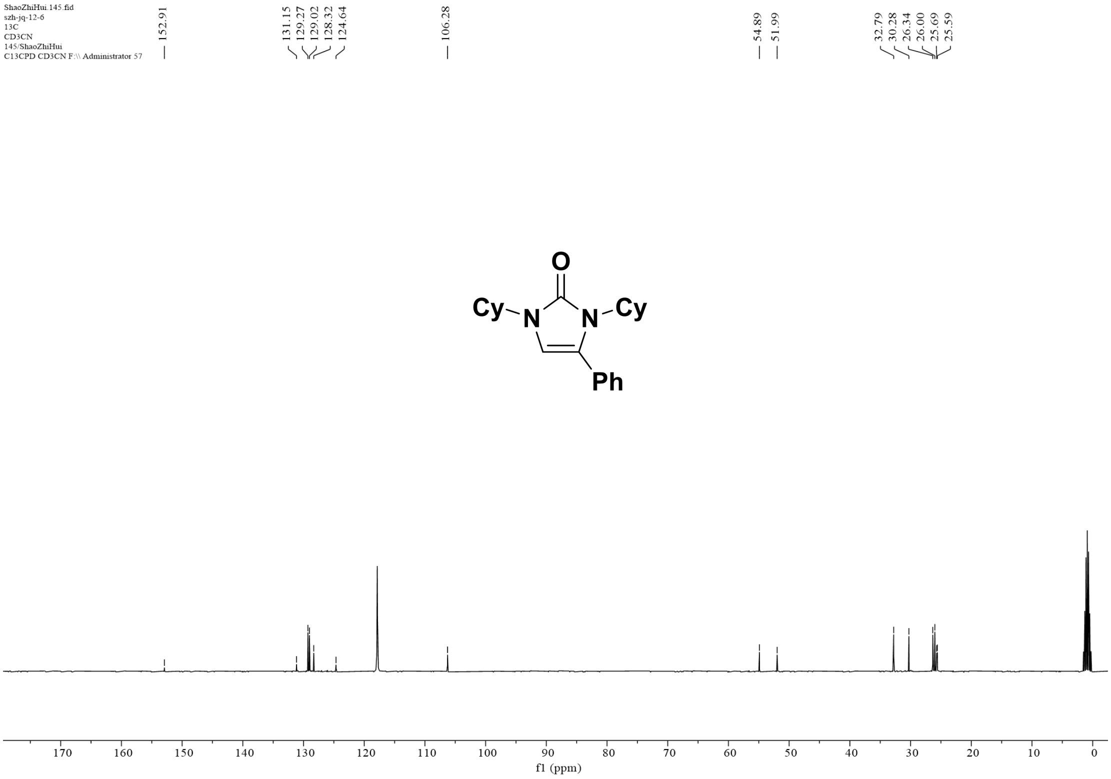

Supplementary Figure 29. ^{1}H NMR (400 MHz, CD_3CN) spectrum of 3i

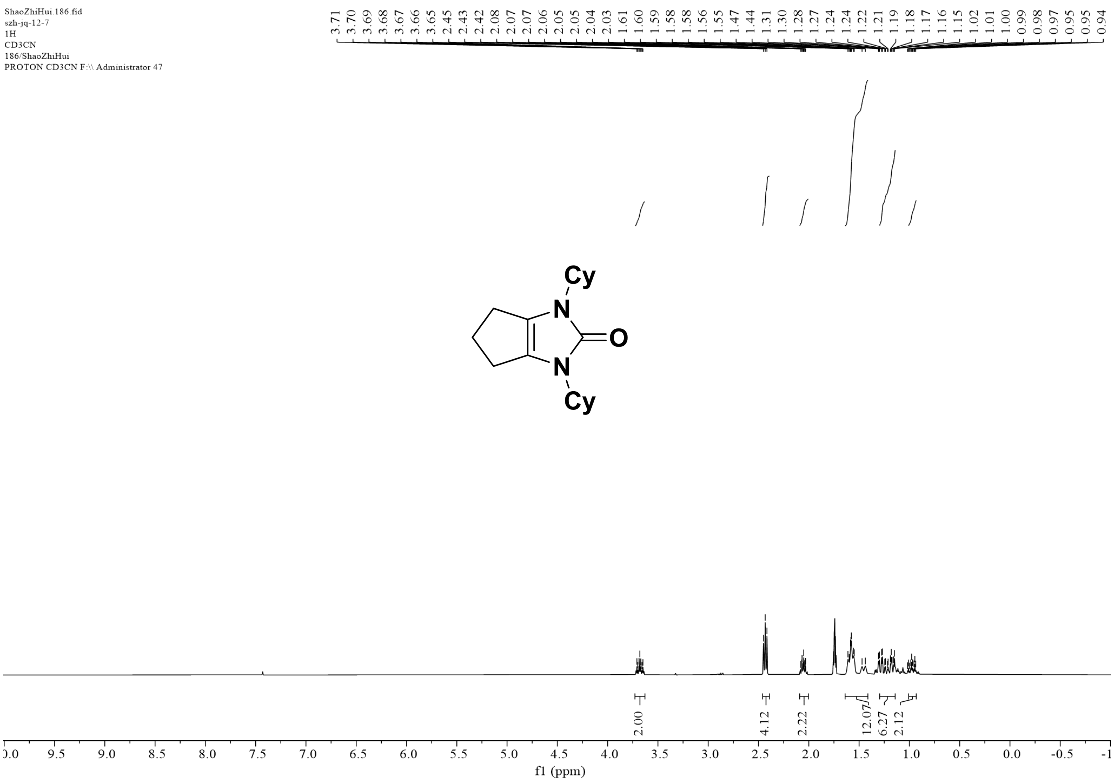

Supplementary Figure 30. ^{13}C NMR (100 MHz, CD_3CN) spectrum of 3i

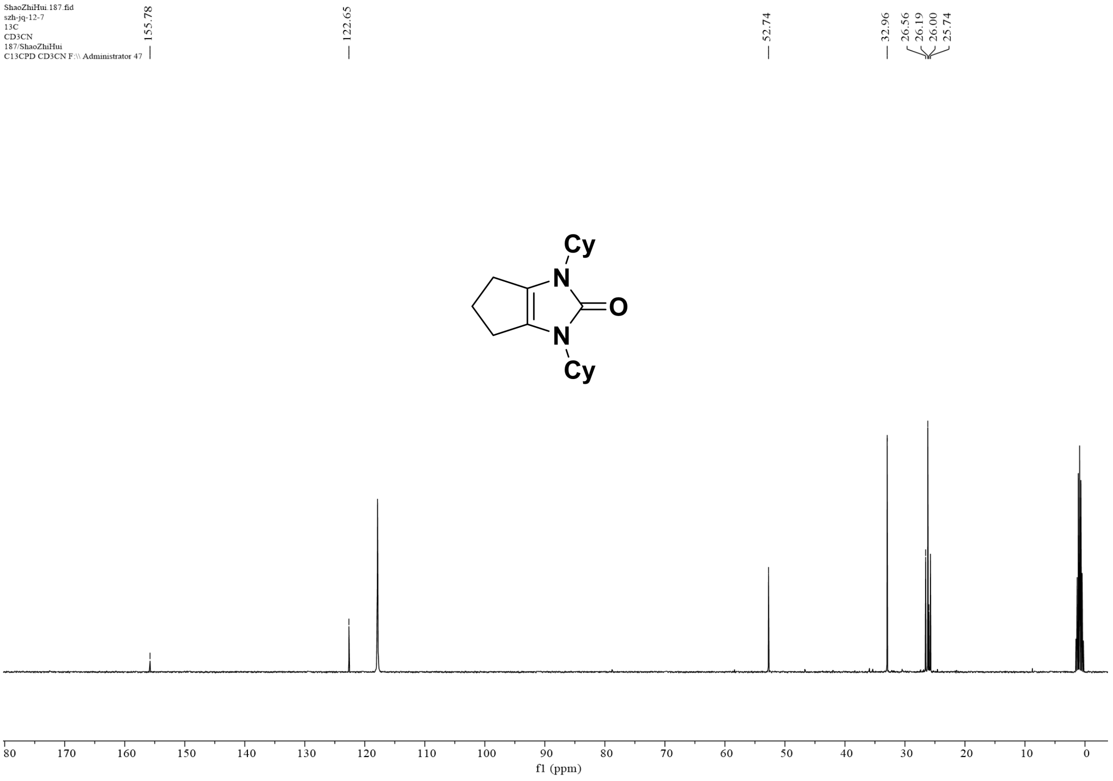

Supplementary Figure 31. ^{1}H NMR (400 MHz, CD_3CN) spectrum of 3j

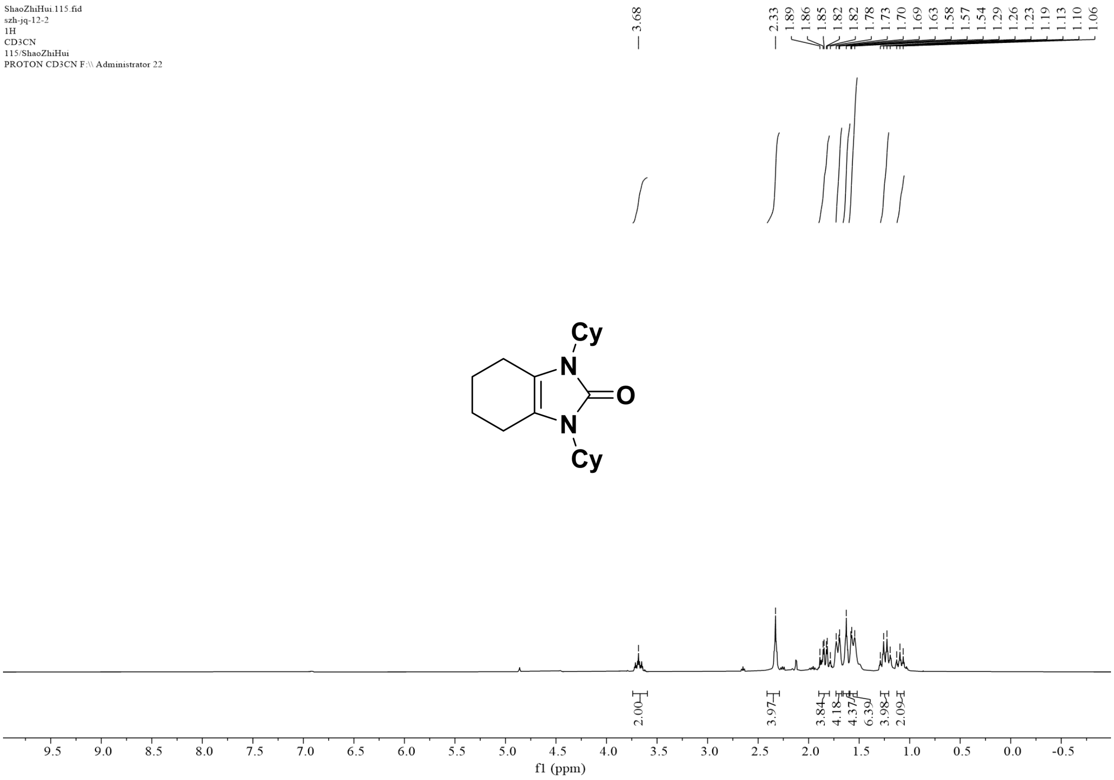

Supplementary Figure 32. ^{13}C NMR (100 MHz, CD_3CN) spectrum of 3j

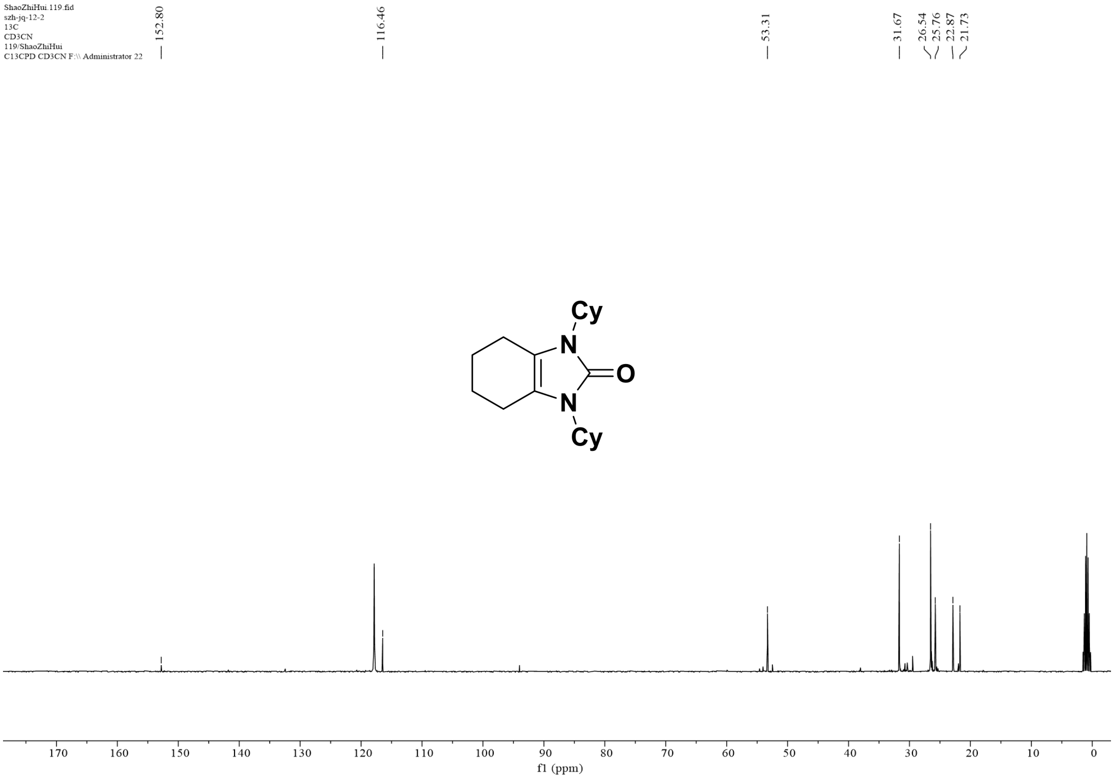

Supplementary Figure 33. ^{1}H NMR (400 MHz, CD_3CN) spectrum of 4a

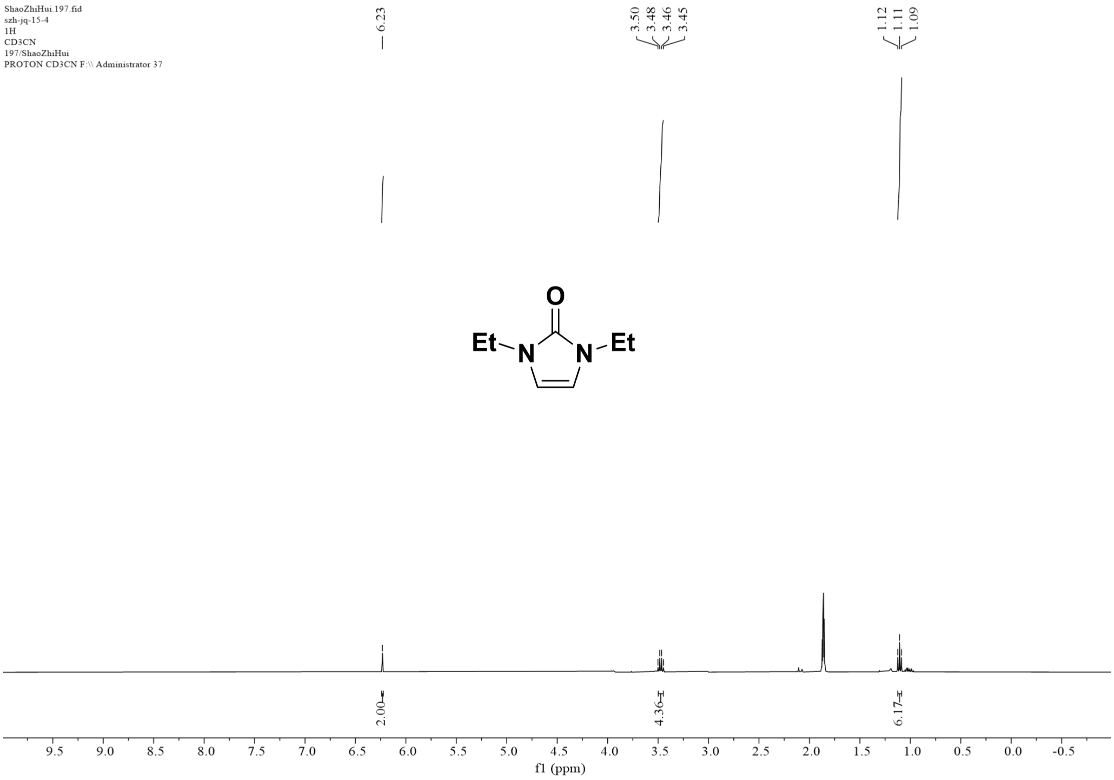

Supplementary Figure 34. ^{13}C NMR (100 MHz, CD_3CN) spectrum of 4a

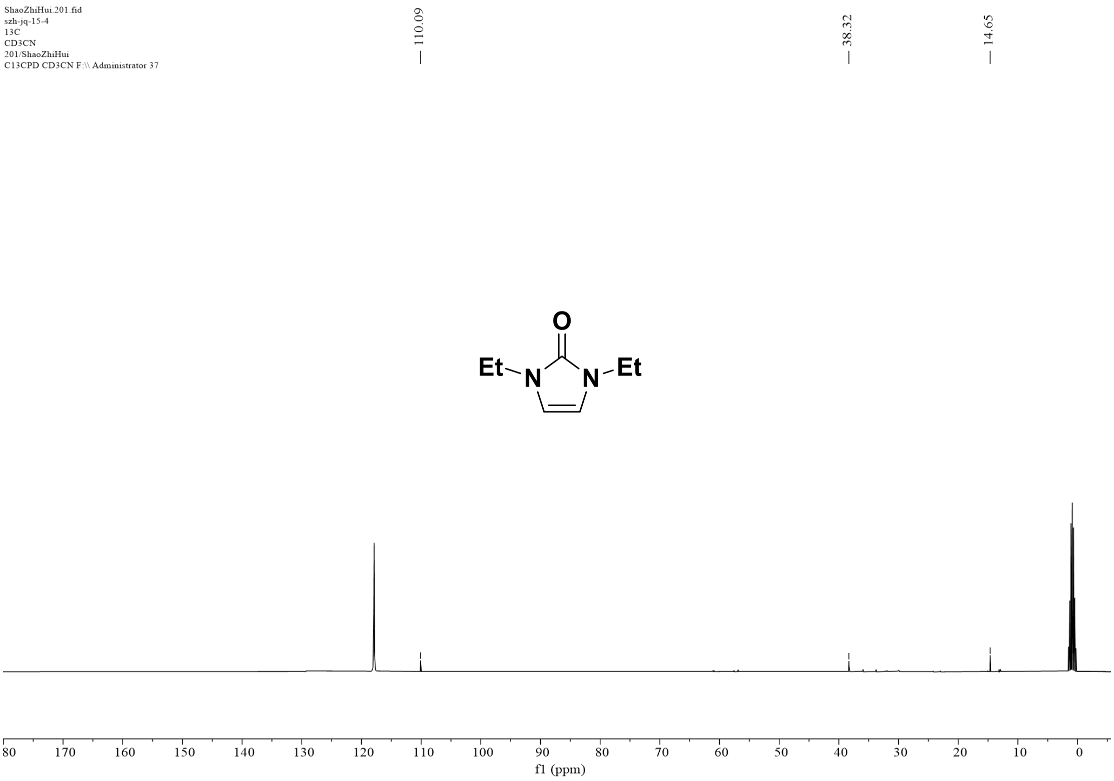

Supplementary Figure 35. ^{1}H NMR (400 MHz, CD_3CN) spectrum of 4b

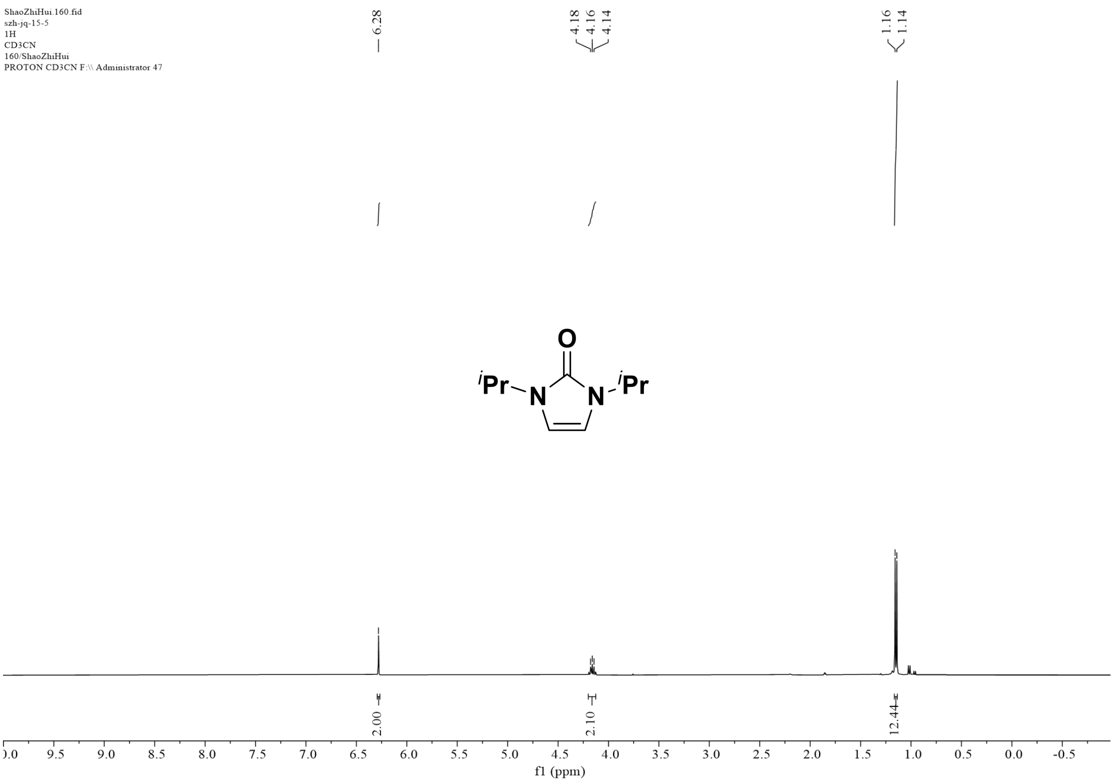

Supplementary Figure 36. ^{13}C NMR (100 MHz, CD_3CN) spectrum of 4b

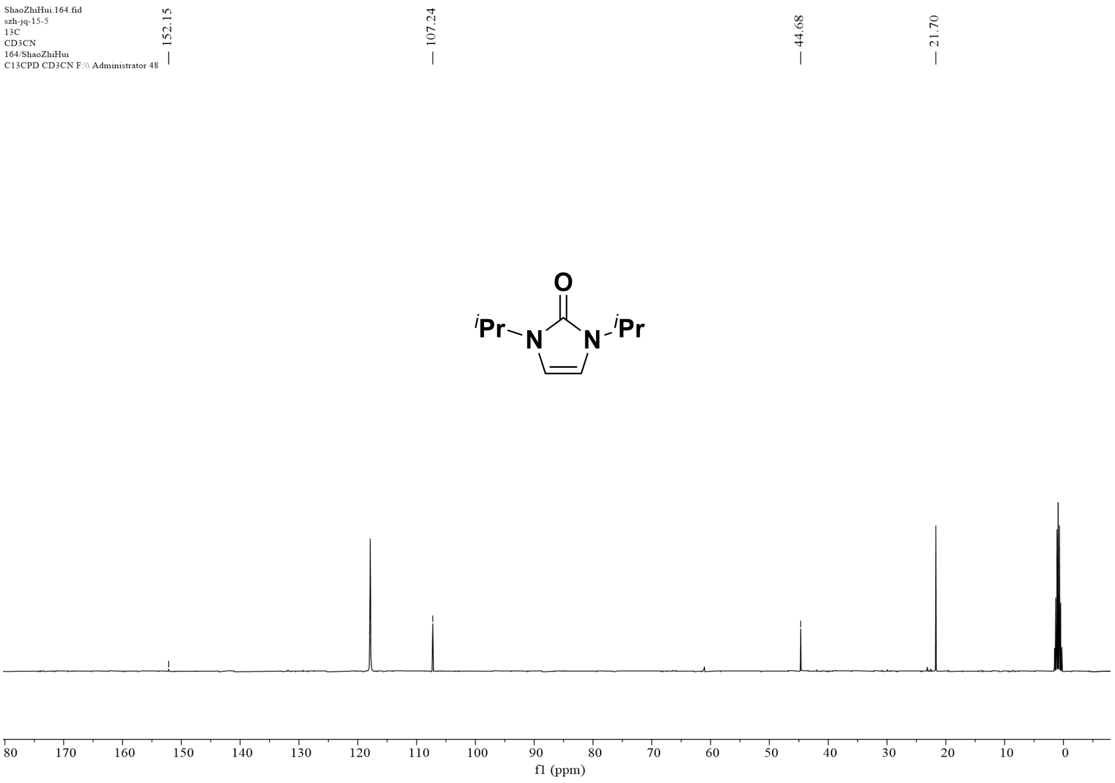

Supplementary Figure 37. ^{1}H NMR (400 MHz, CD_3CN) spectrum of 4c

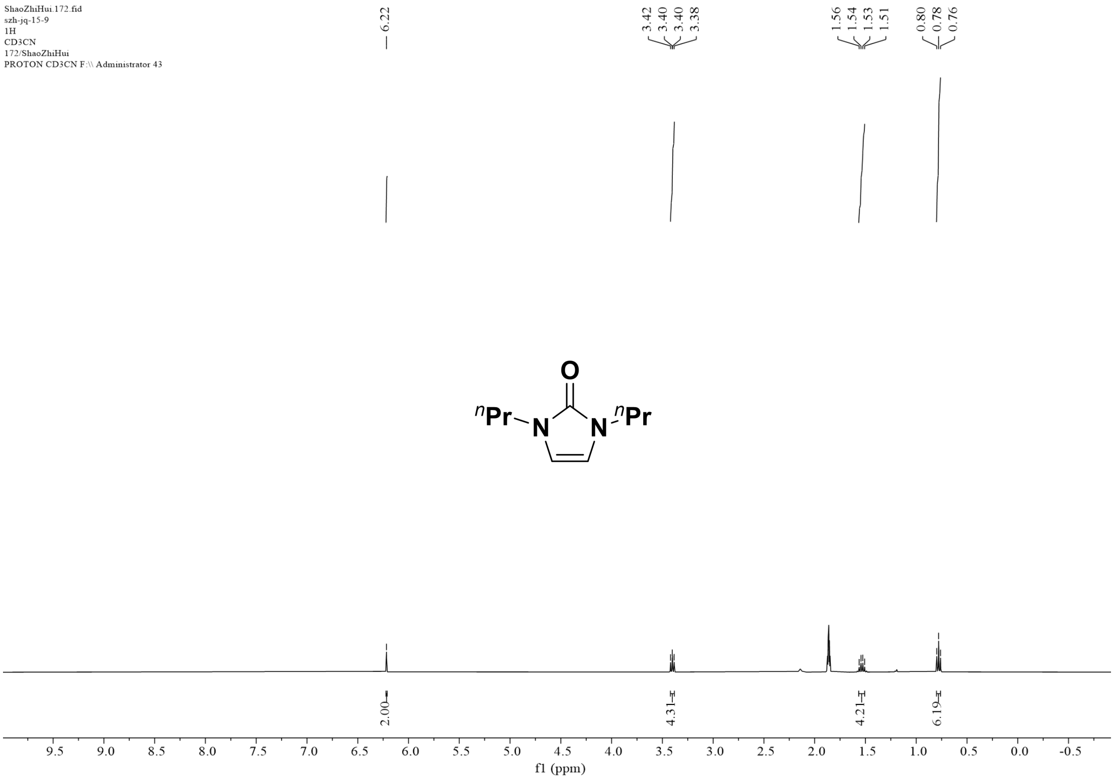

Supplementary Figure 38. ^{13}C NMR (100 MHz, CD_3CN) spectrum of 4c

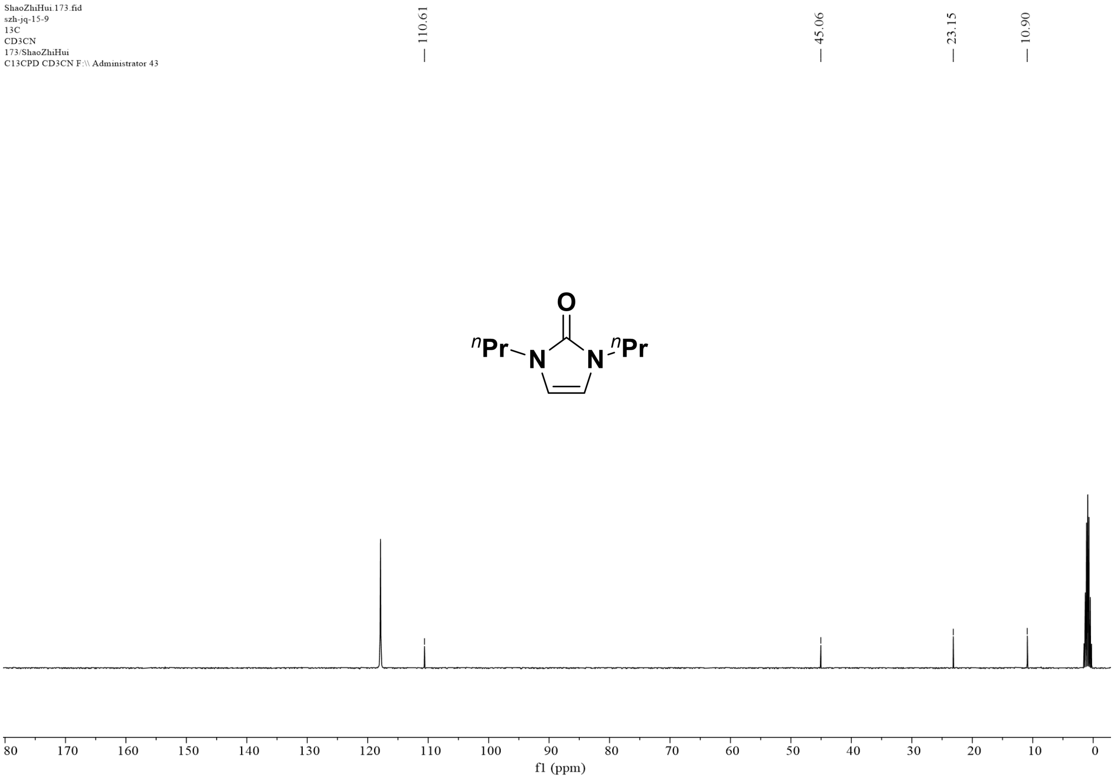

Supplementary Figure 39. ^{1}H NMR (400 MHz, CD_3CN) spectrum of 4d

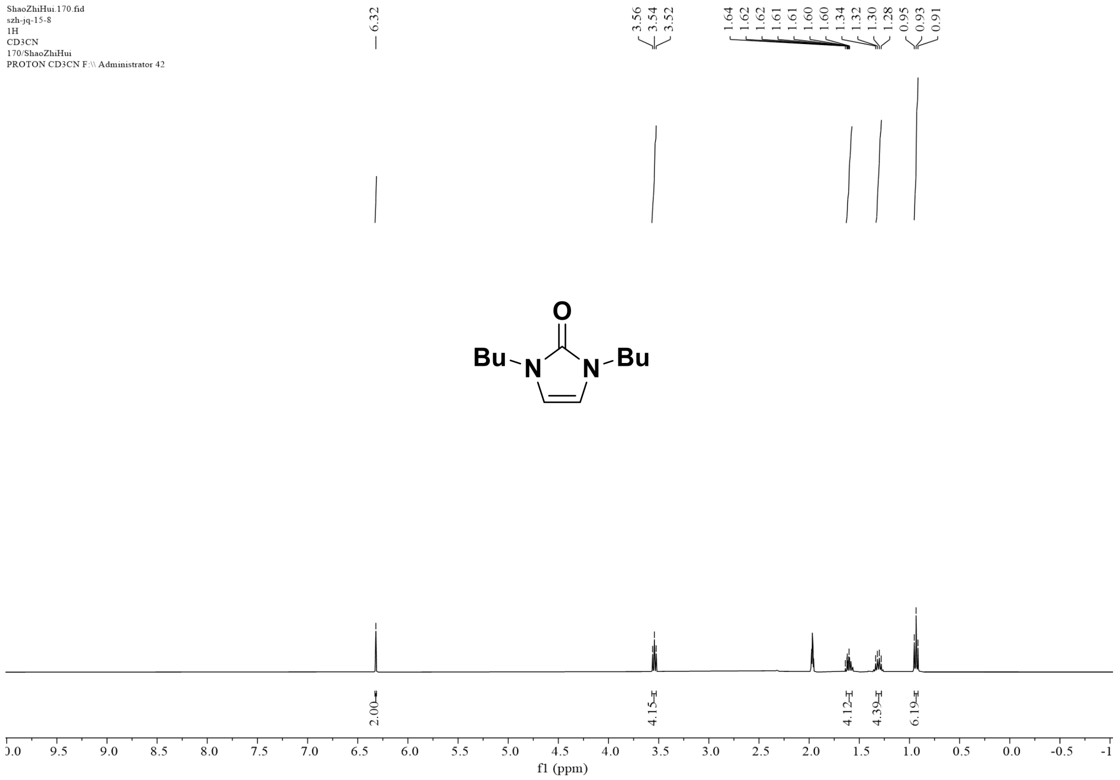

Supplementary Figure 40. ^{13}C NMR (100 MHz, CD_3CN) spectrum of 4d

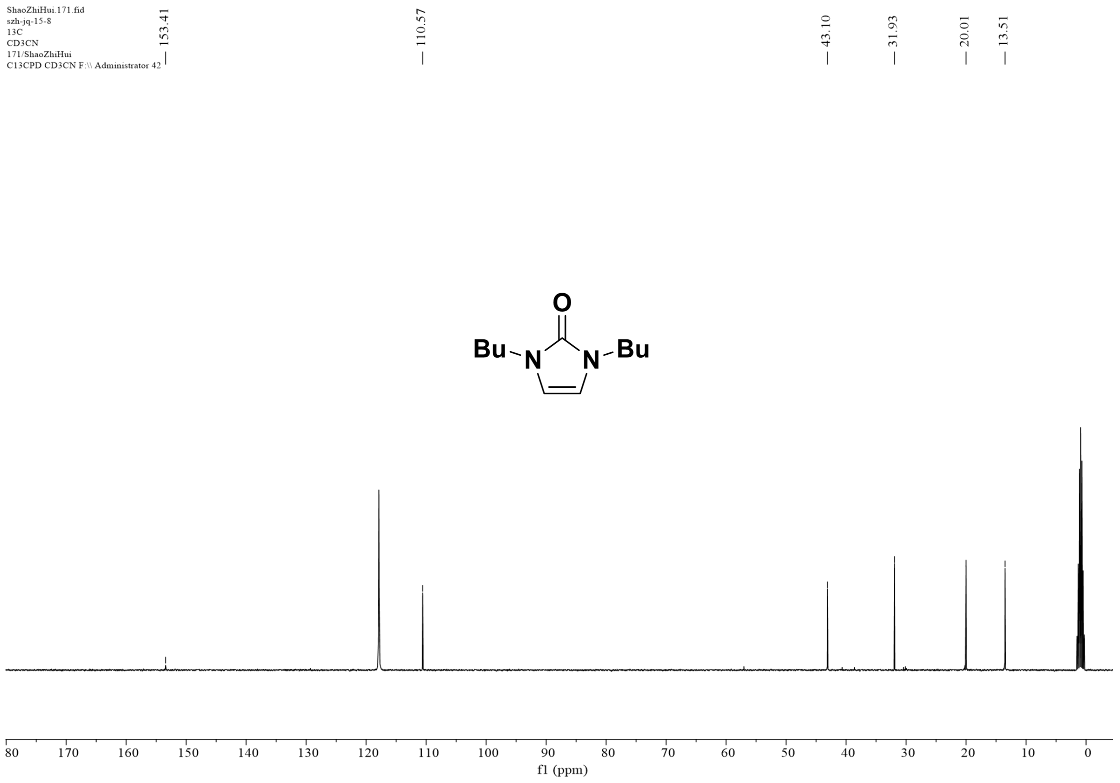

Supplementary Figure 41. ^{1}H NMR (400 MHz, CD_3CN) spectrum of 4e

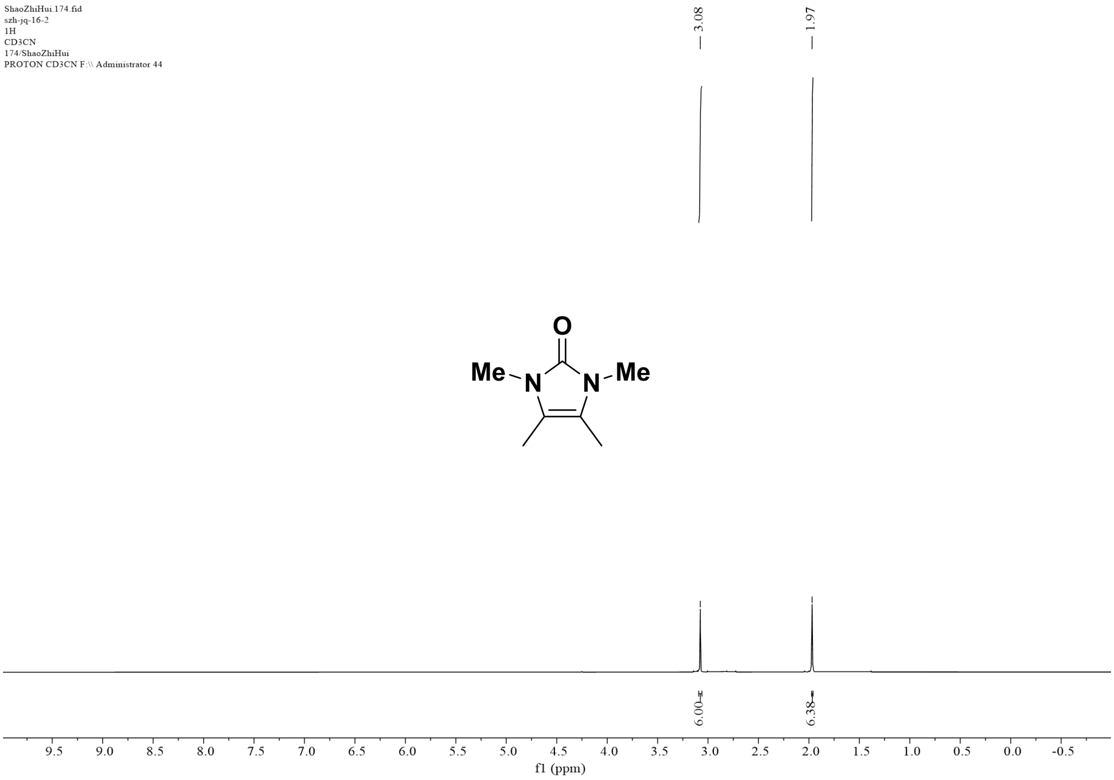

Supplementary Figure 42. ^{13}C NMR (100 MHz, CD_3CN) spectrum of 4e

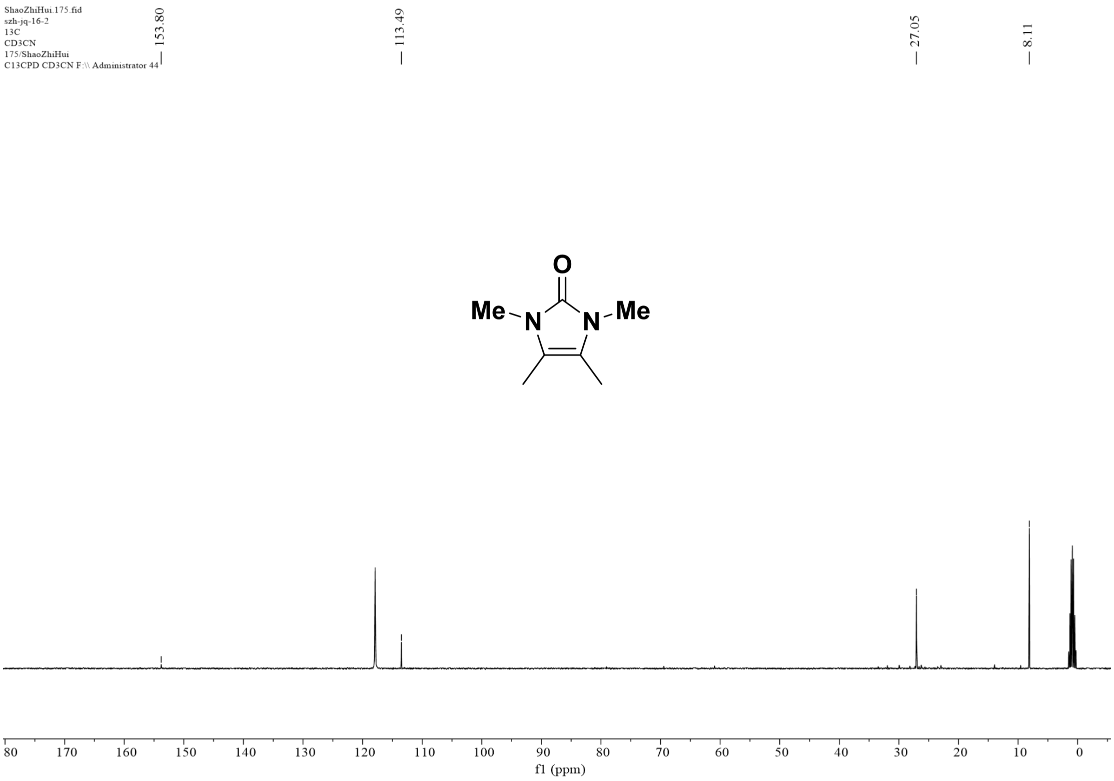

Supplementary Figure 43. ^{1}H NMR (400 MHz, CD_3CN) spectrum of 4f

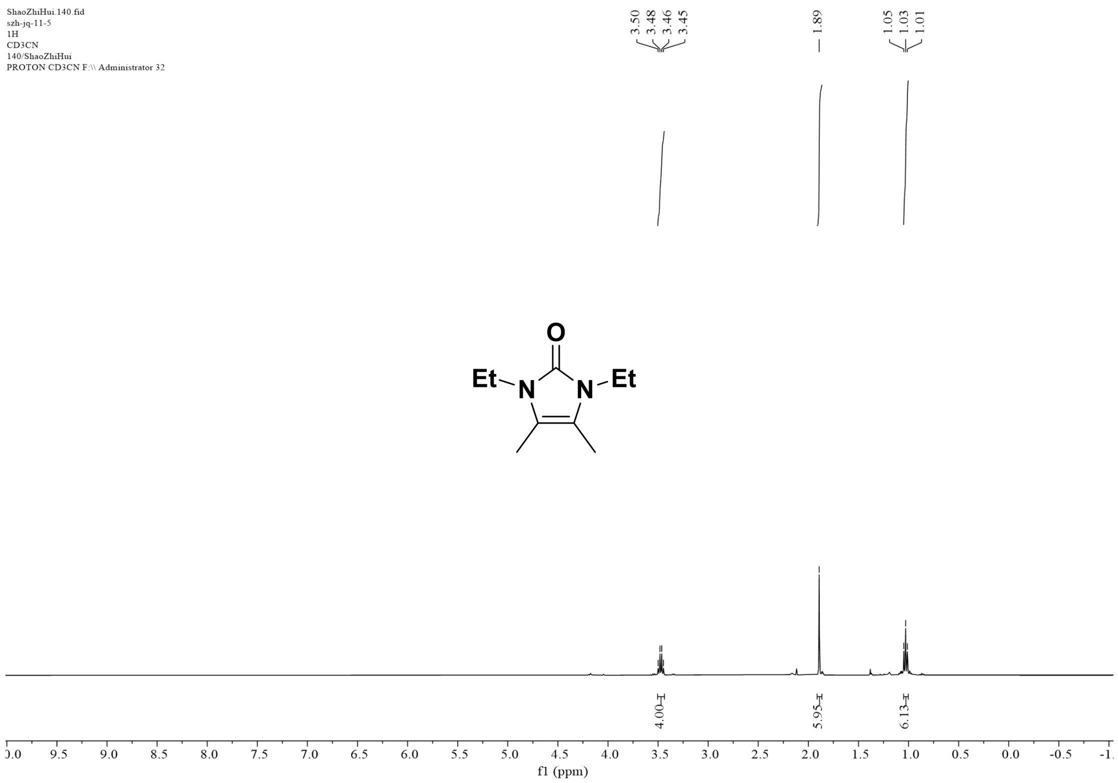

Supplementary Figure 44. ^{13}C NMR (100 MHz, CD_3CN) spectrum of 4f

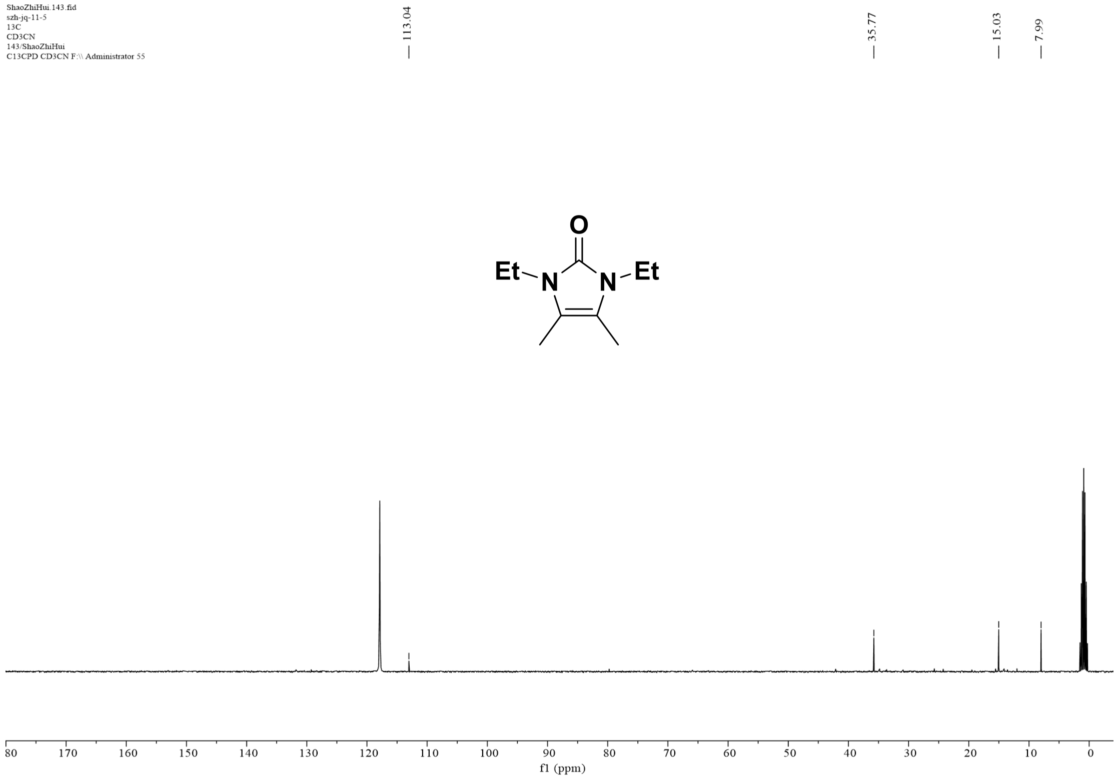

Supplementary Figure 45. ^{1}H NMR (400 MHz, CD_3CN) spectrum of 4g

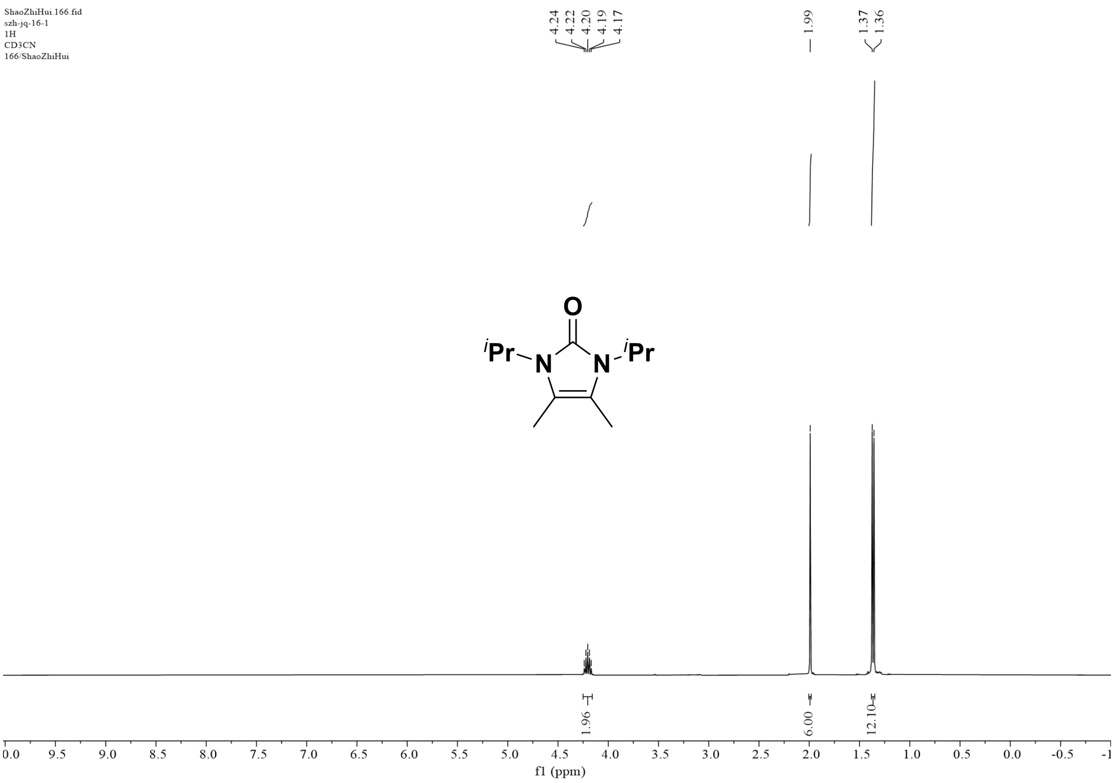

Supplementary Figure 46. ^{13}C NMR (100 MHz, CD_3CN) spectrum of 4g

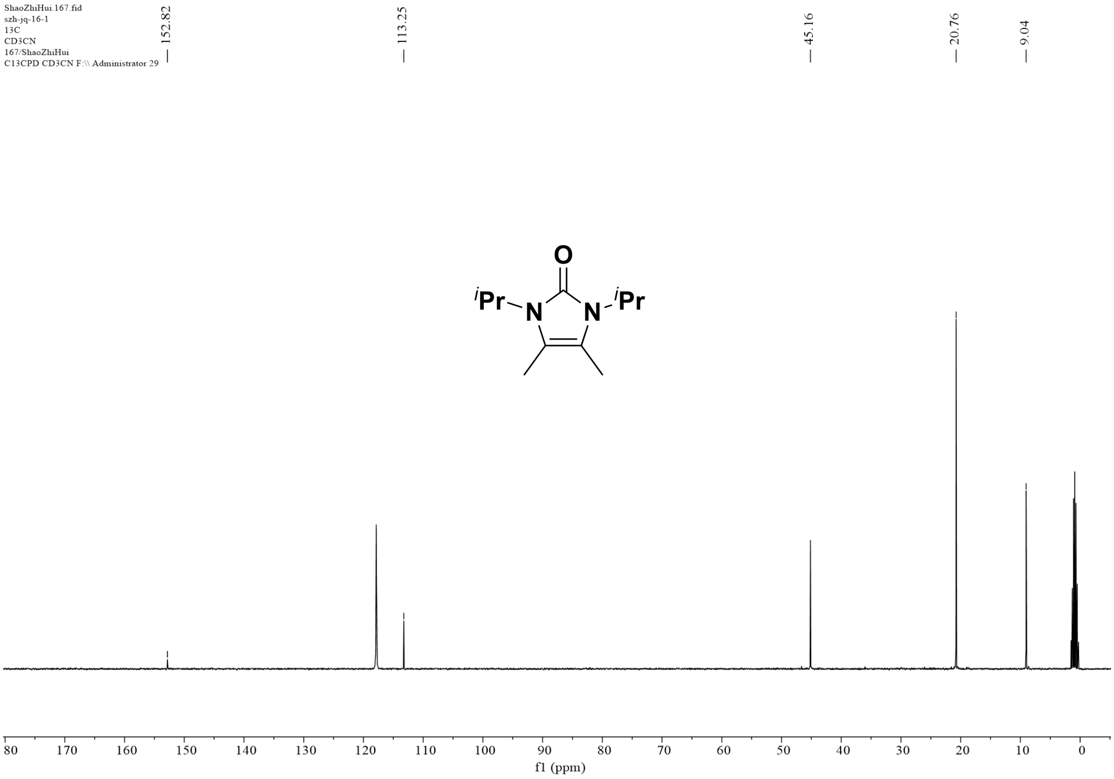

Supplementary Figure 47. ^{1}H NMR (400 MHz, CD_3CN) spectrum of 4h

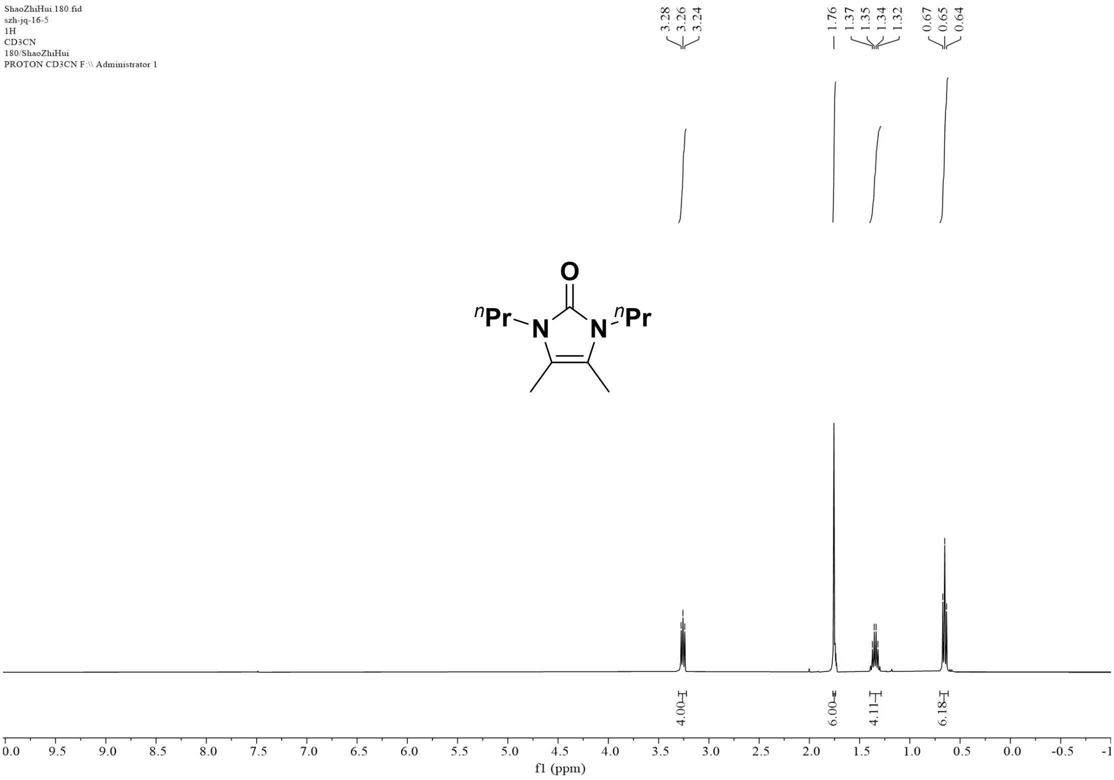

Supplementary Figure 48. ^{13}C NMR (100 MHz, CD_3CN) spectrum of 4h

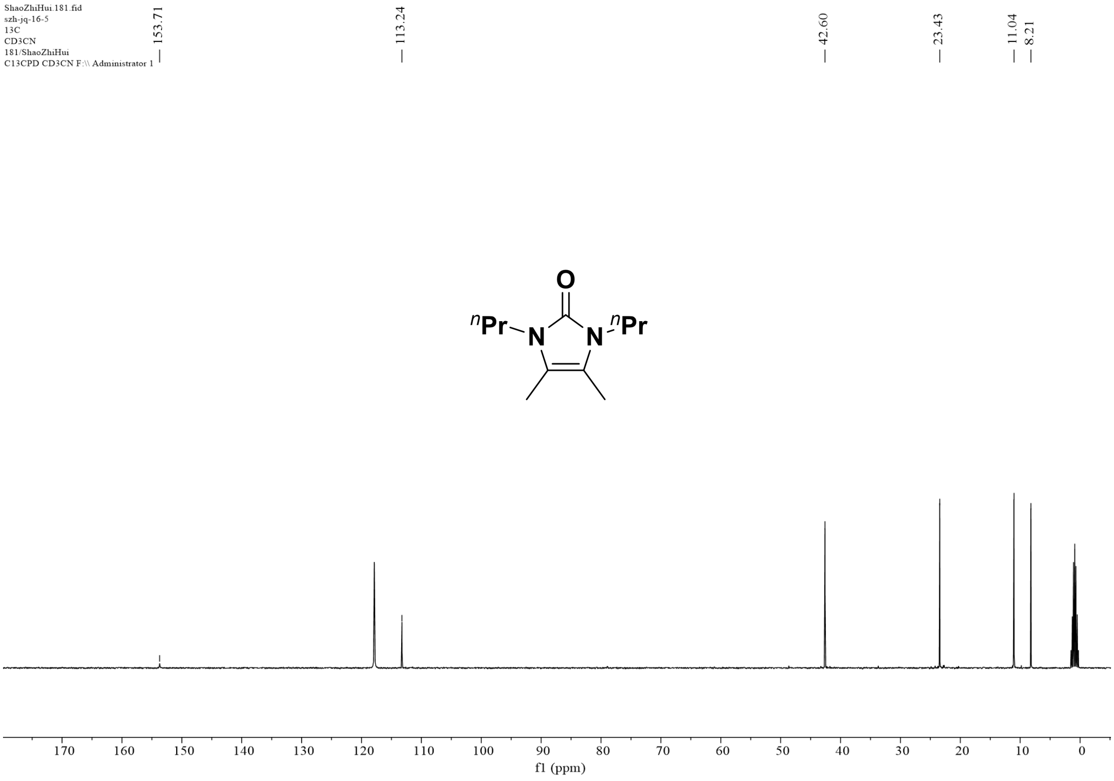

Supplementary Figure 49. ^{1}H NMR (400 MHz, CD_3CN) spectrum of 4i

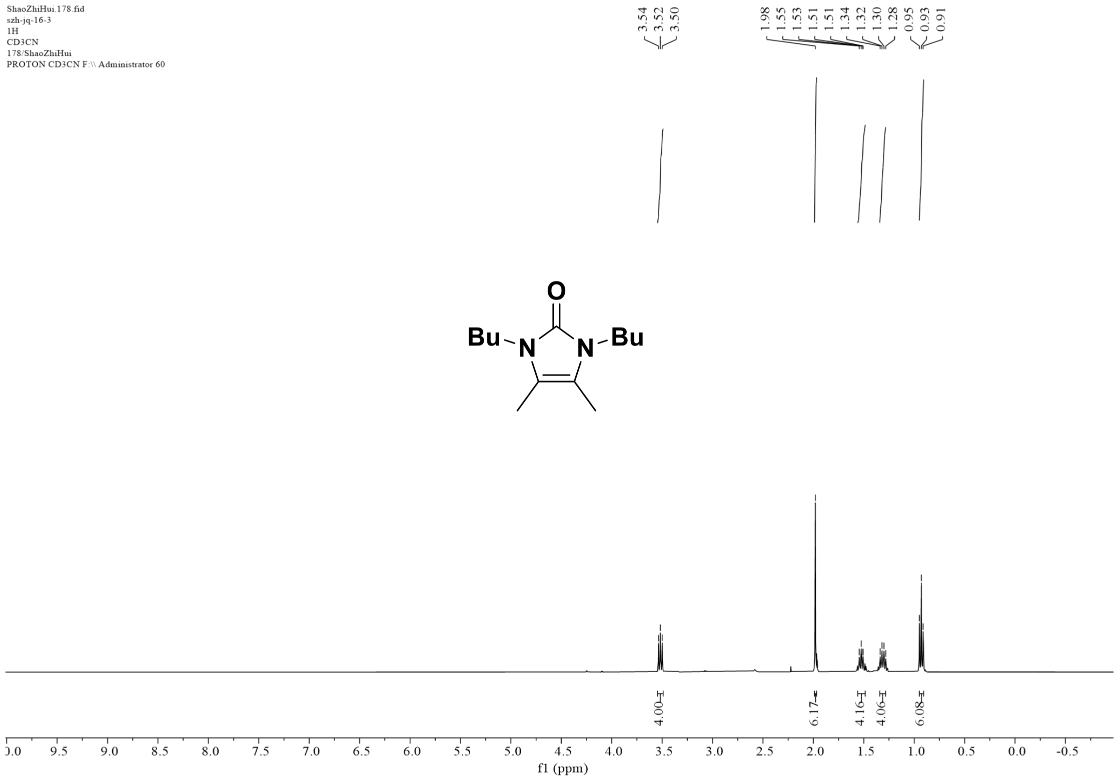

Supplementary Figure 50. ^{13}C NMR (100 MHz, CD_3CN) spectrum of 4i

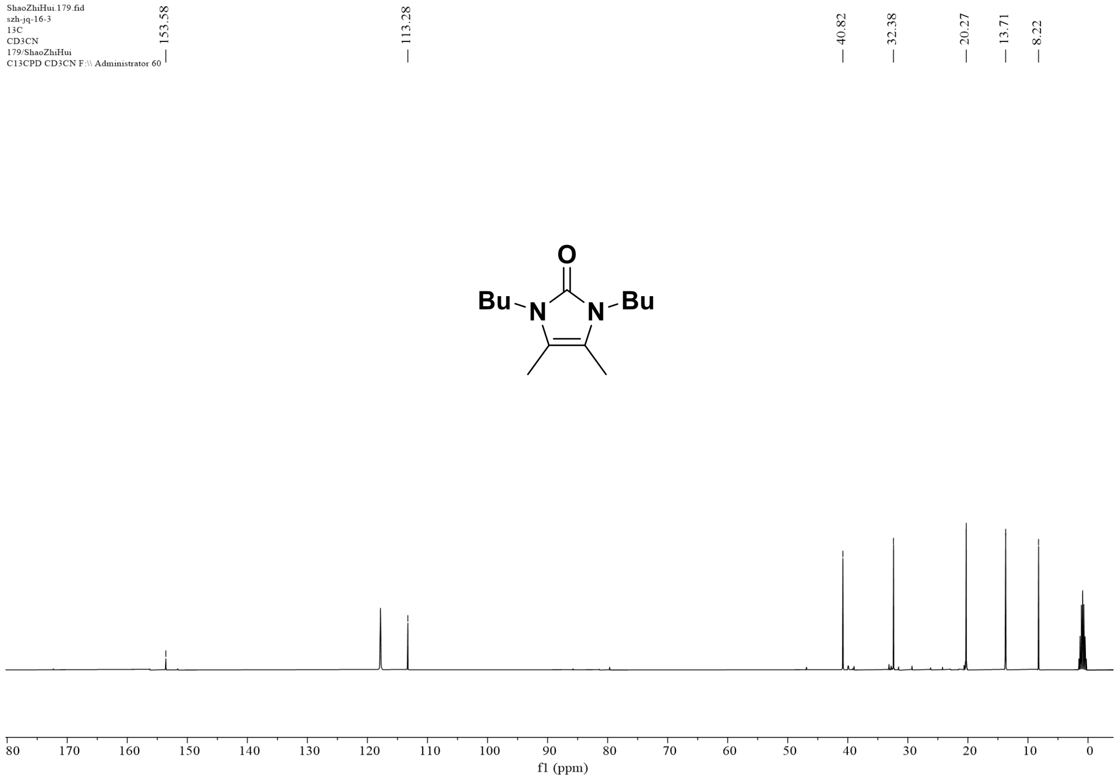

Supplementary Figure 51. ^{1}H NMR (400 MHz, CD_3CN) spectrum of 4j

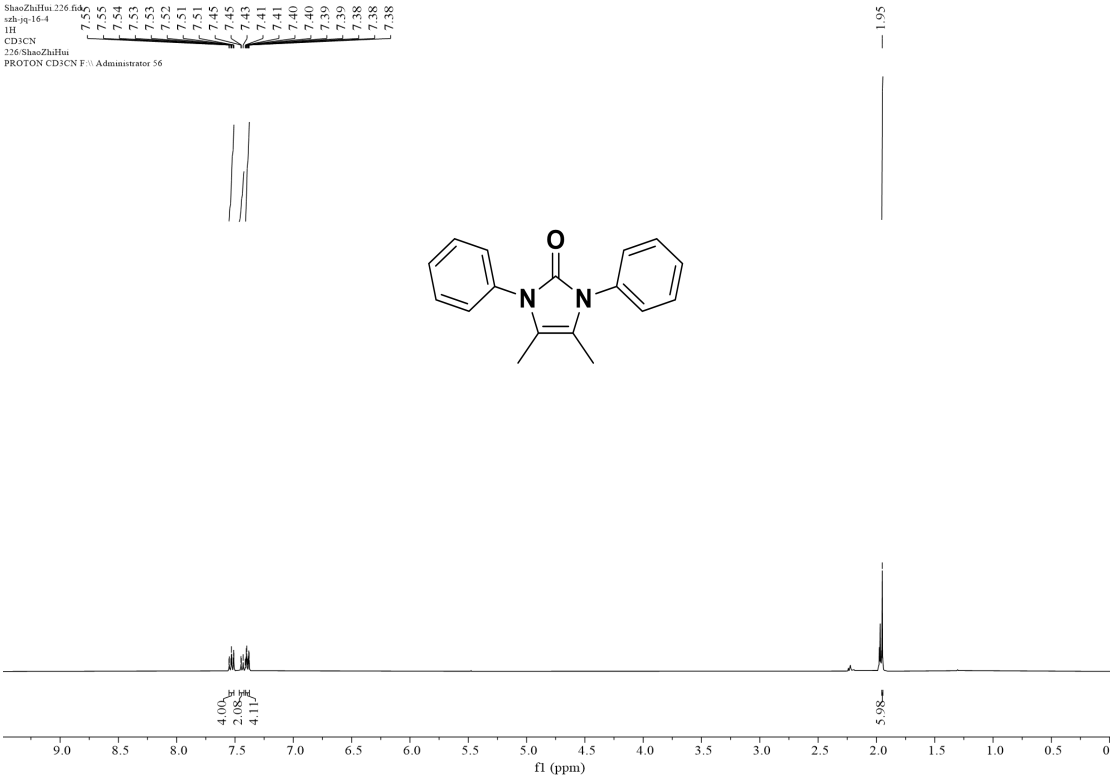

Supplementary Figure 52. ^{13}C NMR (100 MHz, CD_3CN) spectrum of 4j

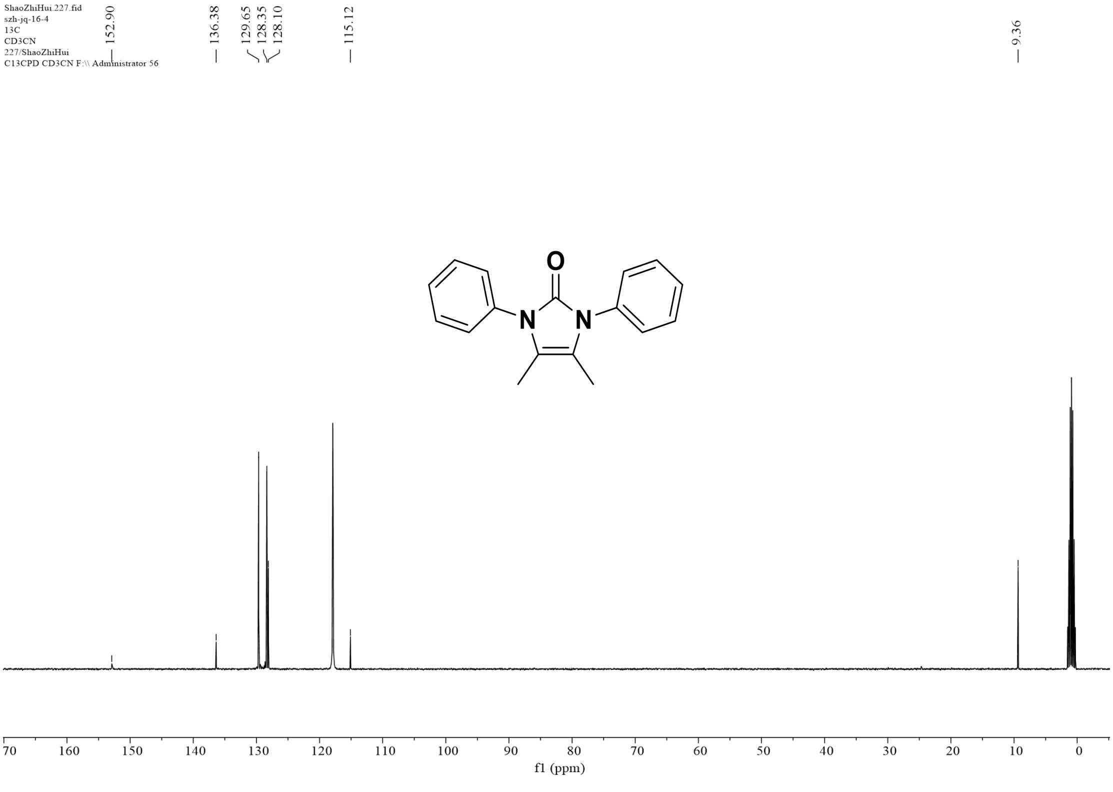

Supplementary Figure 53. ^{1}H NMR (400 MHz, MeOD) spectrum of 4k

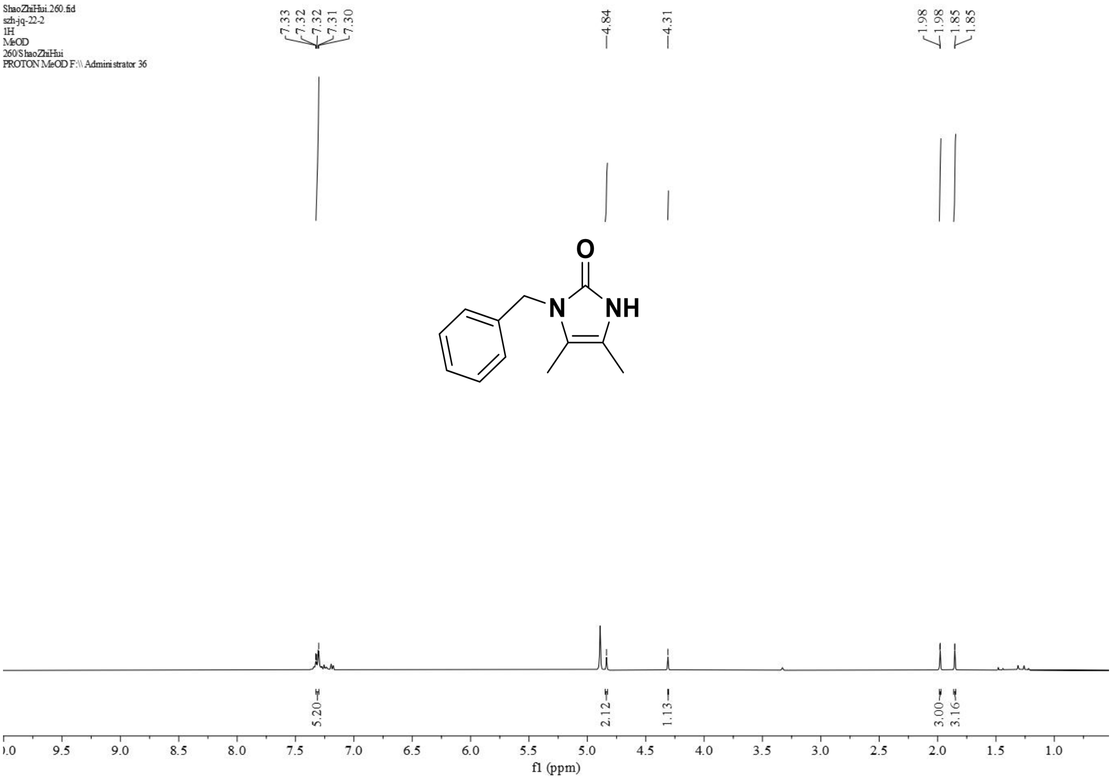

Supplementary Figure 54. ^{13}C NMR (100 MHz, MeOD) spectrum of 4k

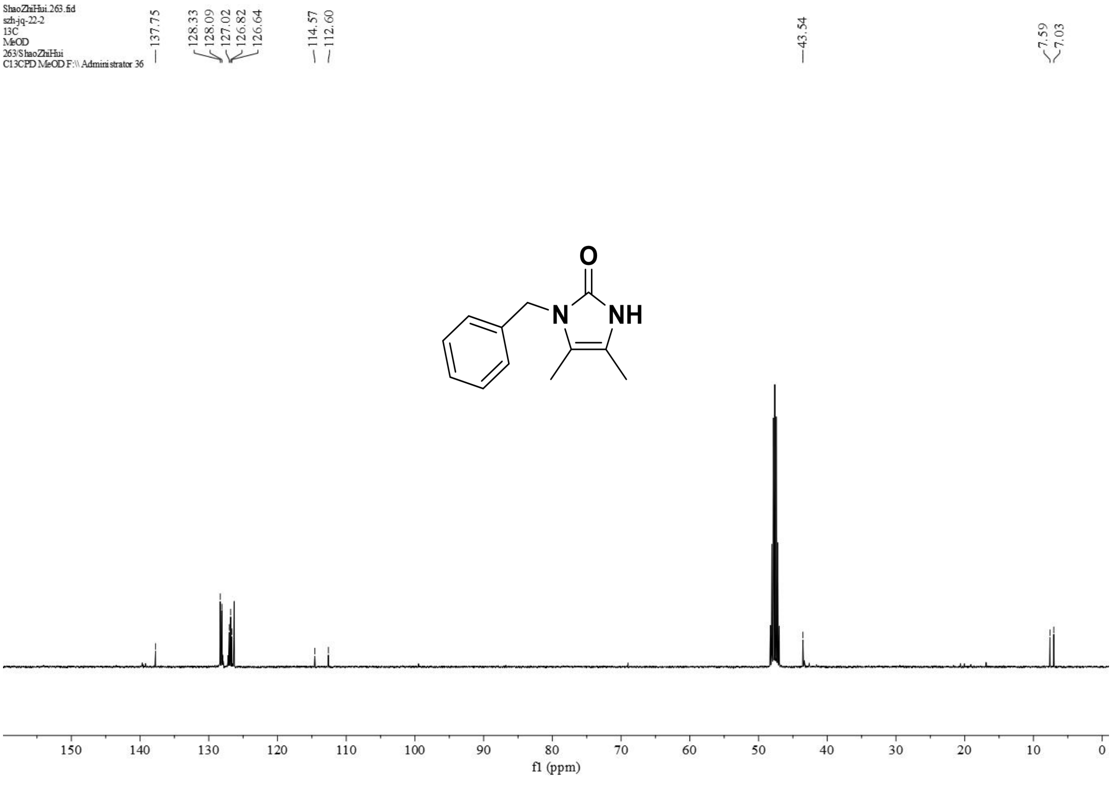

Supplementary Figure 55. ^{1}H NMR (400 MHz, CD_3CN) spectrum of 4l

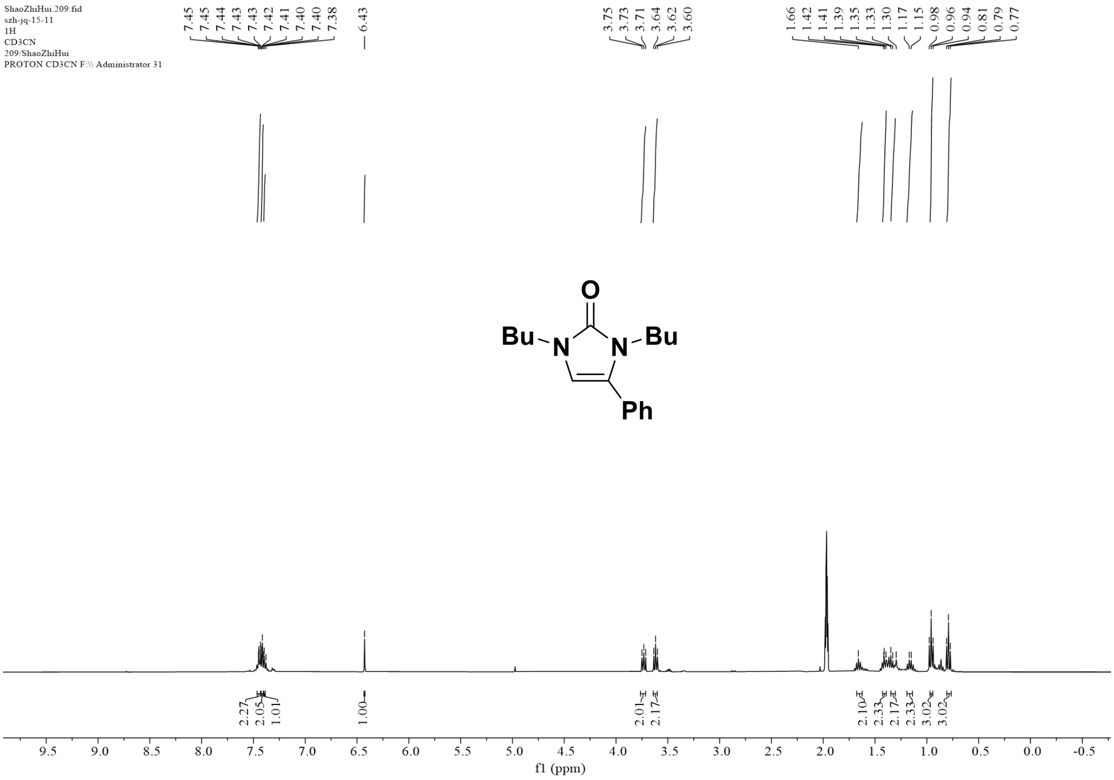

Supplementary Figure 56. ^{13}C NMR (100 MHz, CD_3CN) spectrum of 4l

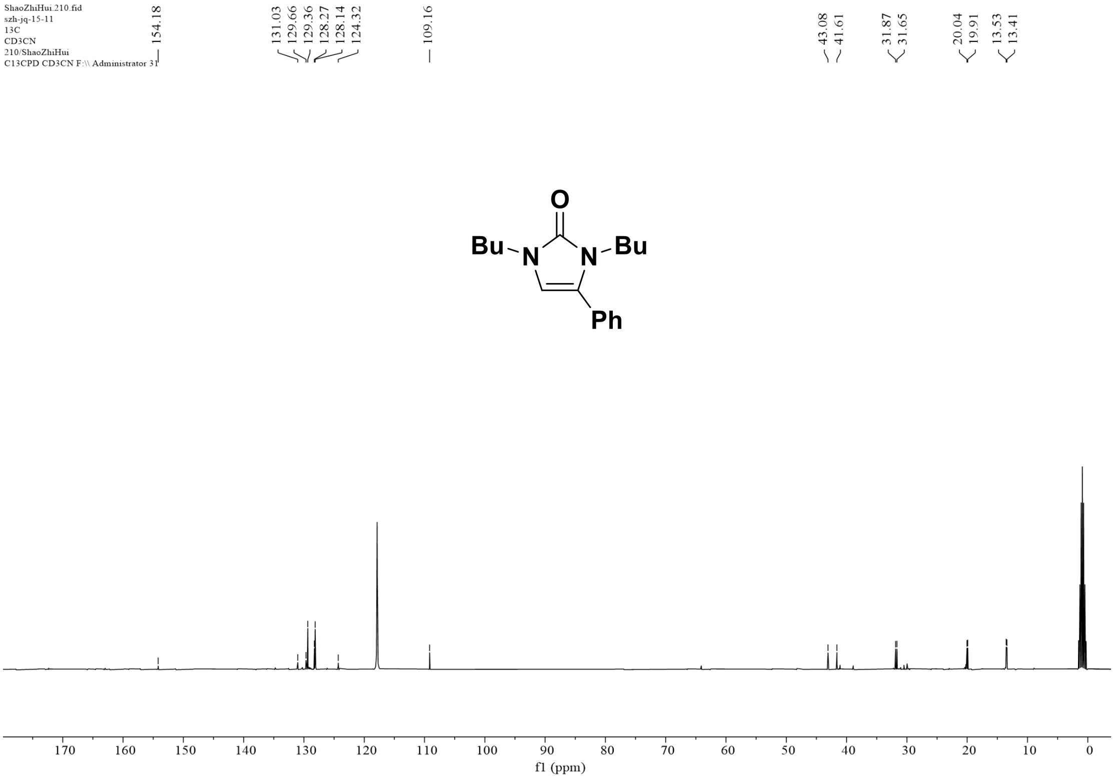

Supplementary Figure 57. ^{1}H NMR (400 MHz, CD_3CN) spectrum of 4m

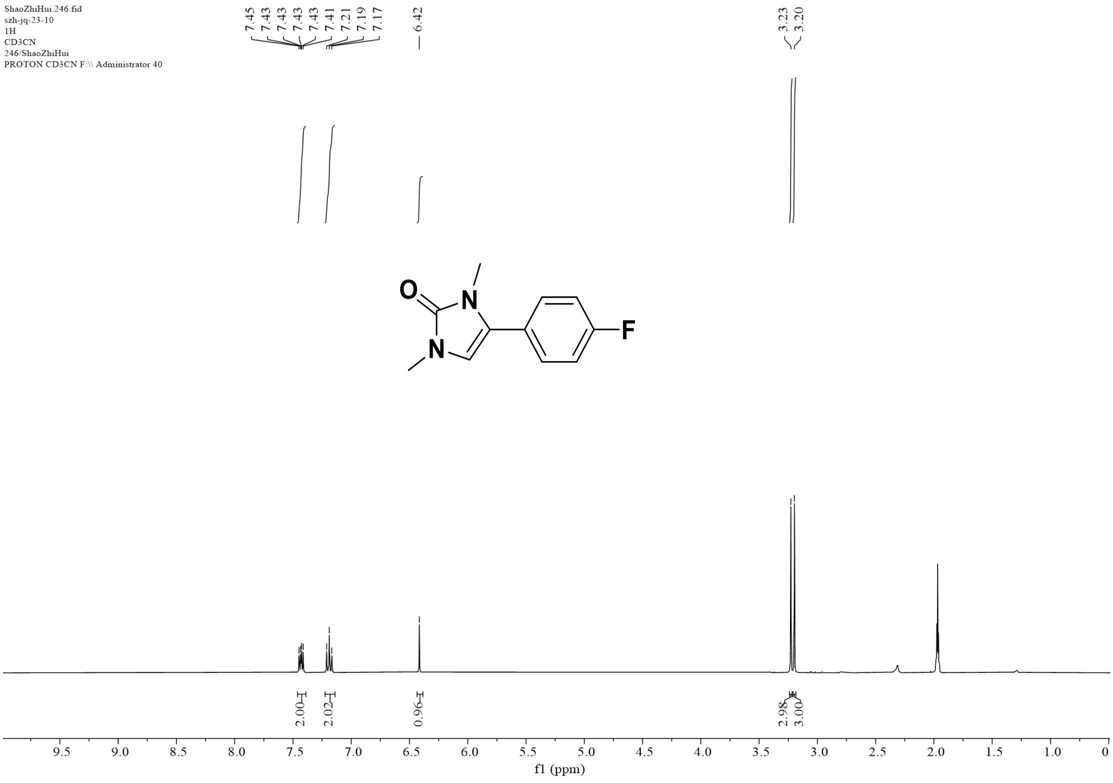

Supplementary Figure 58. ^{13}C NMR (100 MHz, CD_3CN) spectrum of 4m

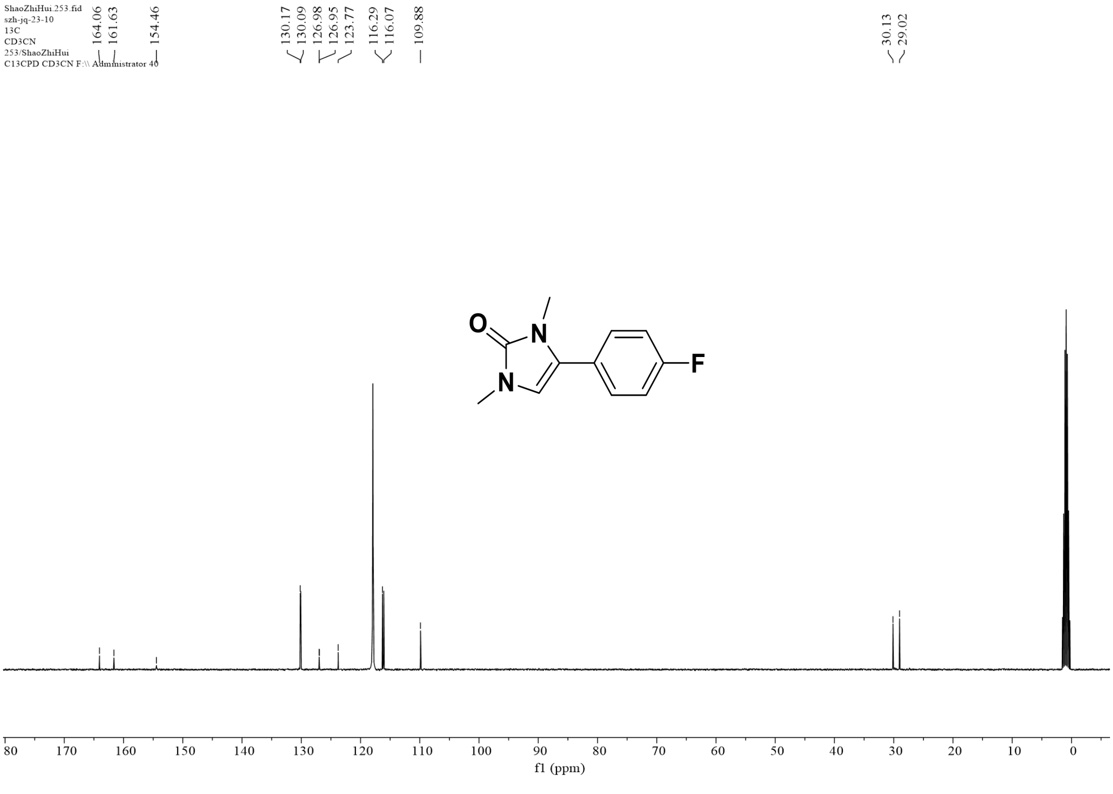

Supplementary Figure 59. ^{19}F NMR (376 MHz, CD_3CN) spectrum of 4m

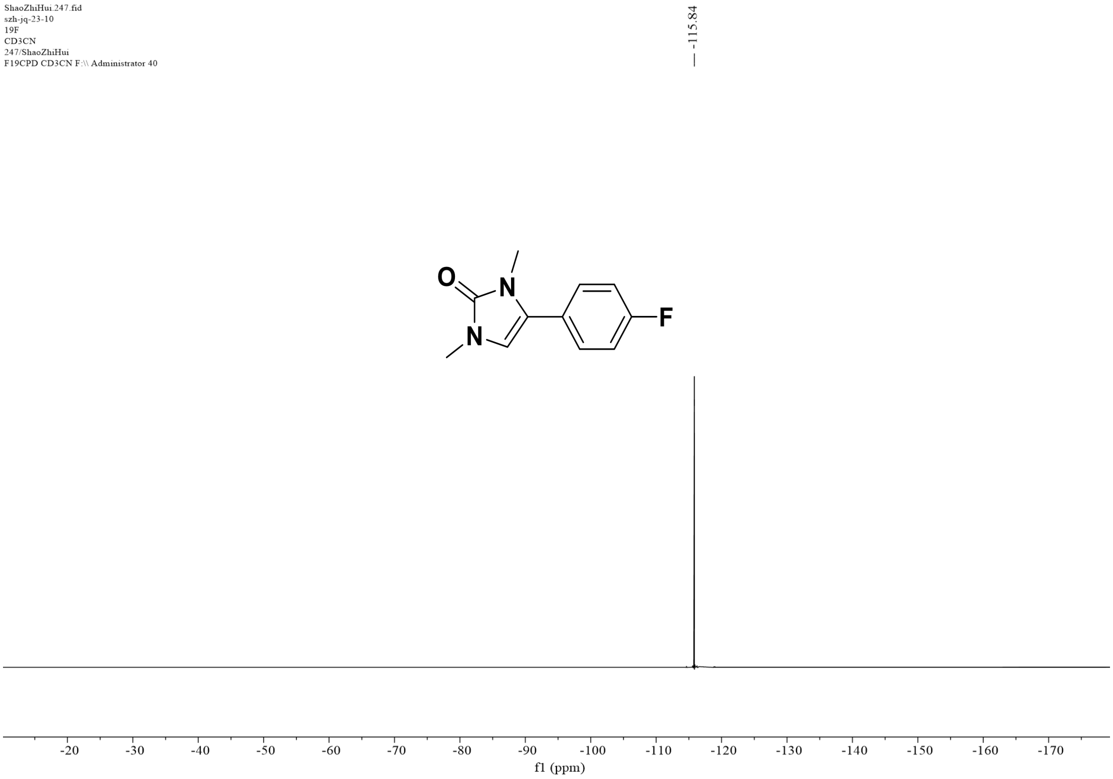

Supplementary Figure 60. ^{1}H NMR (400 MHz, CD_3CN) spectrum of 4n

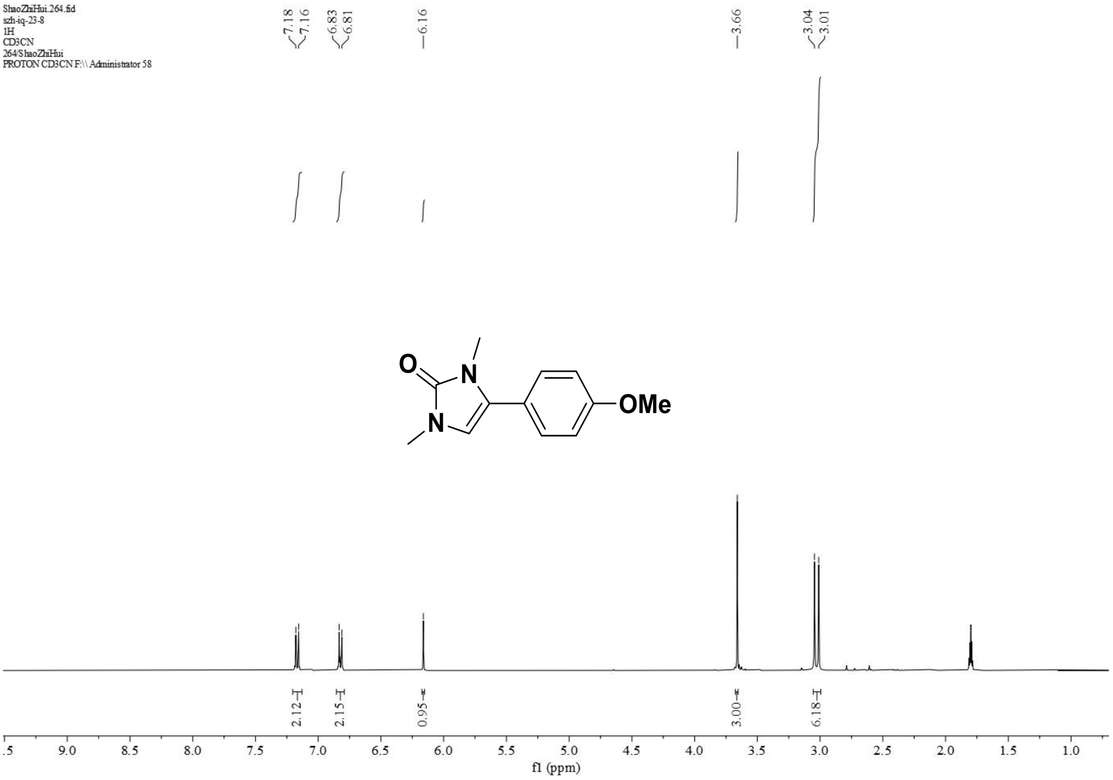

Supplementary Figure 61. ^{13}C NMR (100 MHz, CD_3CN) spectrum of 4n

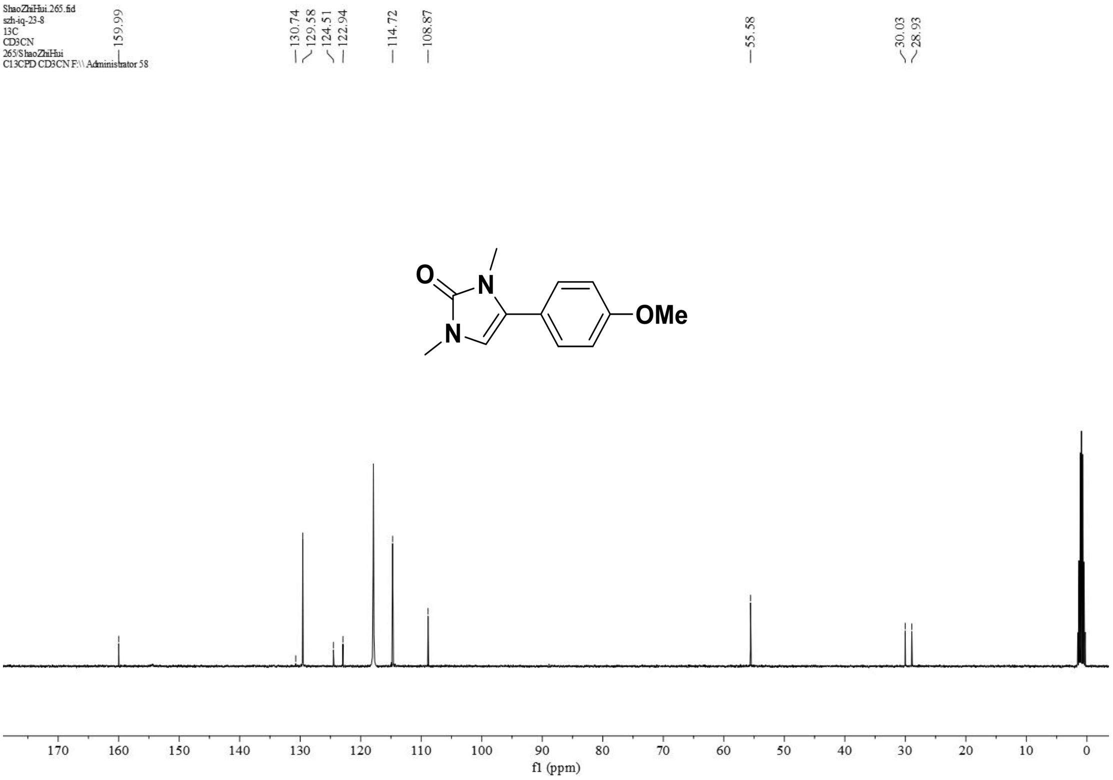

Supplementary Figure 62. ^{1}H NMR (400 MHz, CD_3CN) spectrum of 4o

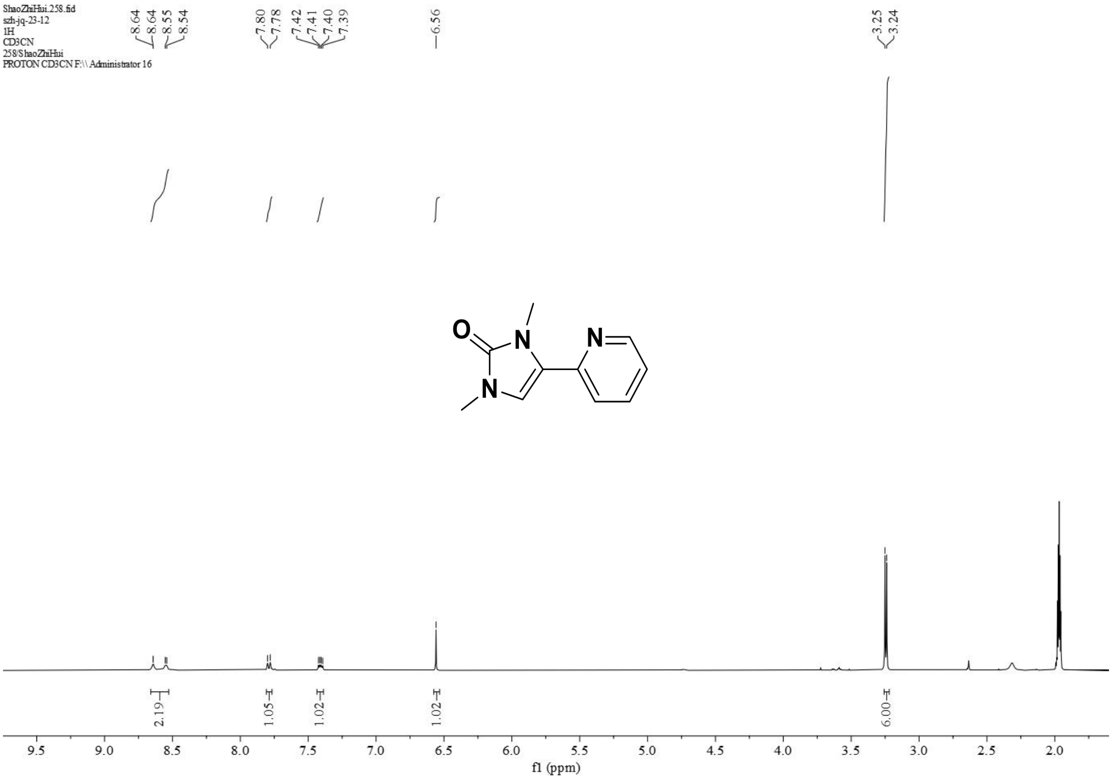

Supplementary Figure 63. ^{13}C NMR (100 MHz, CD_3CN) spectrum of 4o

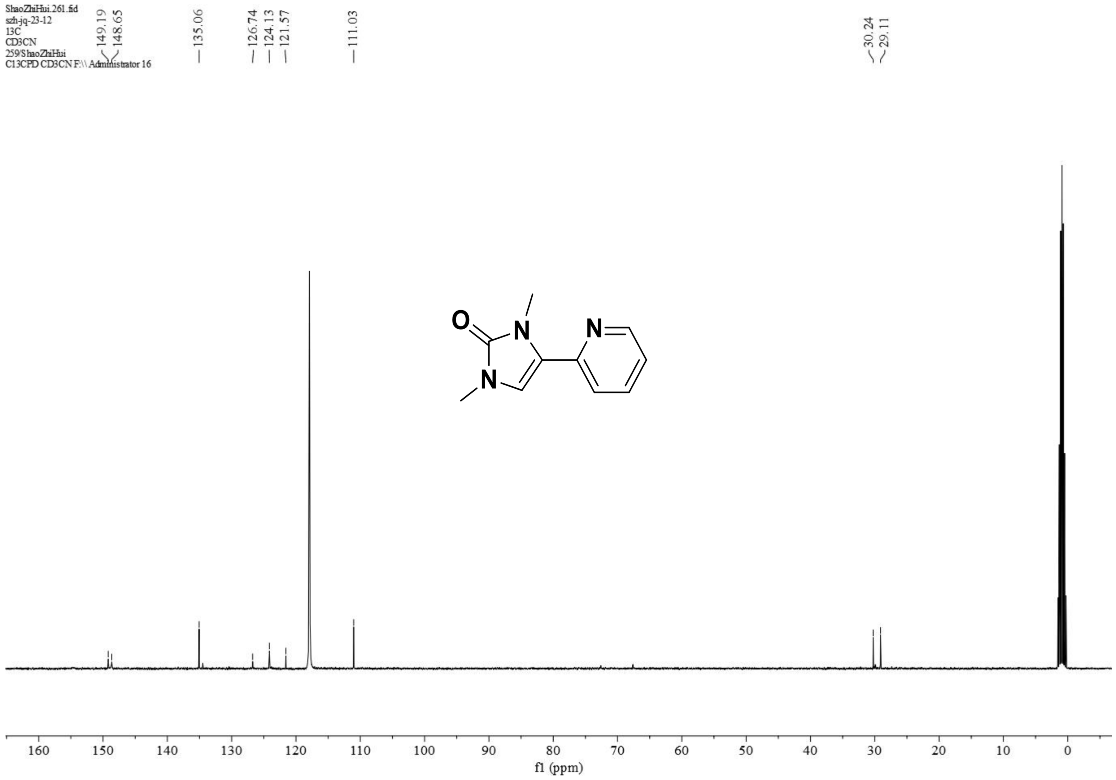

Supplementary Figure 64. ^{1}H NMR (400 MHz, CD_3CN) spectrum of 4p

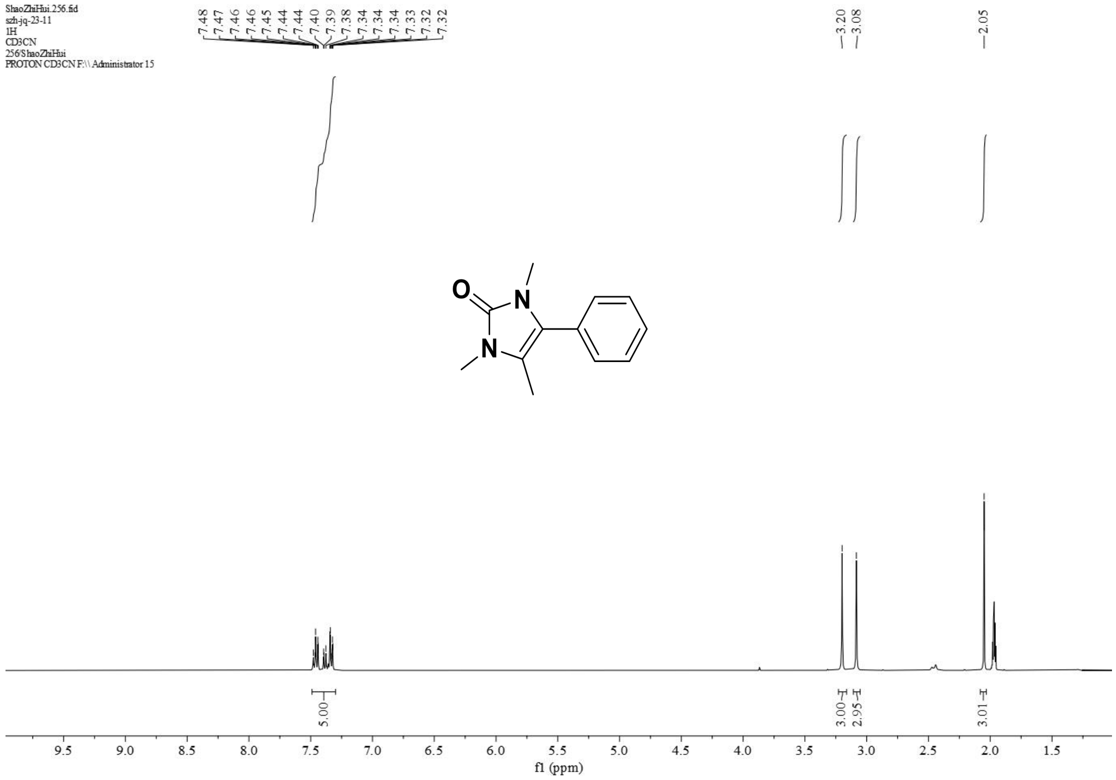

Supplementary Figure 65. ^{13}C NMR (100 MHz, CD_3CN) spectrum of 4p

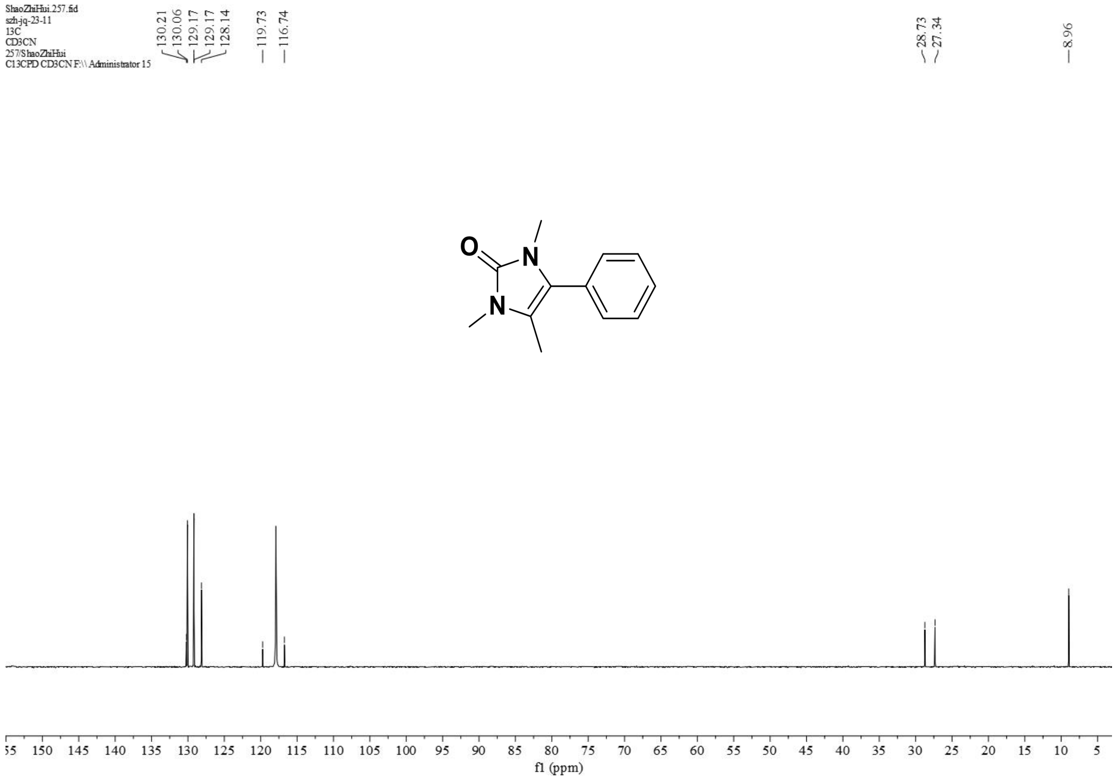

Supplementary Figure 66. ^{1}H NMR (400 MHz, CD_3CN) spectrum of 4q

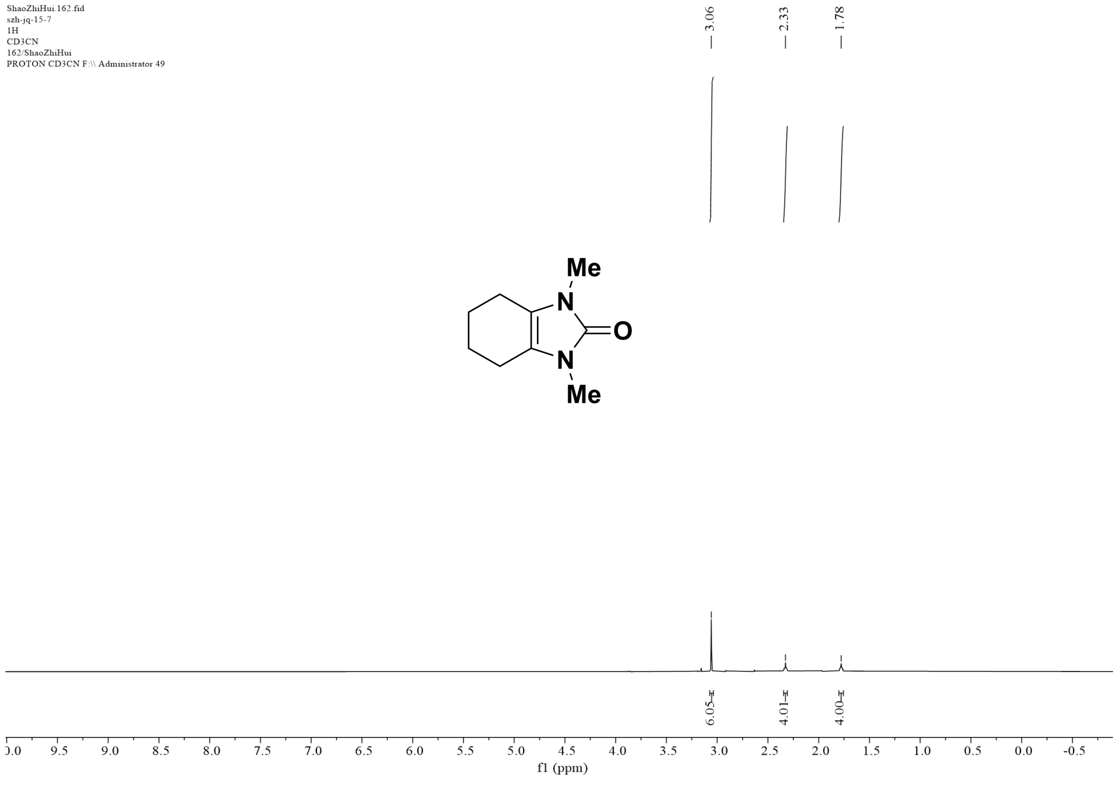

Supplementary Figure 67. ^{13}C NMR (100 MHz, CD_3CN) spectrum of 4q

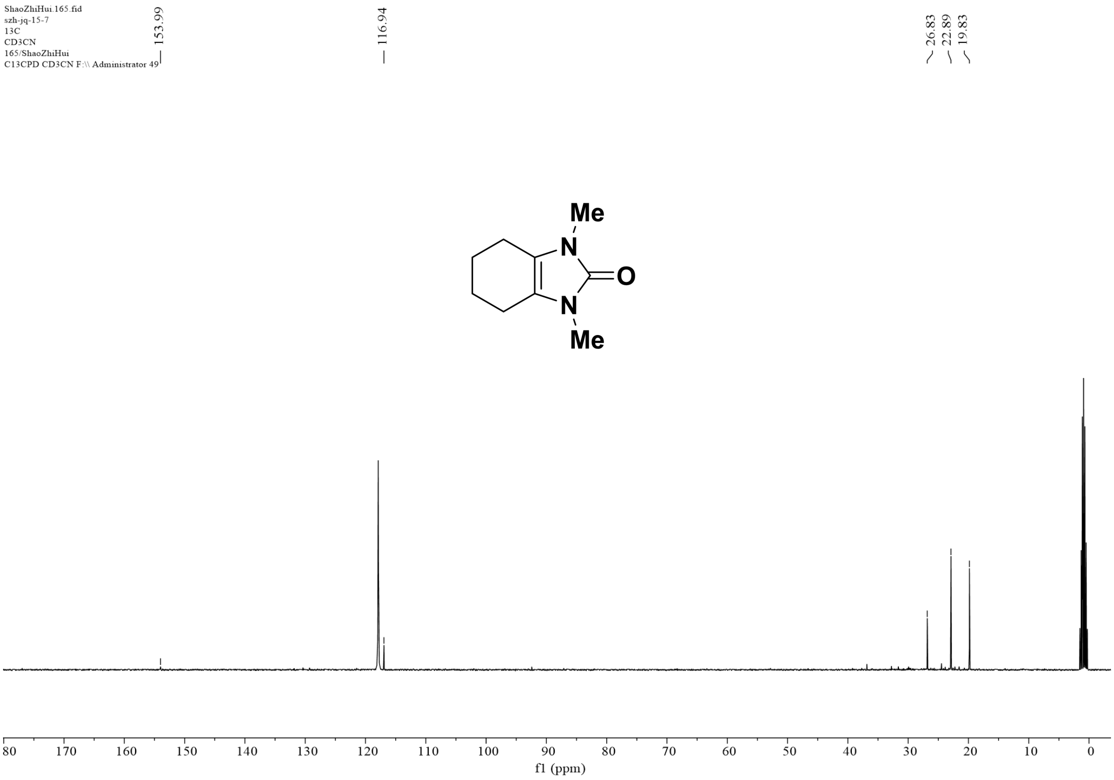

Supplementary Figure 68. ^{1}H NMR (400 MHz, CD_3CN) spectrum of 4r

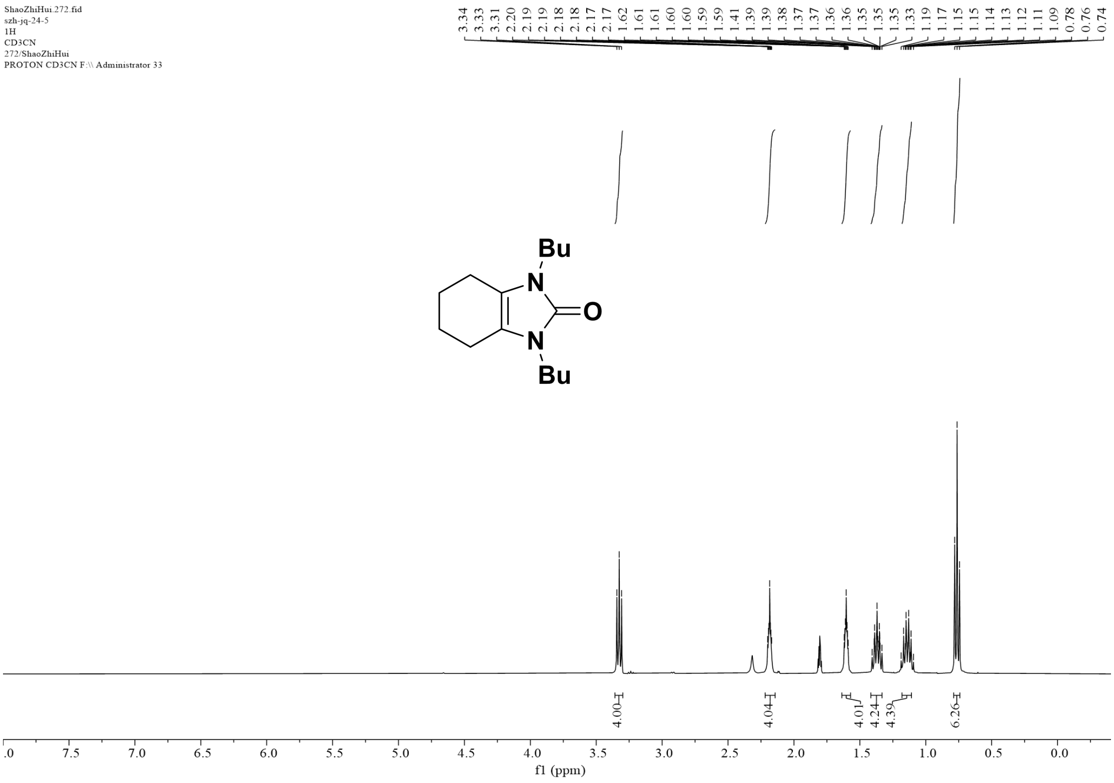

Supplementary Figure 69. ^{13}C NMR (100 MHz, CD_3CN) spectrum of 4r

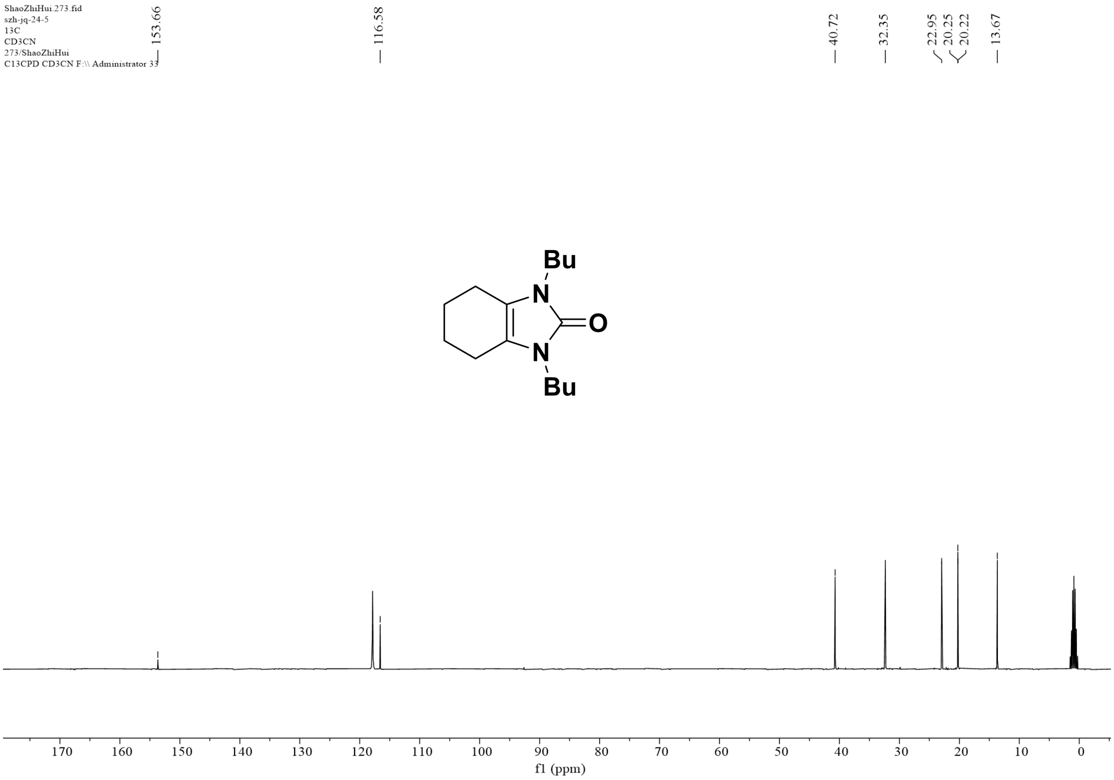

Supplementary Figure 70. ^{1}H NMR (400 MHz, CD_3CN) spectrum of 4s

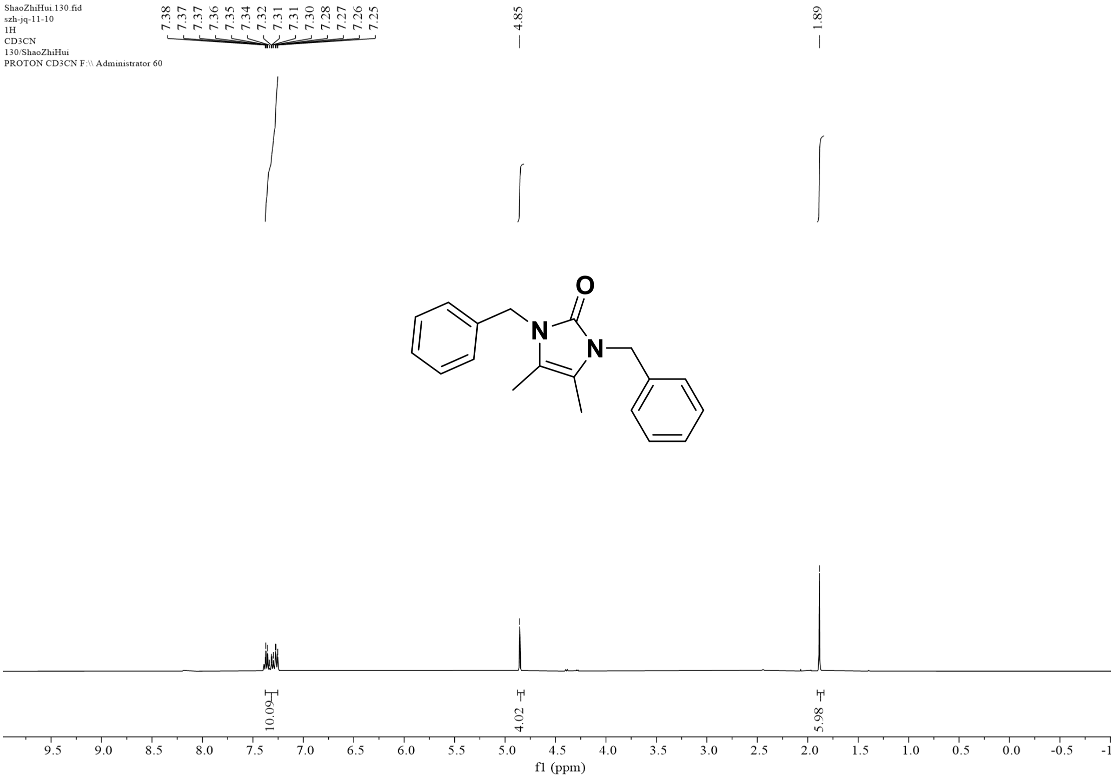

Supplementary Figure 71. ^{13}C NMR (100 MHz, CD_3CN) spectrum of 4s

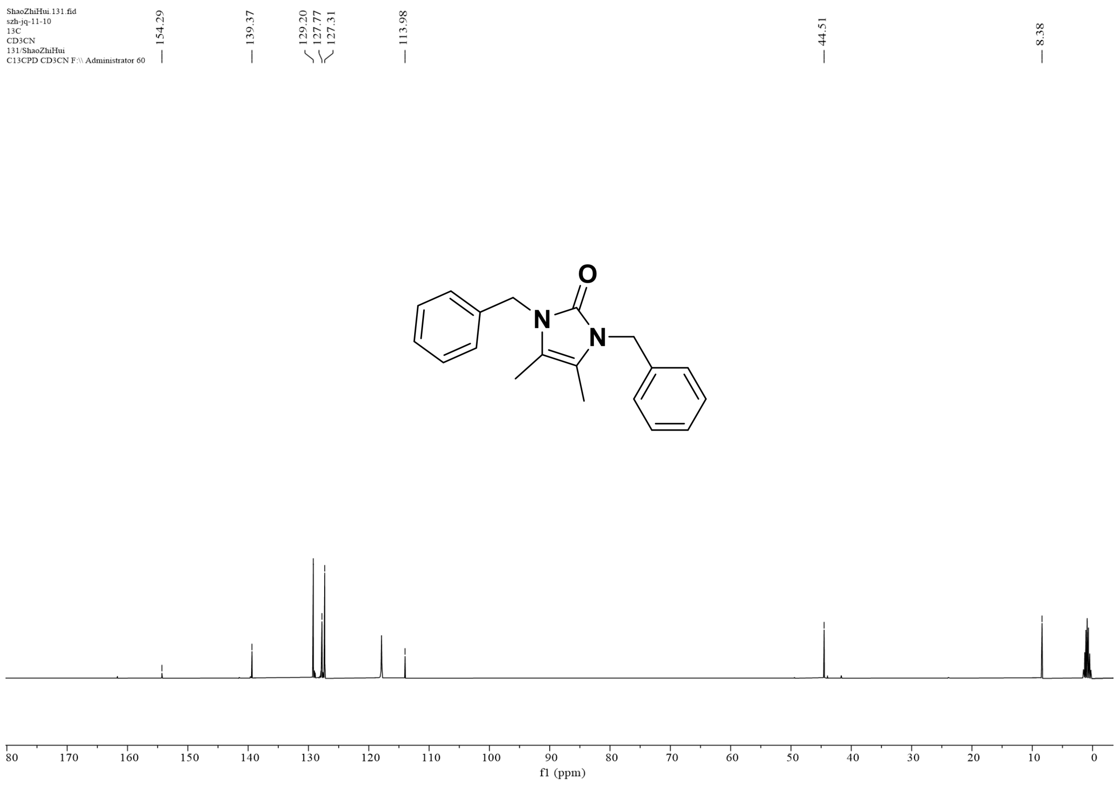

Supplementary Figure 72. ^{1}H NMR (400 MHz, CD_3CN) spectrum of 4t

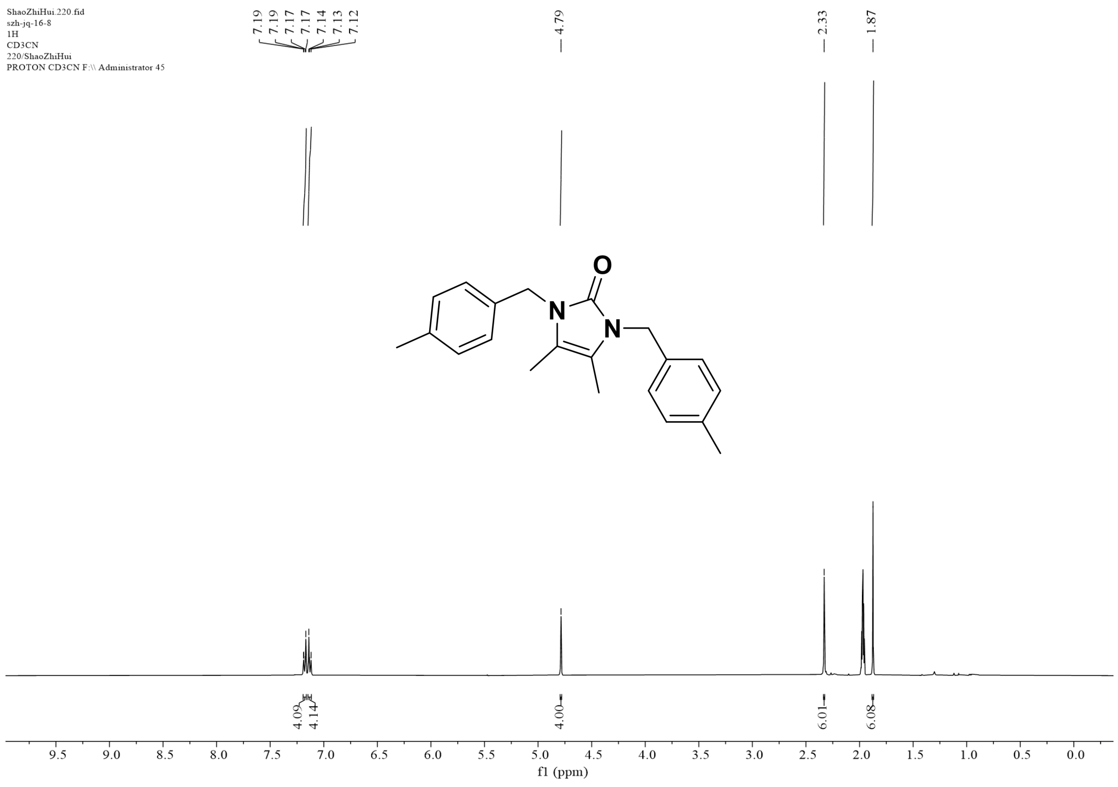

Supplementary Figure 73. ^{13}C NMR (100 MHz, CD_3CN) spectrum of 4t

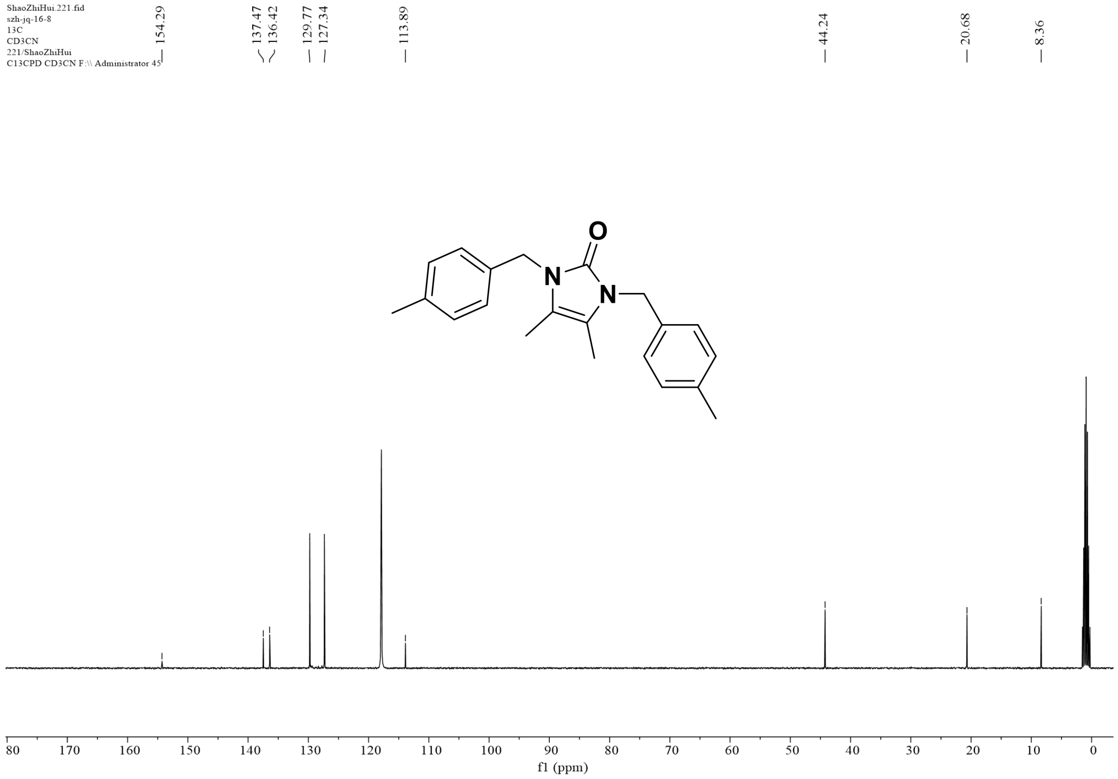

Supplementary Figure 74. ^{1}H NMR (400 MHz, CD_3CN) spectrum of 4u

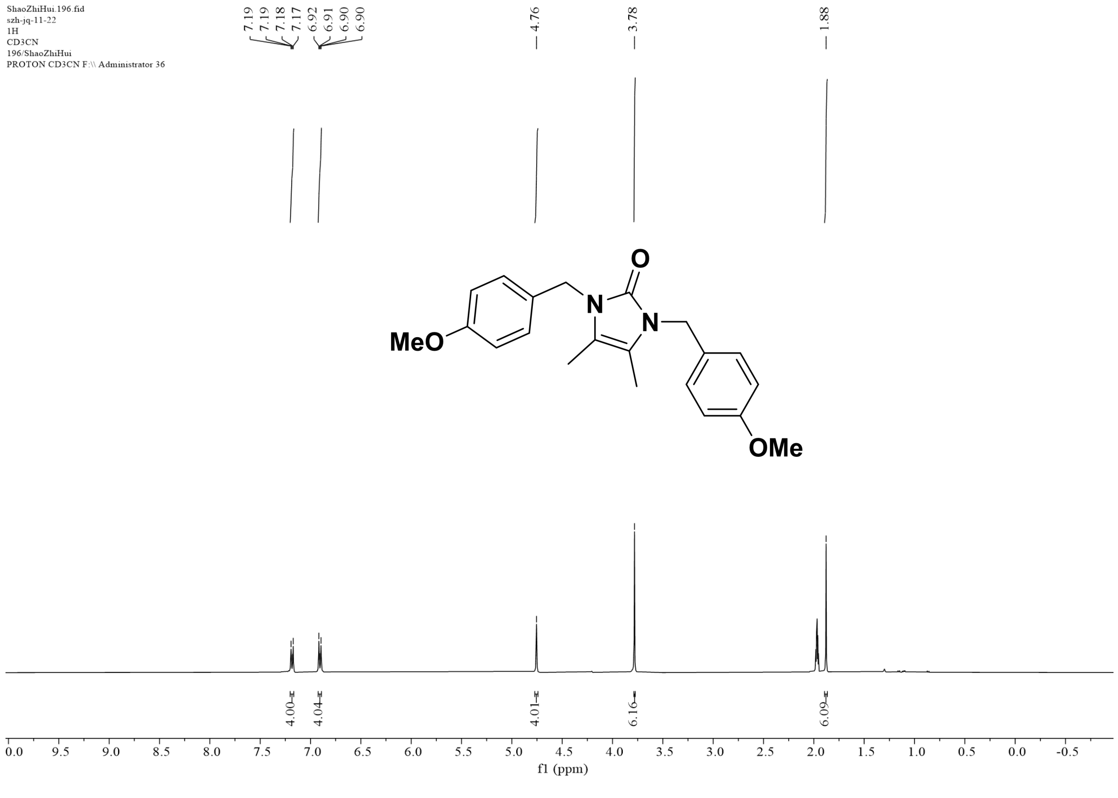

Supplementary Figure 75. ^{13}C NMR (100 MHz, CD_3CN) spectrum of 4u

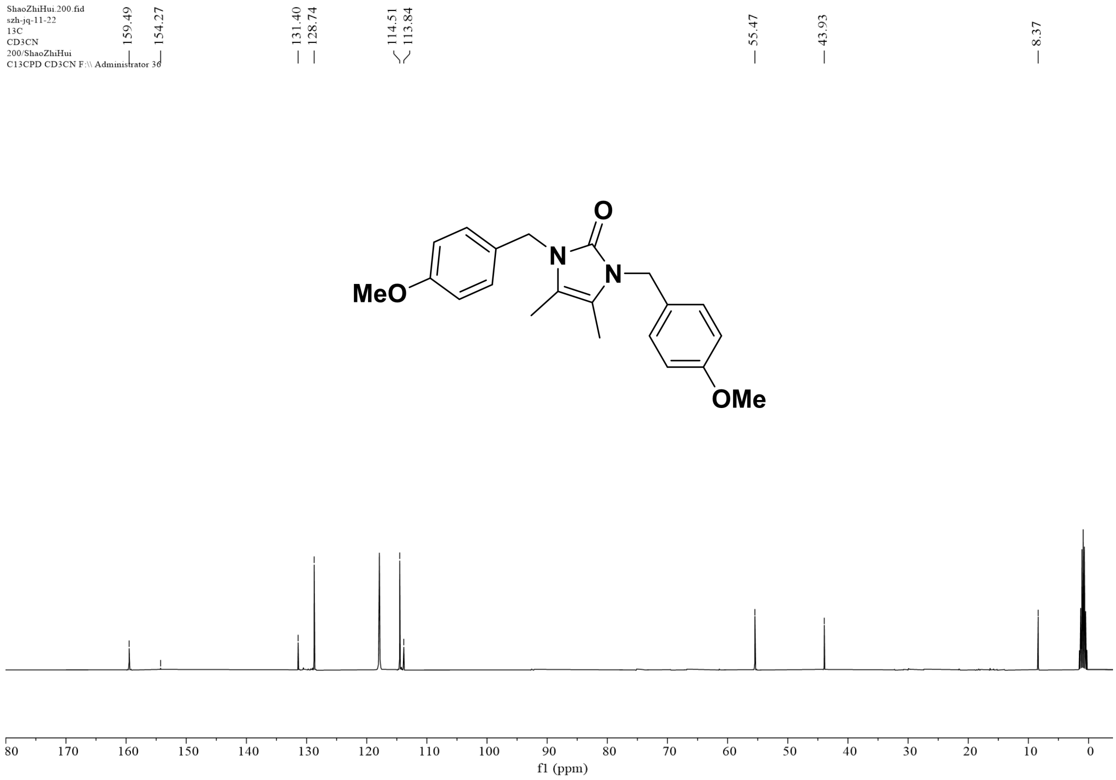

Supplementary Figure 76. ^{1}H NMR (400 MHz, CD_3CN) spectrum of 4v

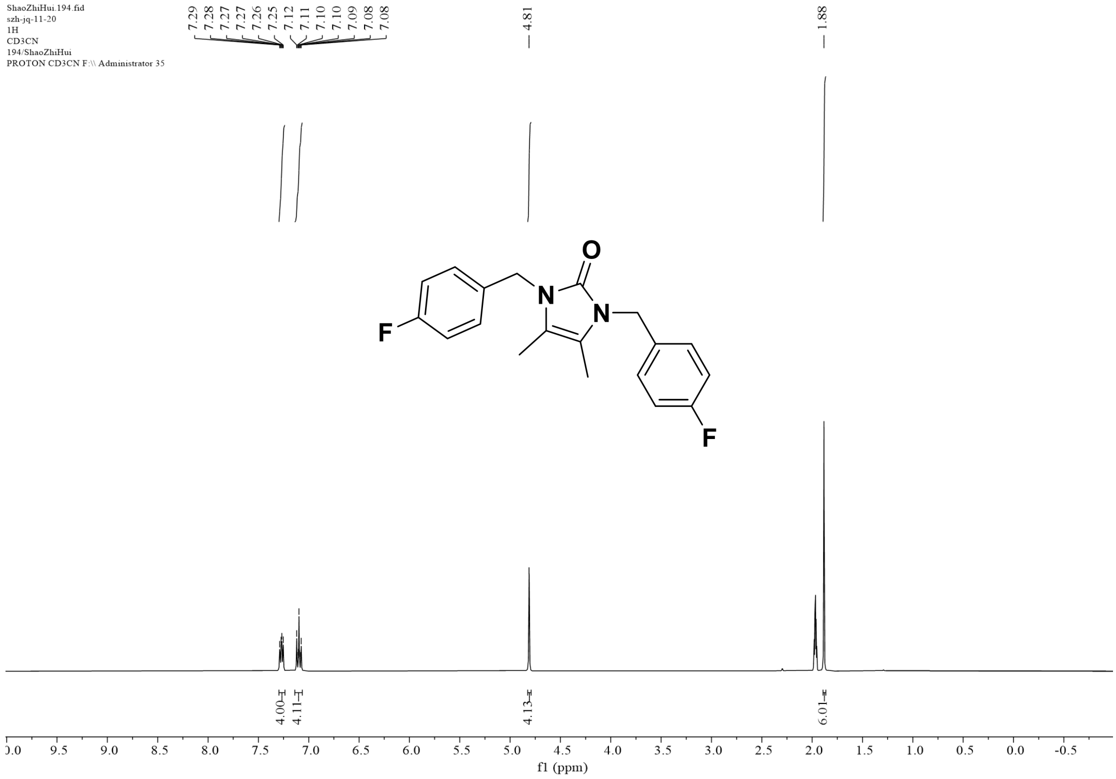

Supplementary Figure 77. ^{13}C NMR (100 MHz, CD_3CN) spectrum of 4v

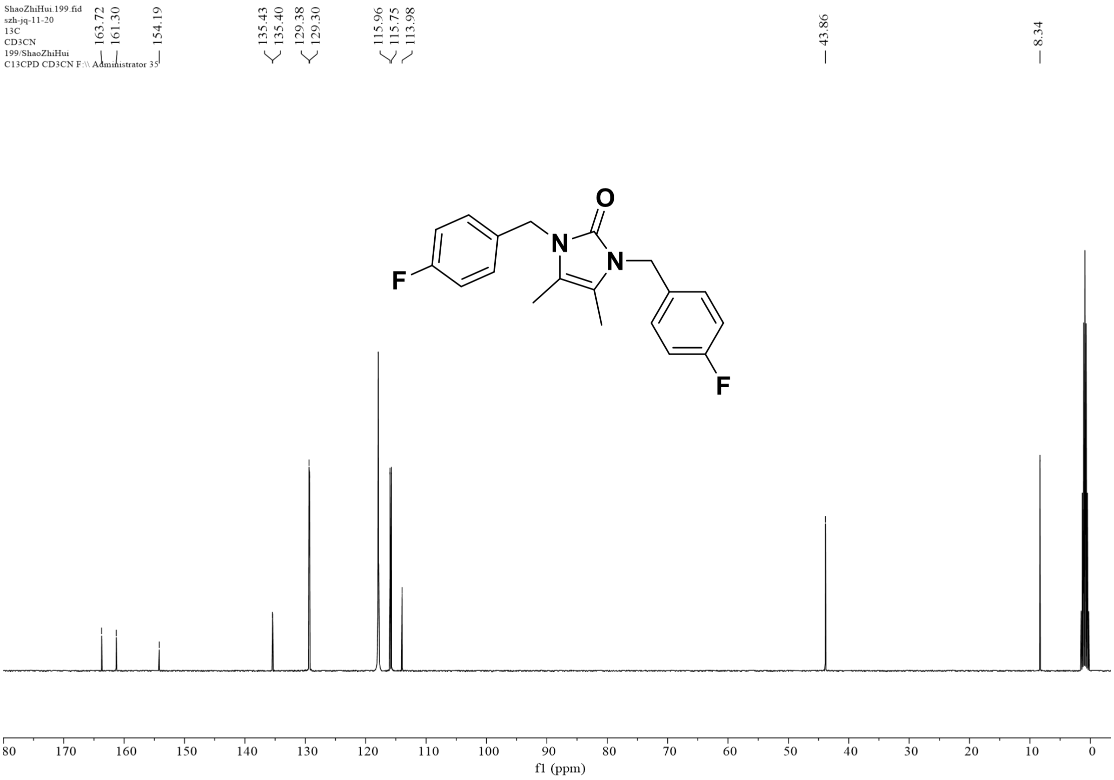

Supplementary Figure 78. ^{19}F NMR (376 MHz, CD_3CN) spectrum of 4v

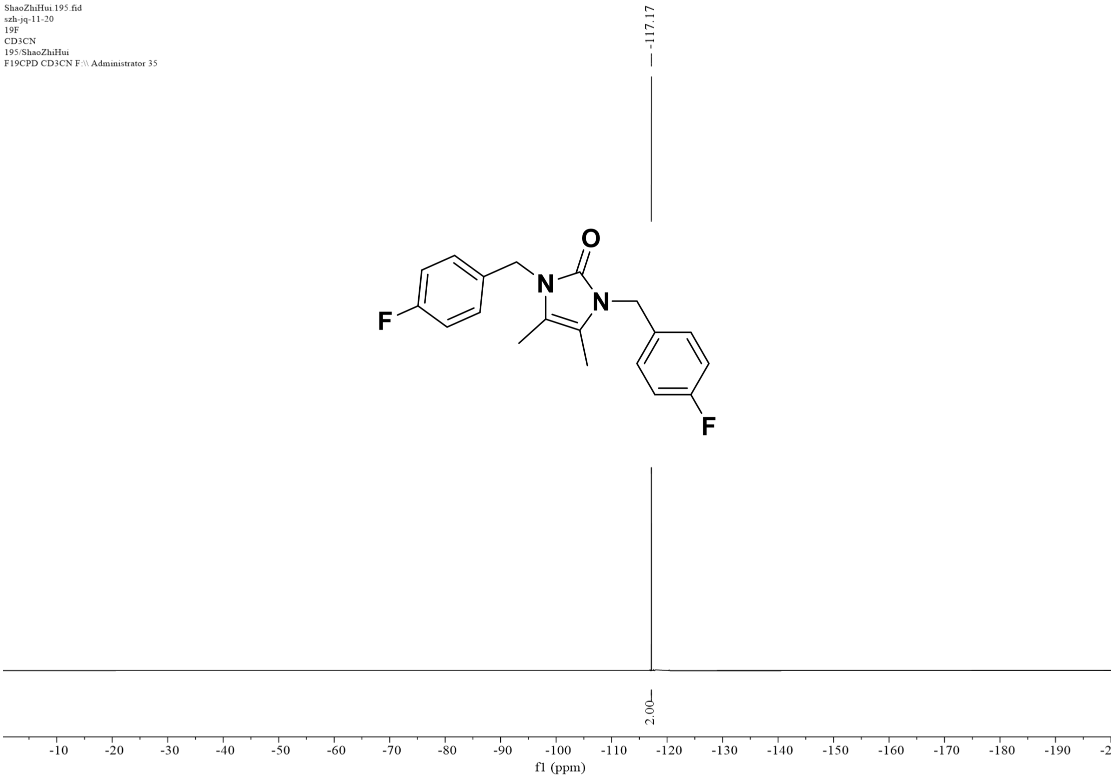

Supplementary Figure 79. ^{1}H NMR (400 MHz, CD_3CN) spectrum of 4w

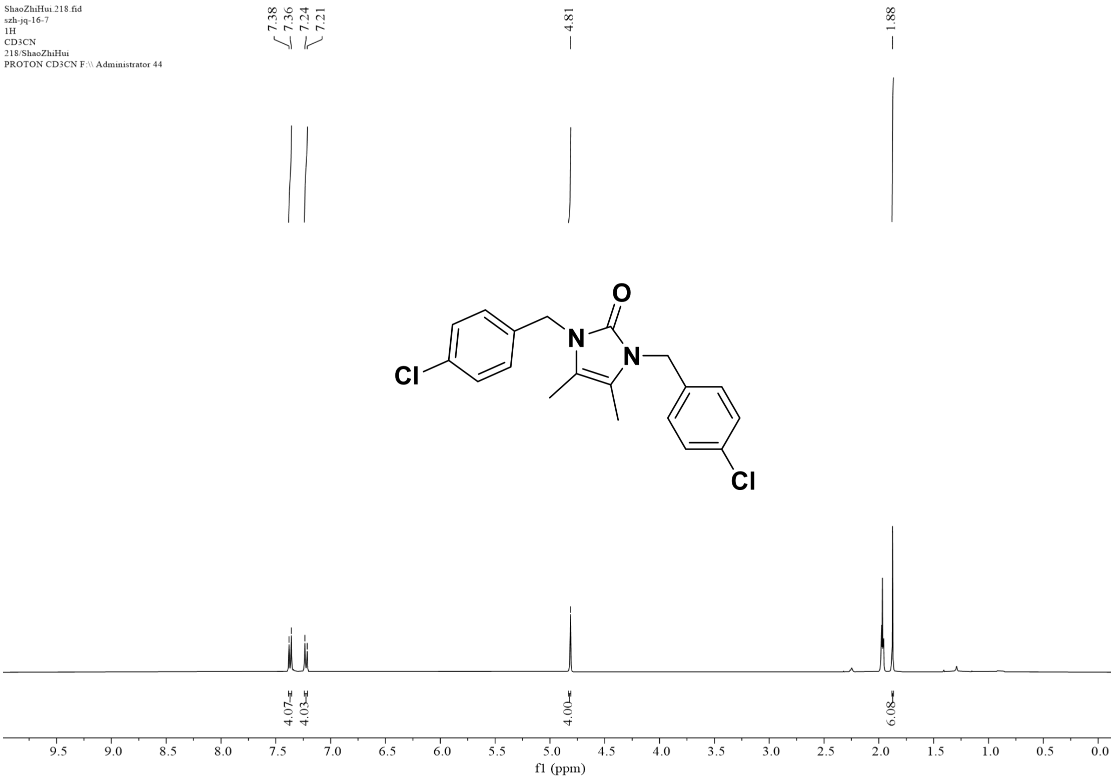

Supplementary Figure 80. ^{13}C NMR (100 MHz, CD_3CN) spectrum of 4w

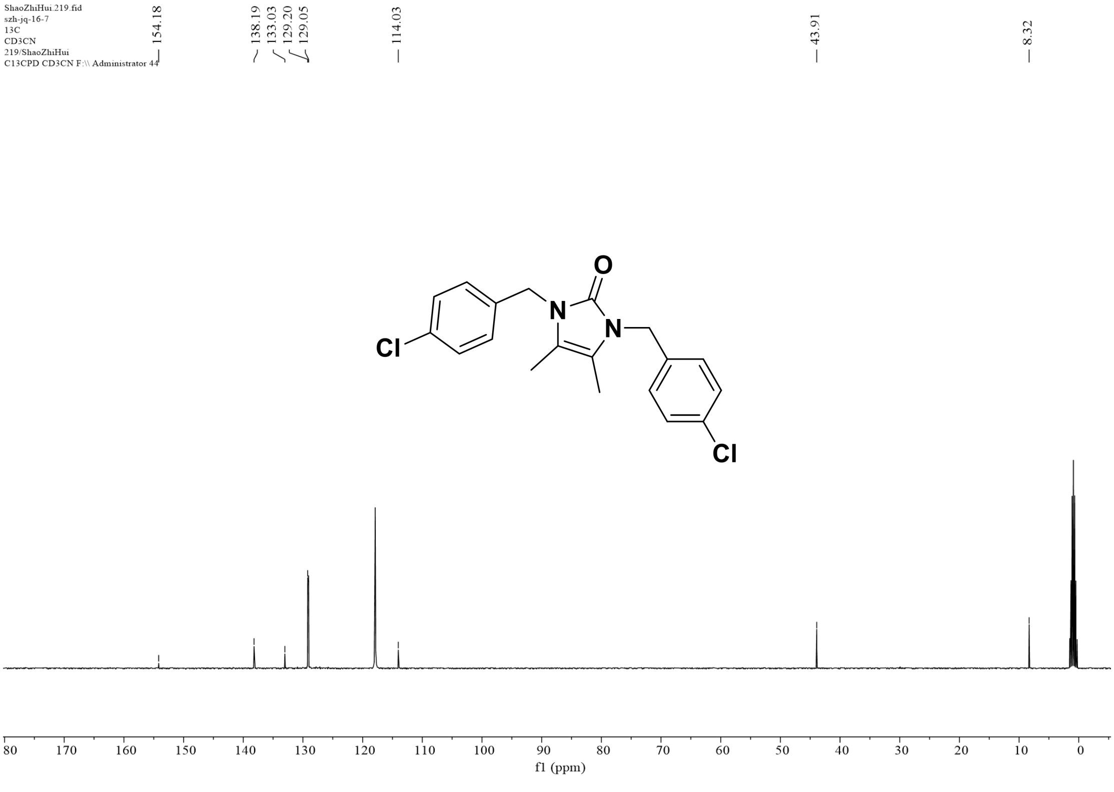

Supplementary Figure 81. ^{1}H NMR (400 MHz, CD_3CN) spectrum of 4x

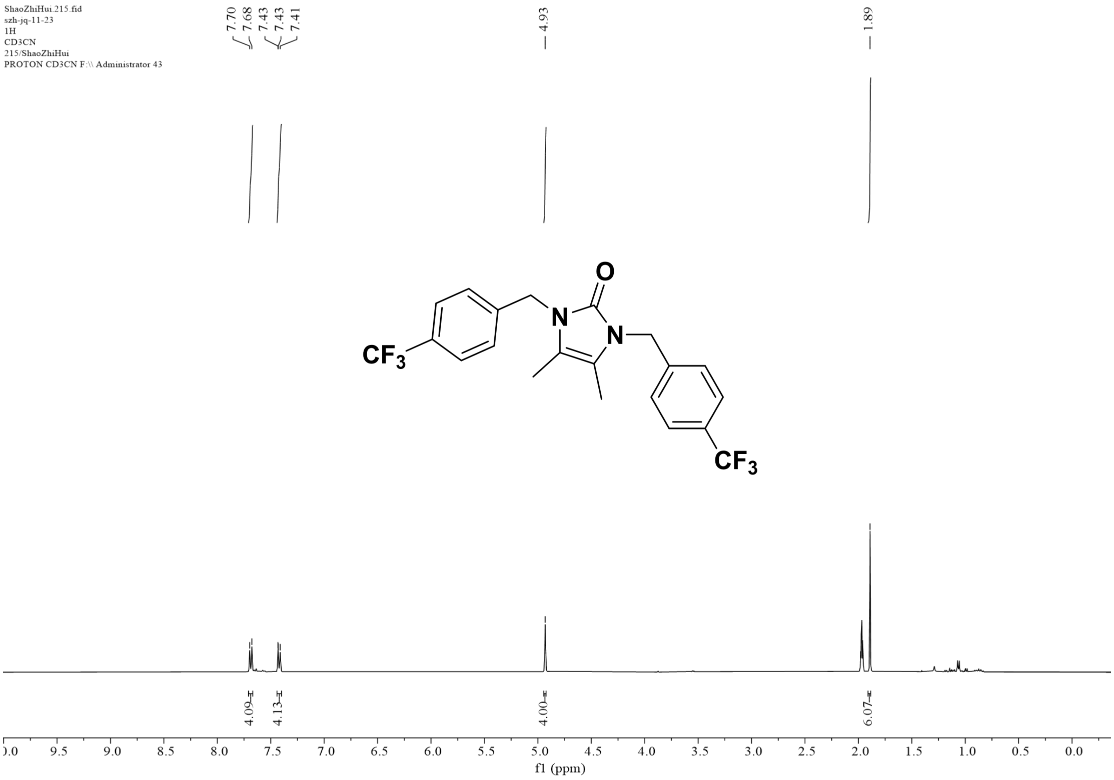

Supplementary Figure 82. ^{13}C NMR (100 MHz, CD_3CN) spectrum of 4x

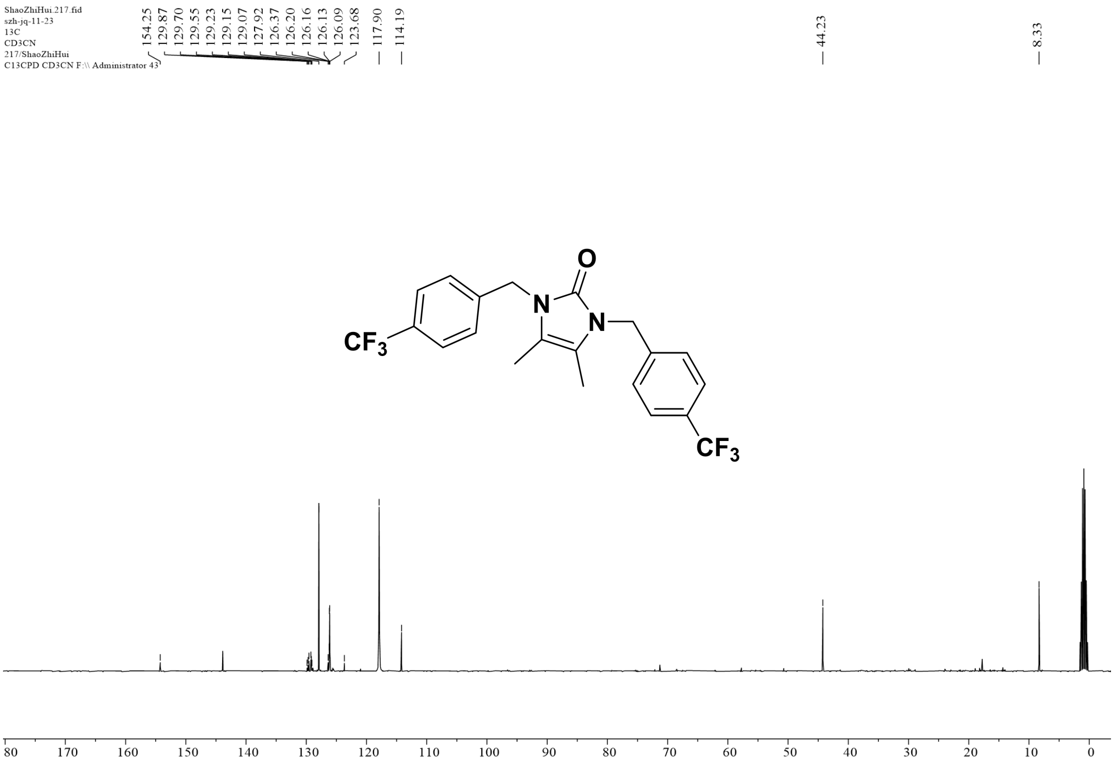

Supplementary Figure 83. ^{19}F NMR (376 MHz, CD3CN) spectrum of 4x

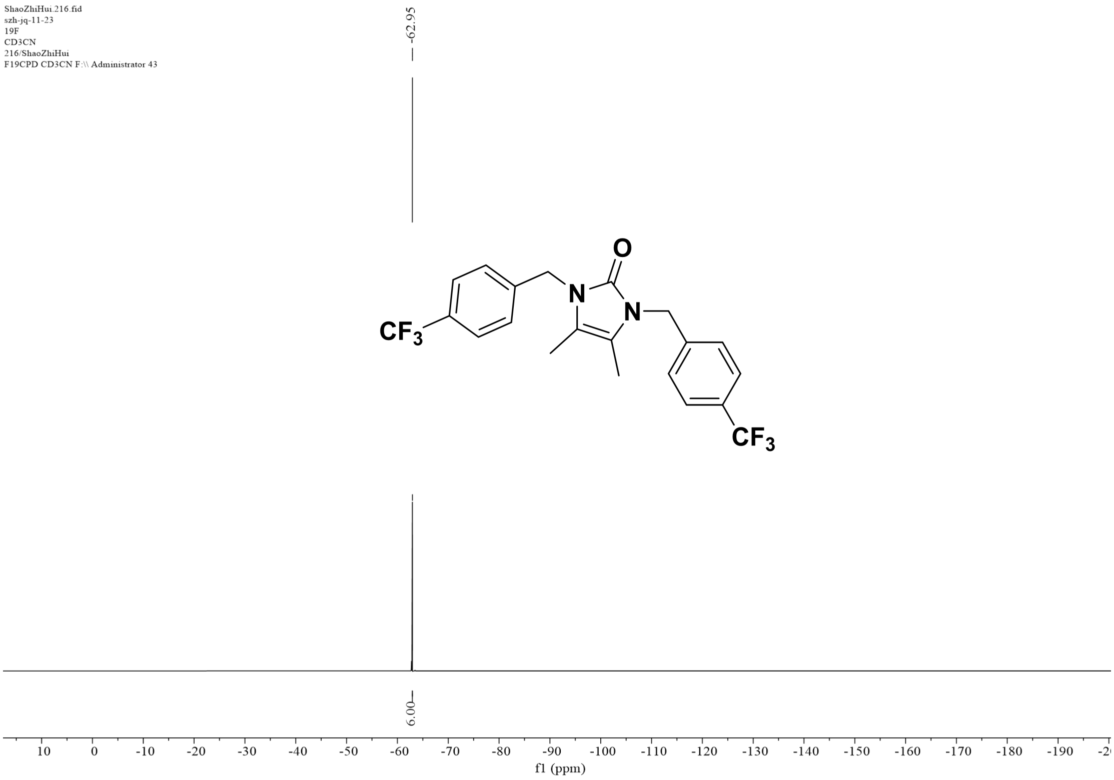

Supplementary Figure 84. ^{1}H NMR (400 MHz, CD_3CN) spectrum of (4y+4y')

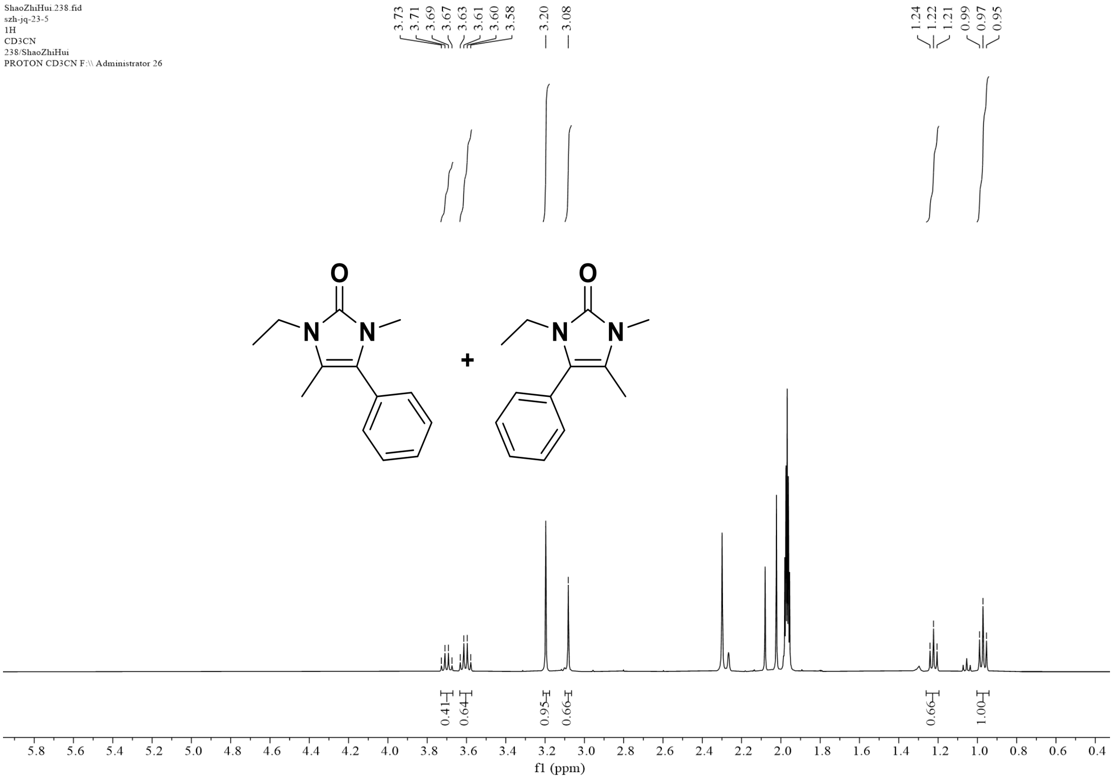

Supplementary Figure 85. ^{1}H NMR (400 MHz, DMSO) spectrum of 1t

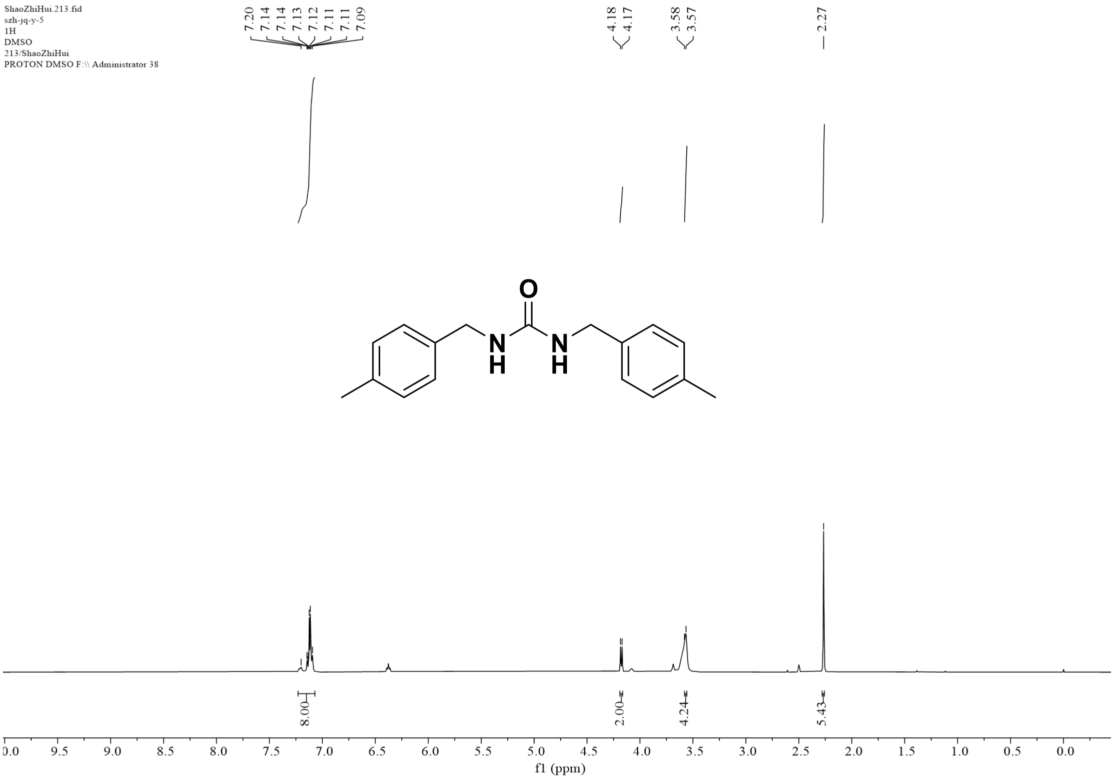

Supplementary Figure 86. ^{13}C NMR (100 MHz, DMSO) spectrum of 1t

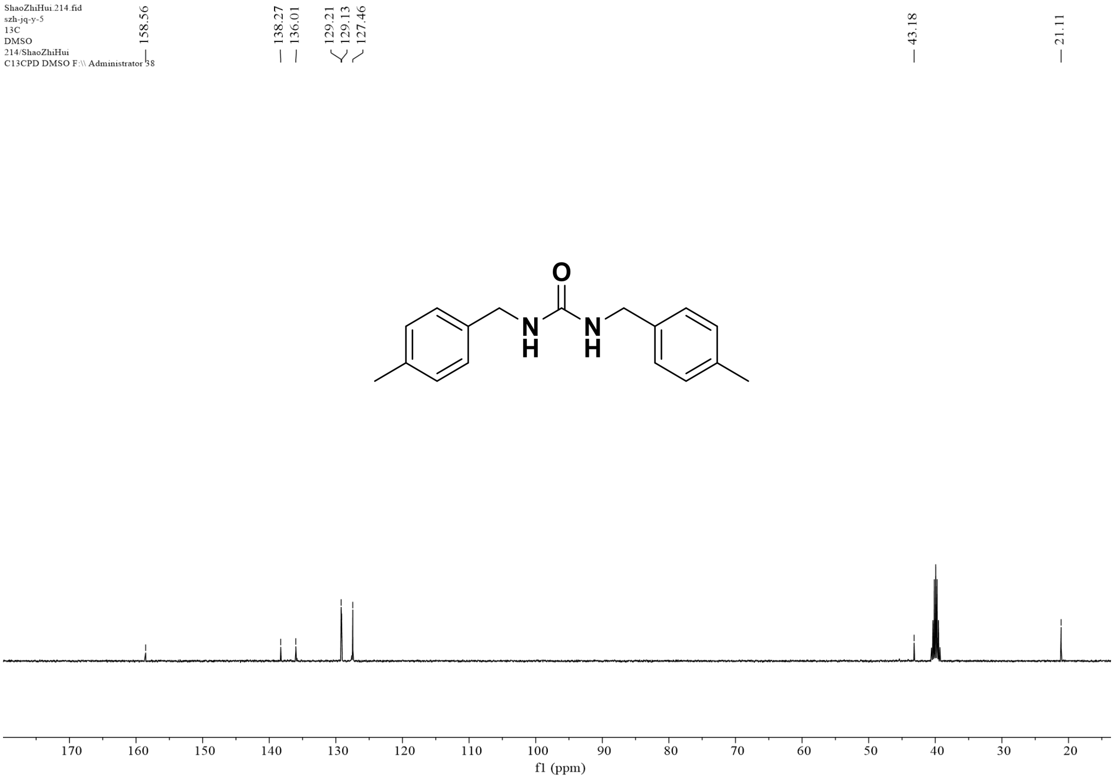

Supplementary Figure 87. ^{1}H NMR (400 MHz, DMSO) spectrum of 1u

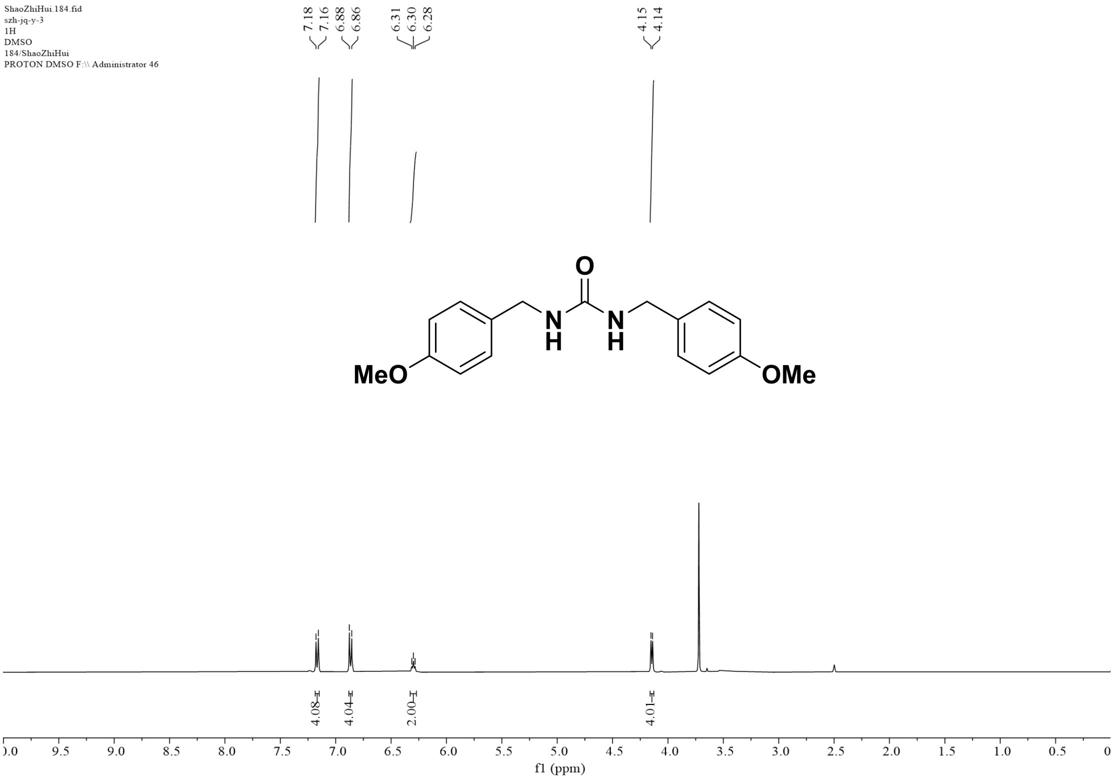

Supplementary Figure 88. ^{13}C NMR (100 MHz, DMSO) spectrum of 1u

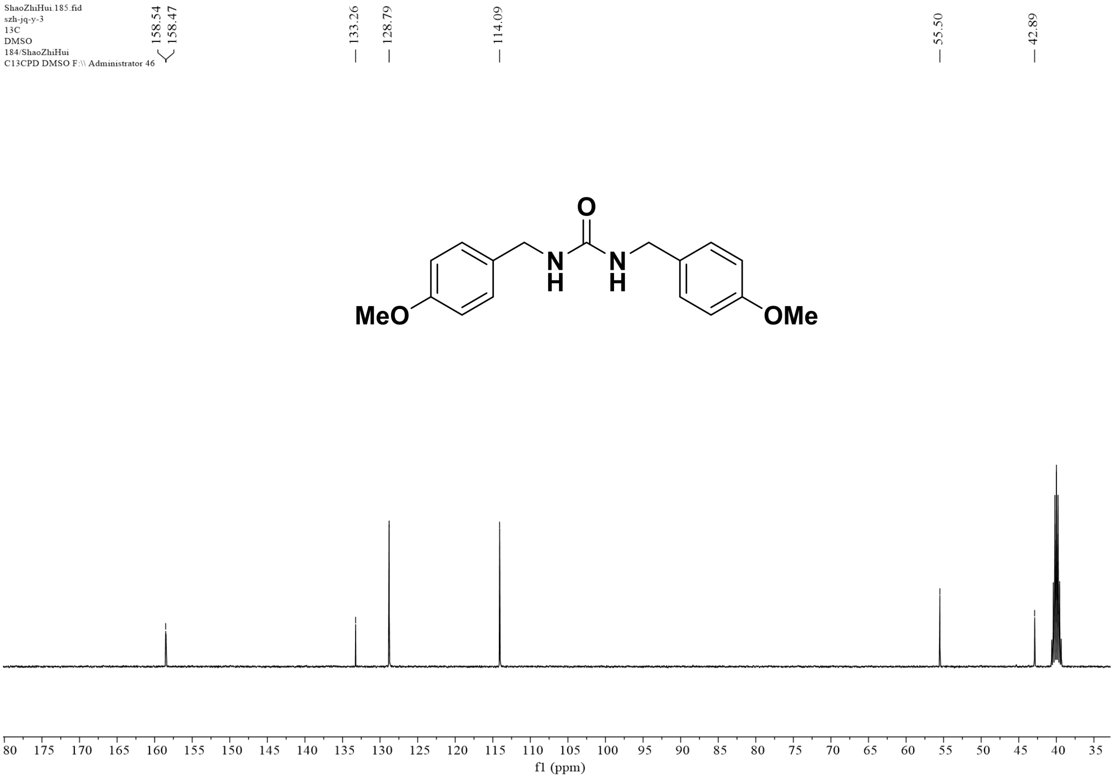

Supplementary Figure 89. ^{1}H NMR (400 MHz, DMSO) spectrum of 1v

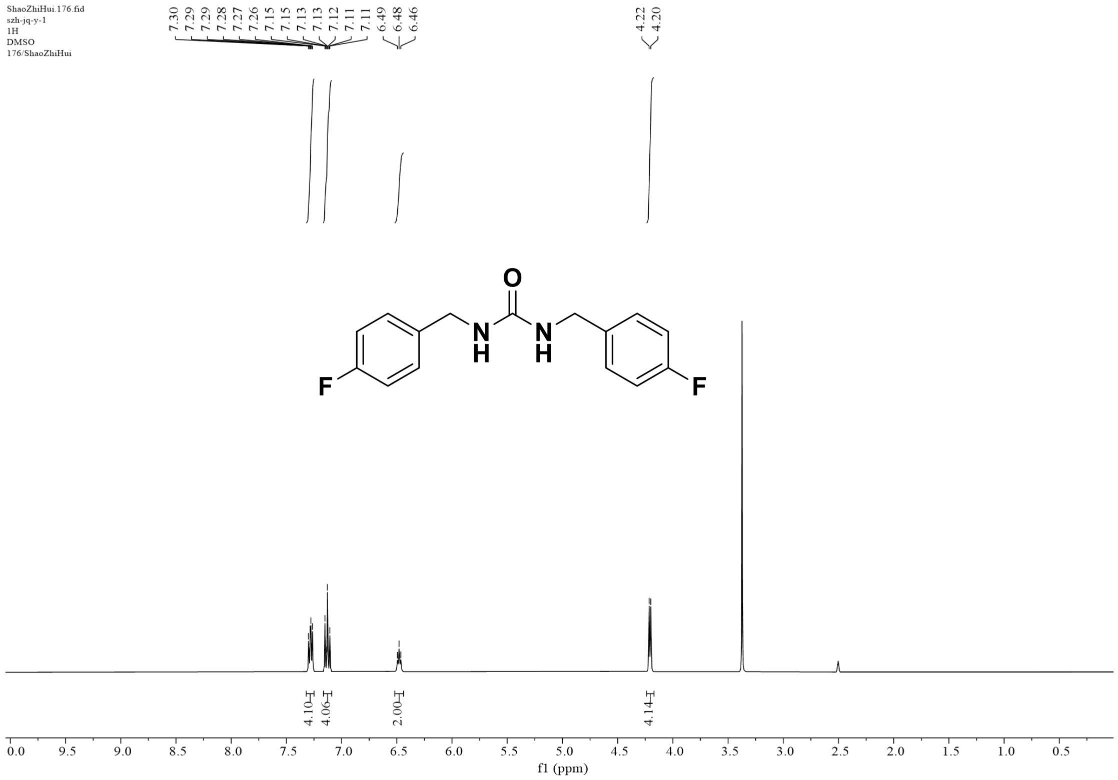

Supplementary Figure 90. ^{13}C NMR (100 MHz, DMSO) spectrum of 1v

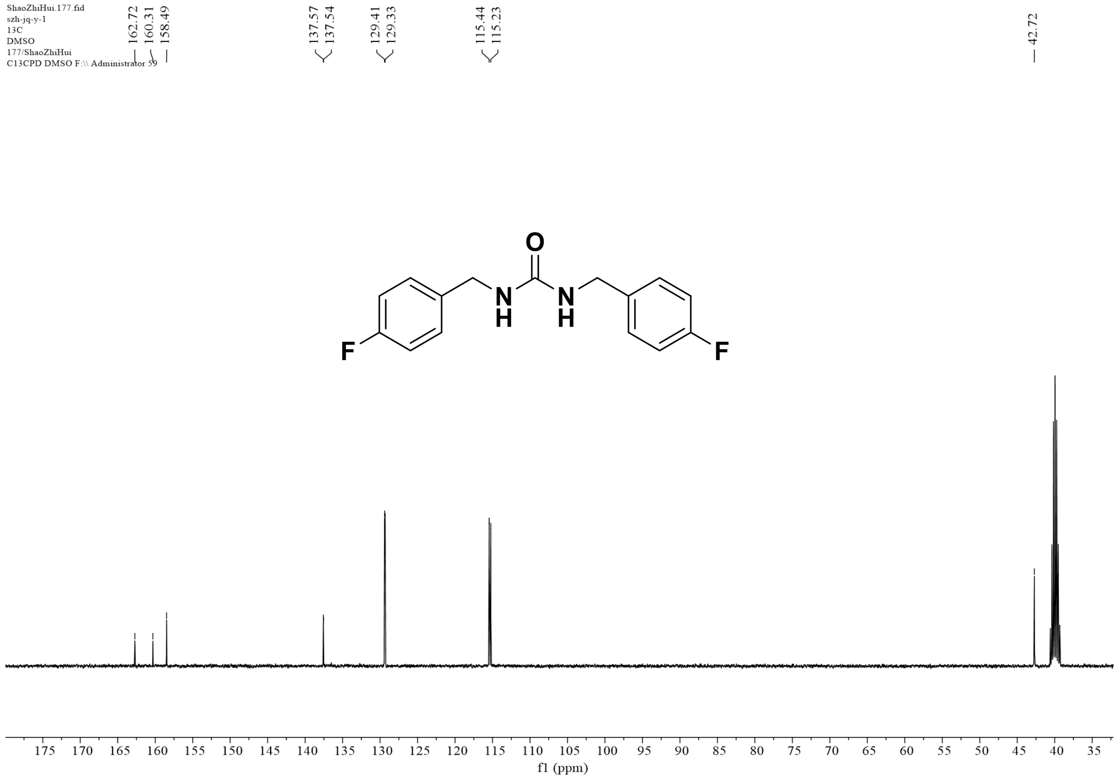

Supplementary Figure 91. ^{19}F NMR (376 MHz, CD3CN) spectrum of 1v

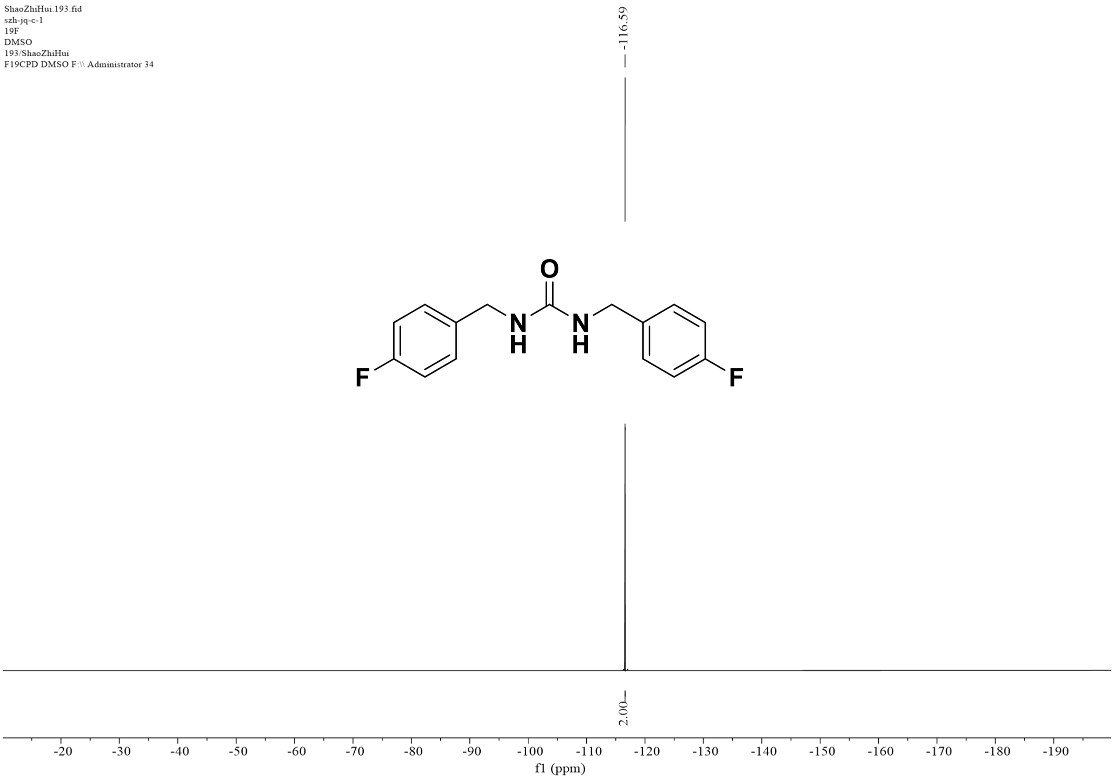

Supplementary Figure 92. ^{1}H NMR (400 MHz, DMSO) spectrum of 1w

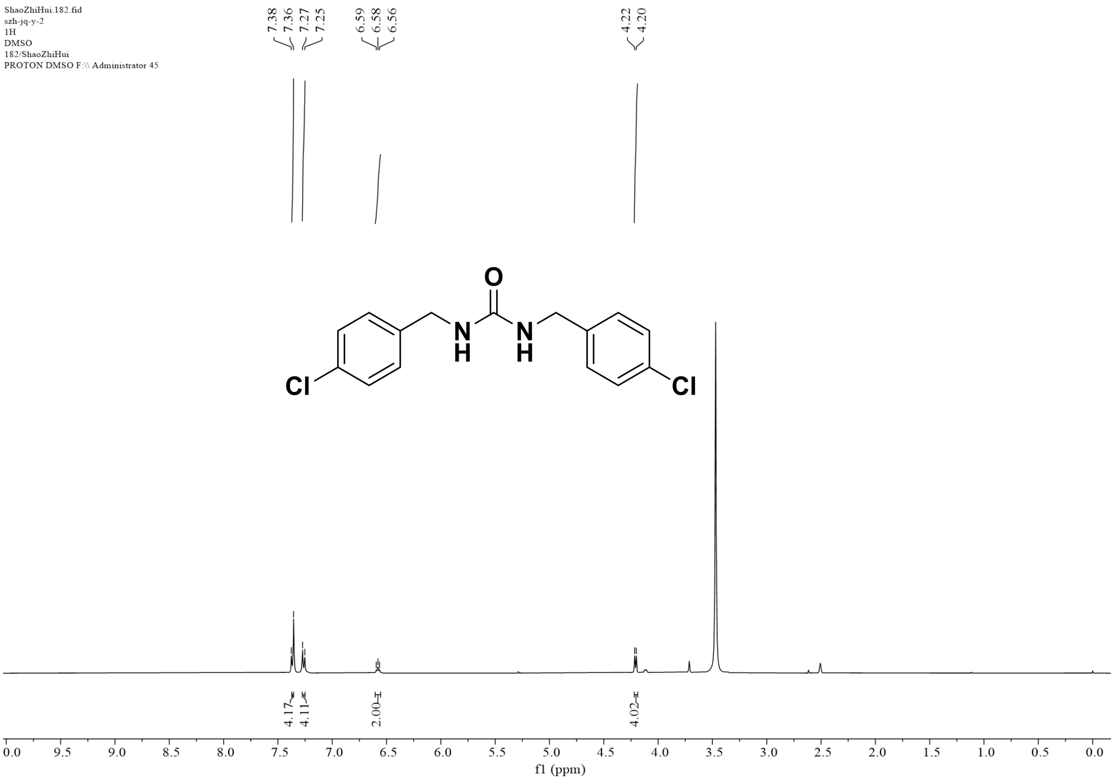

Supplementary Figure 93. ^{13}C NMR (100 MHz, DMSO) spectrum of 1w

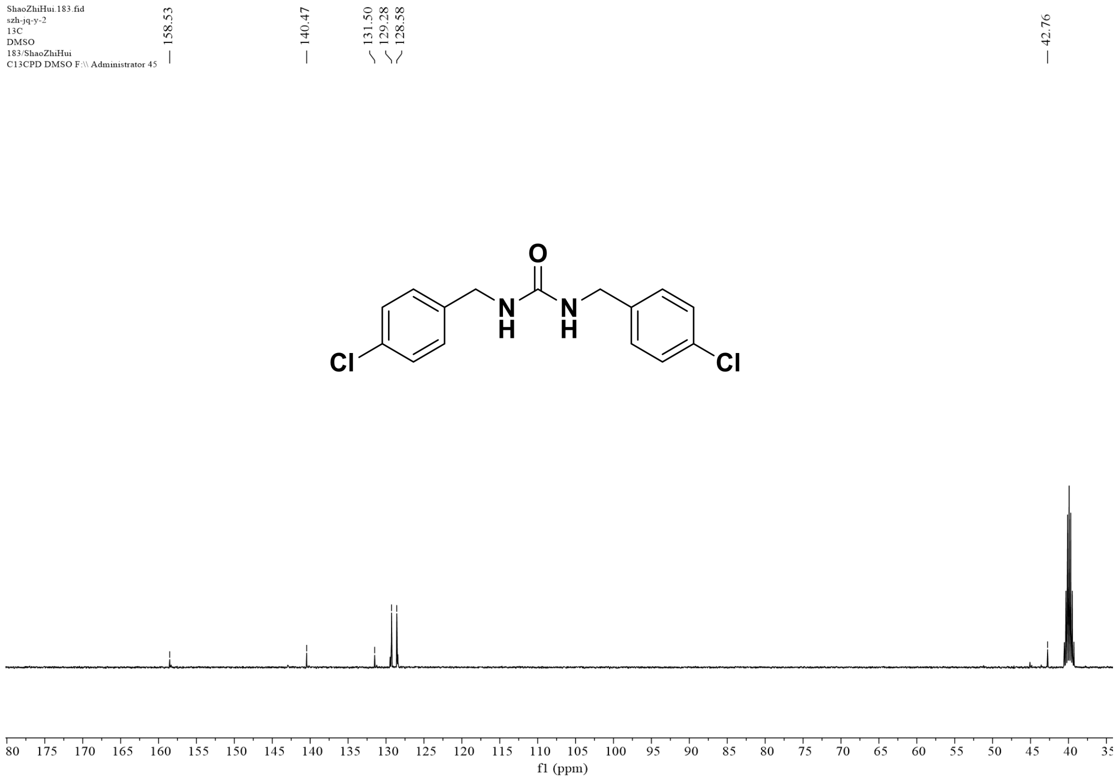

Supplementary Figure 94. ^{1}H NMR (400 MHz, DMSO) spectrum of 1x

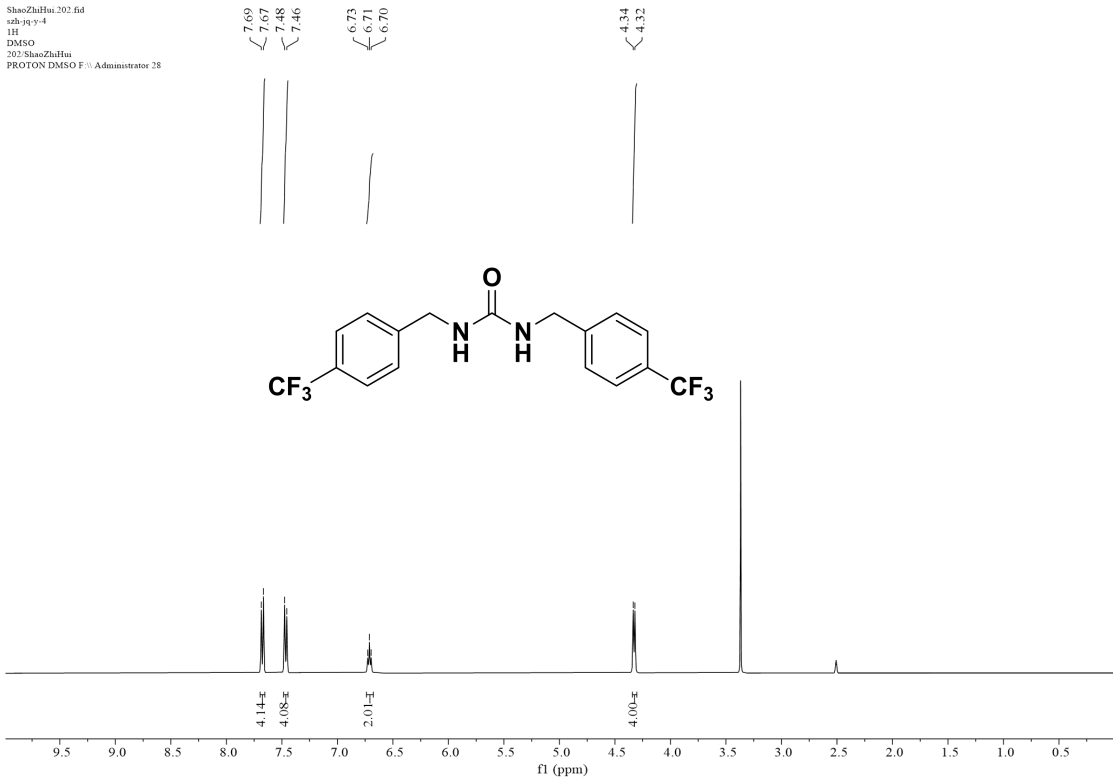

Supplementary Figure 95. ^{13}C NMR (100 MHz, DMSO) spectrum of 1x

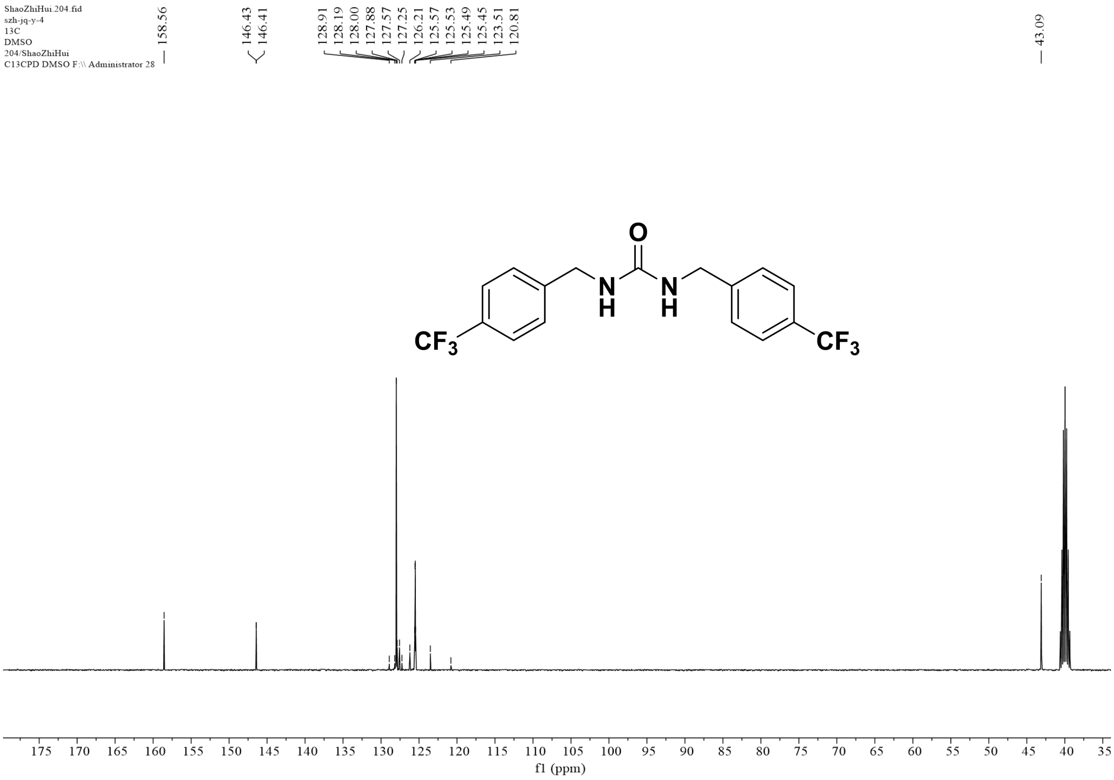

Supplementary Figure 96. ^{19}F NMR (376 MHz, CD3CN) spectrum of 1x

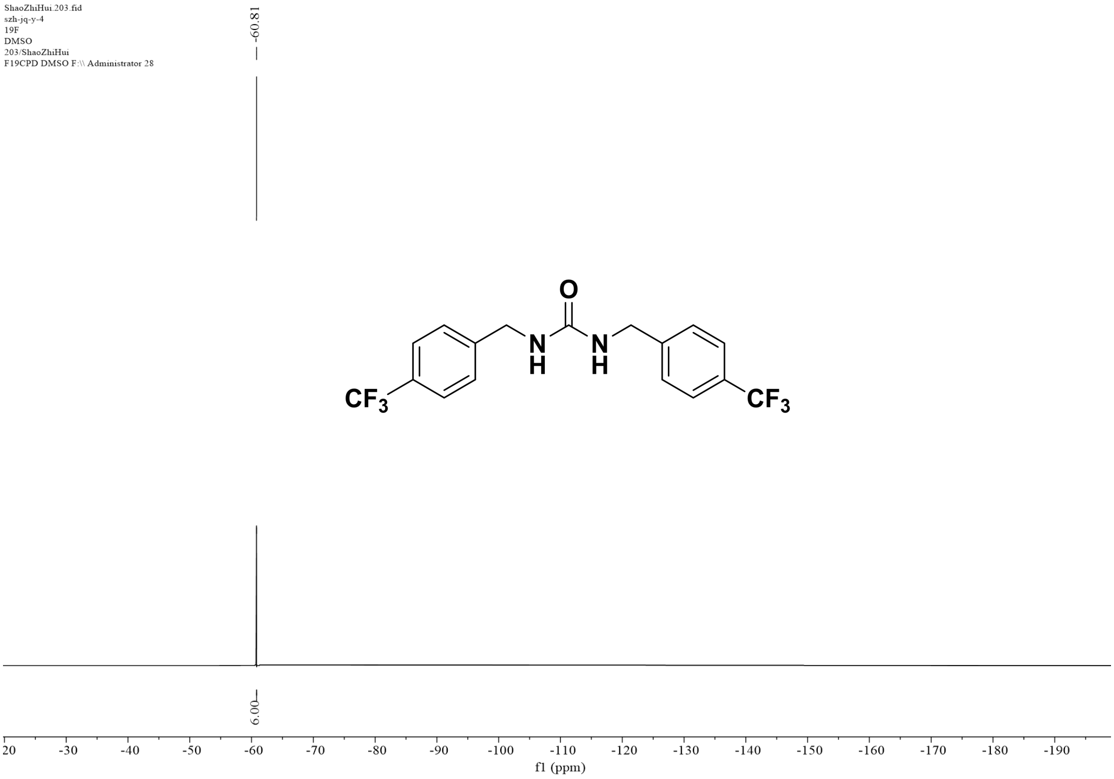

Supplementary Figure 97. ¹H NMR (400 MHz, CD_3CN) spectrum of 1m

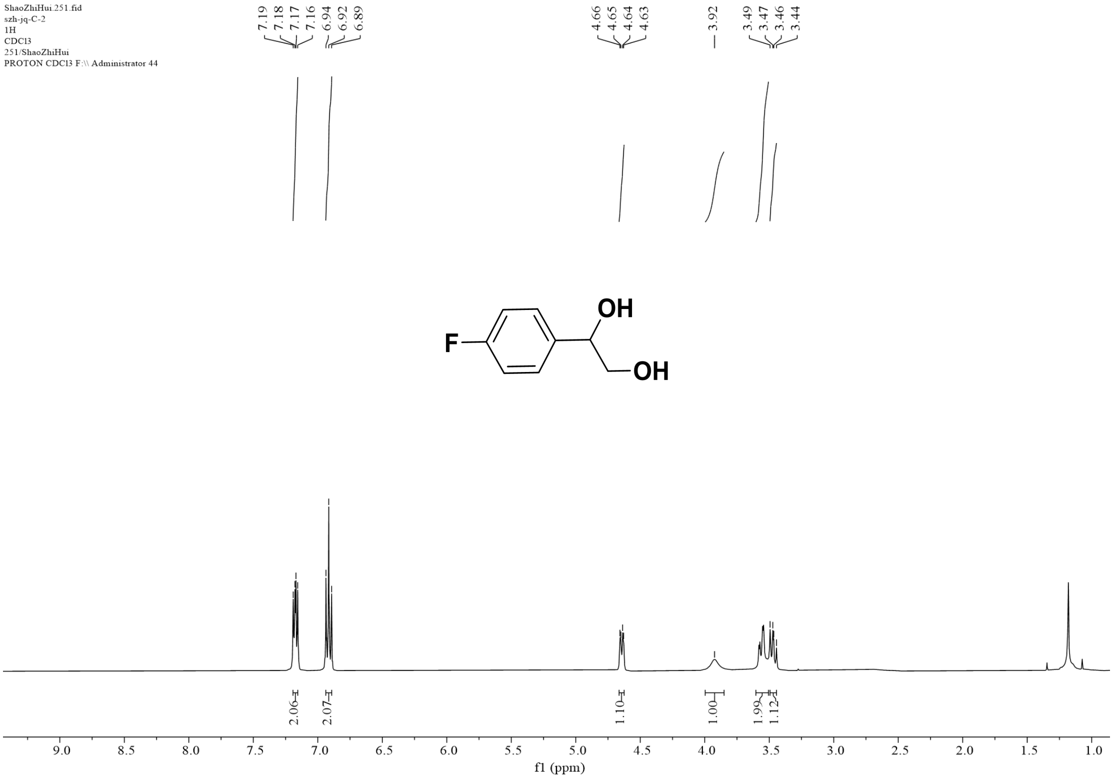

Supplementary Figure 98. ¹³C NMR (100 MHz, CD_3CN) spectrum of 1m

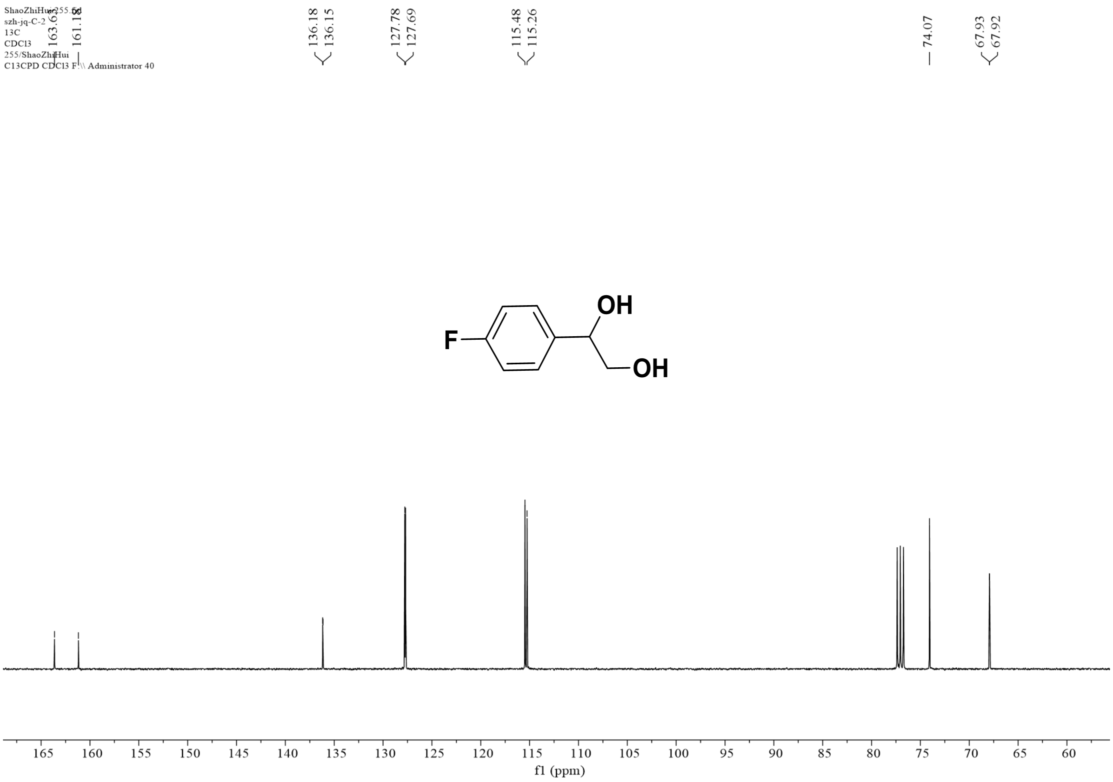

Supplementary Figure 99. ^{19}F NMR (376 MHz, CD_3CN) spectrum of 1m

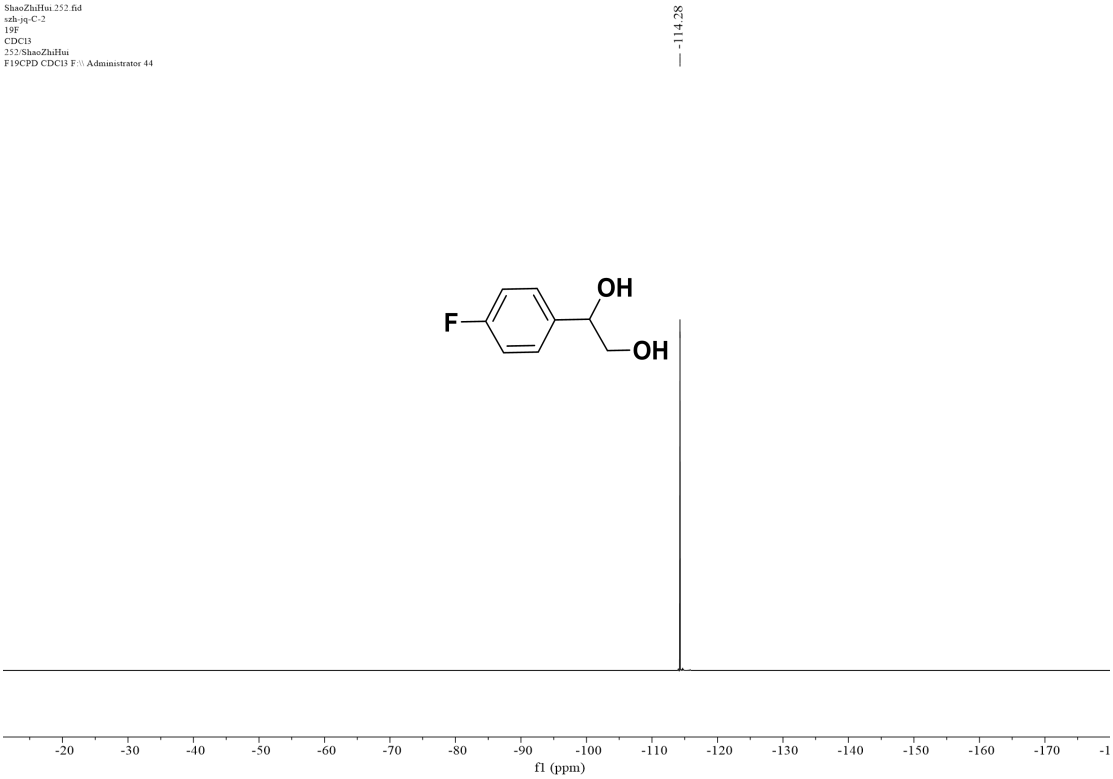

Supplementary Figure 100. ^{1}H NMR (400 MHz, CD_3CN) spectrum of 1n

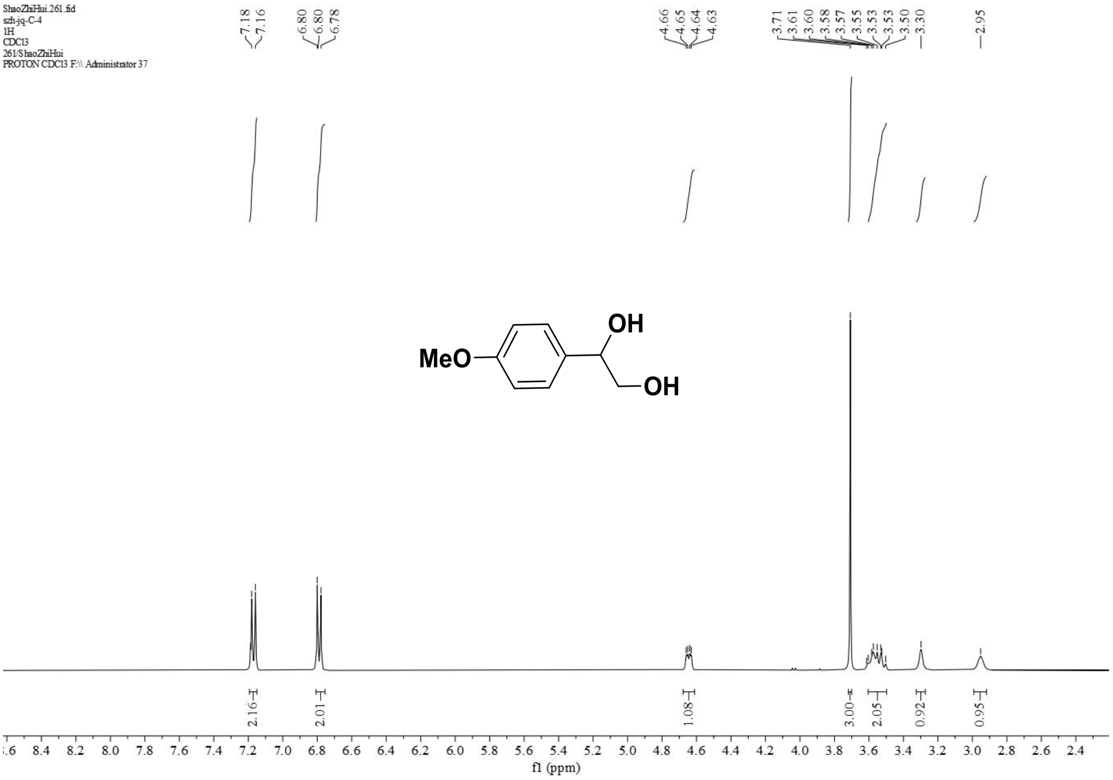

Supplementary Figure 101. ^{13}C NMR (100 MHz, CD_3CN) spectrum of 1n

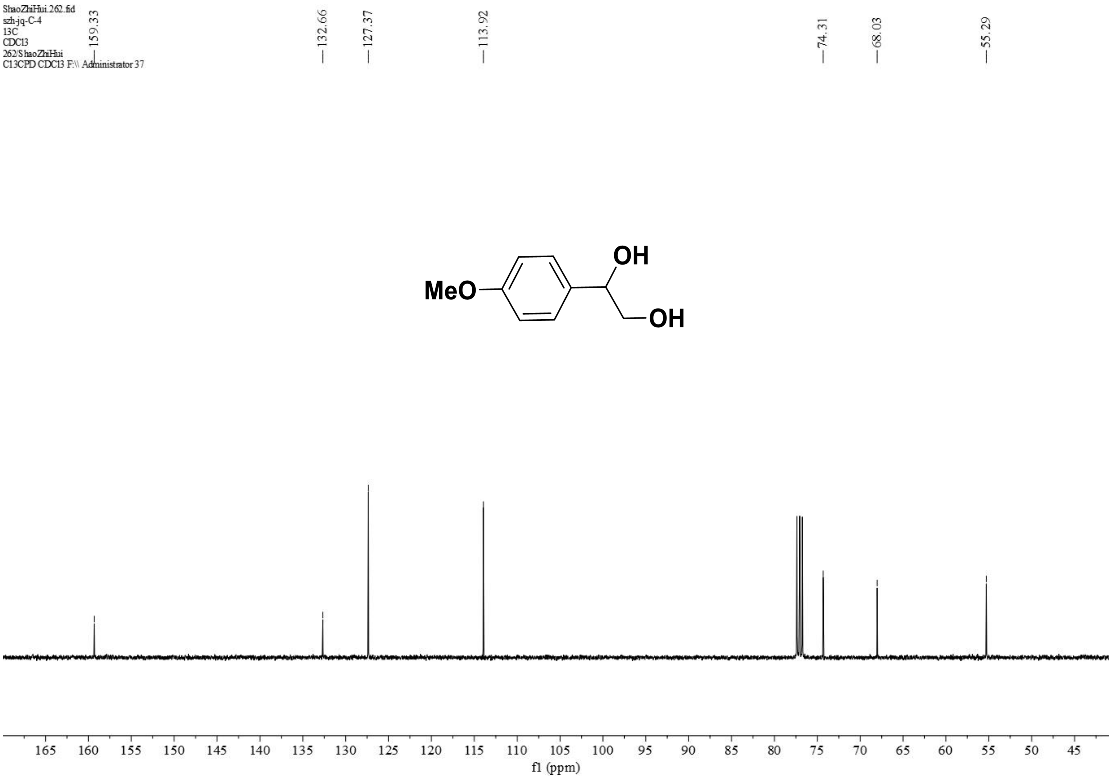

Supplementary Figure 102. ^{1}H NMR (400 MHz, CD_3CN) spectrum of 1p

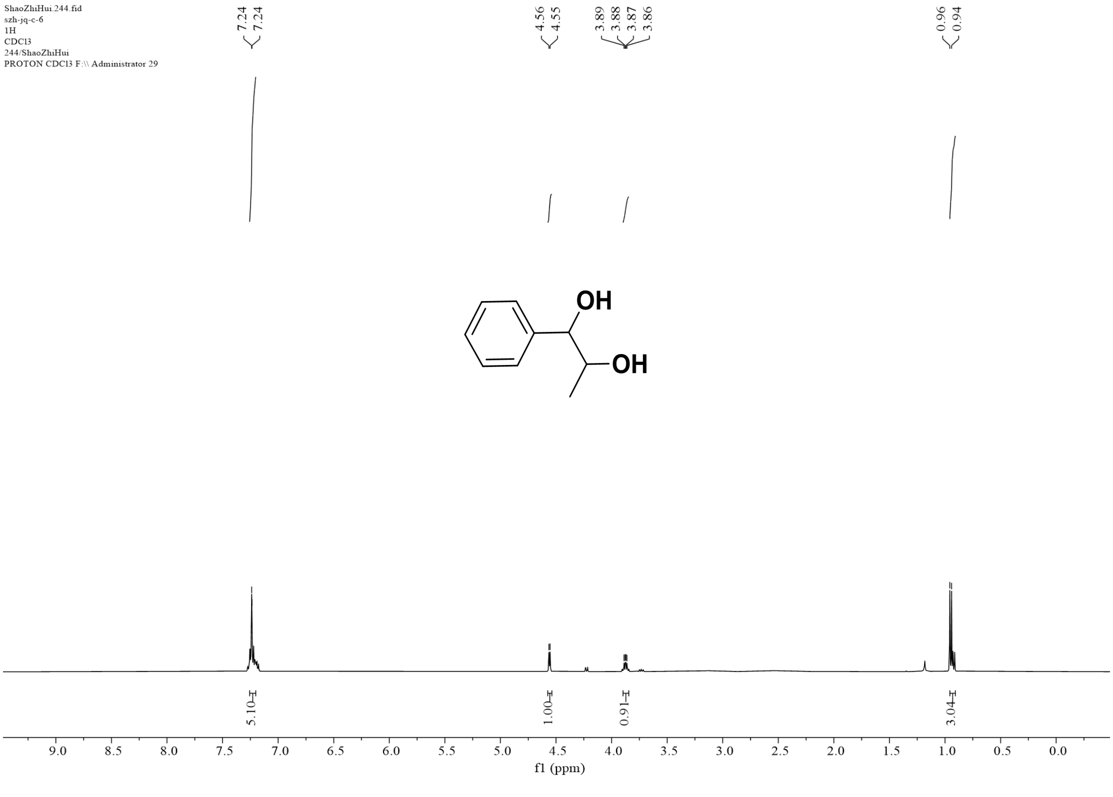

Supplementary Figure 103. ^{13}C NMR (100 MHz, CD_3CN) spectrum of 1p

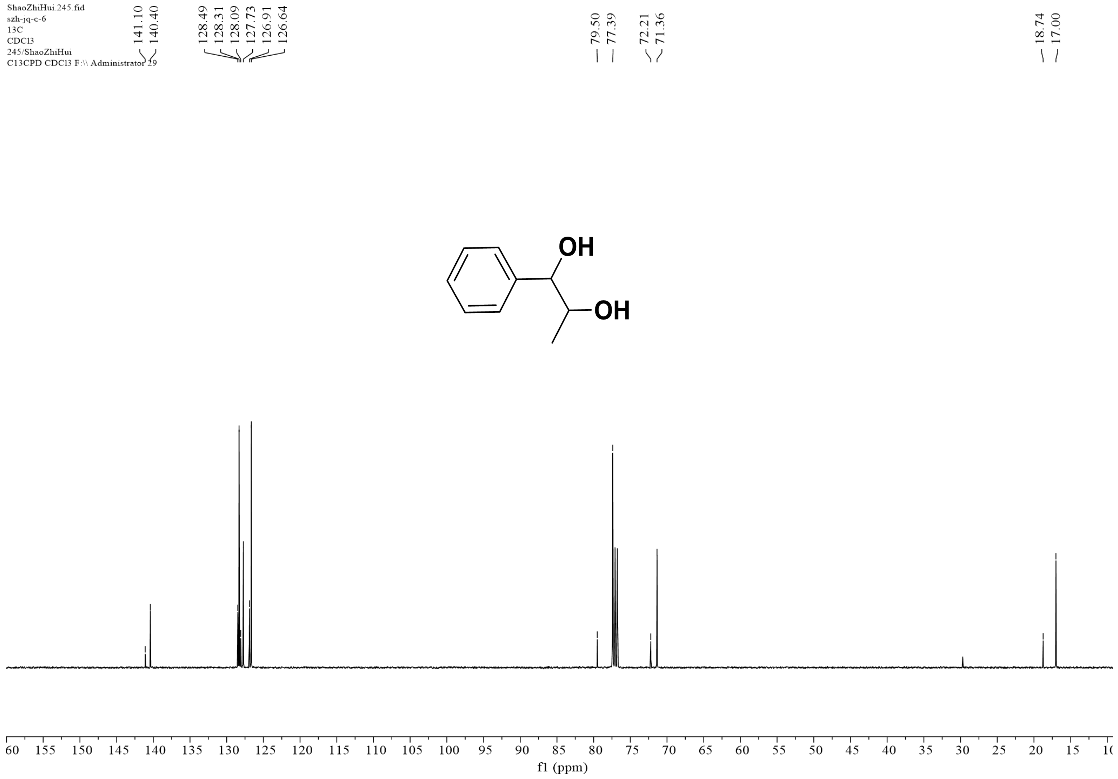

Supplementary Figure 104. ^{1}H NMR (400 MHz, CD_3CN) spectrum of 4b

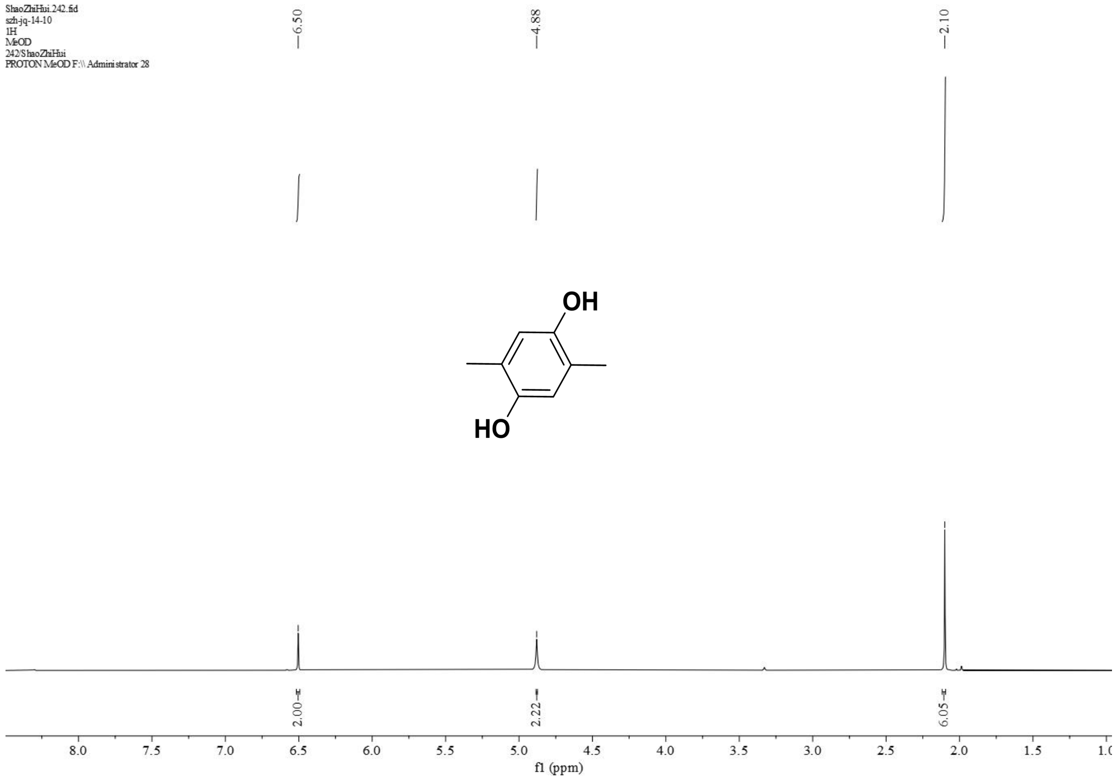

Supplementary Figure 105. ^{13}C NMR (100 MHz, CD_3CN) spectrum of 4b

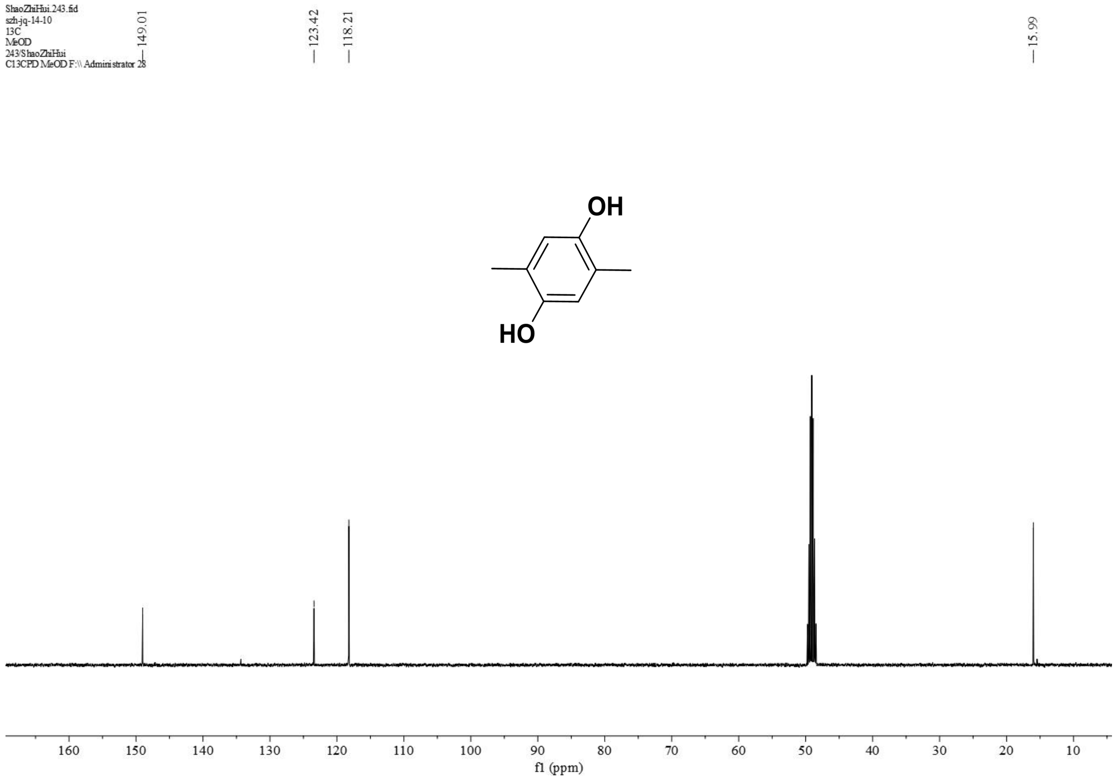

Supplementary Figure 106. ^1H NMR (400 MHz, CD_3CN) spectrum of 5b

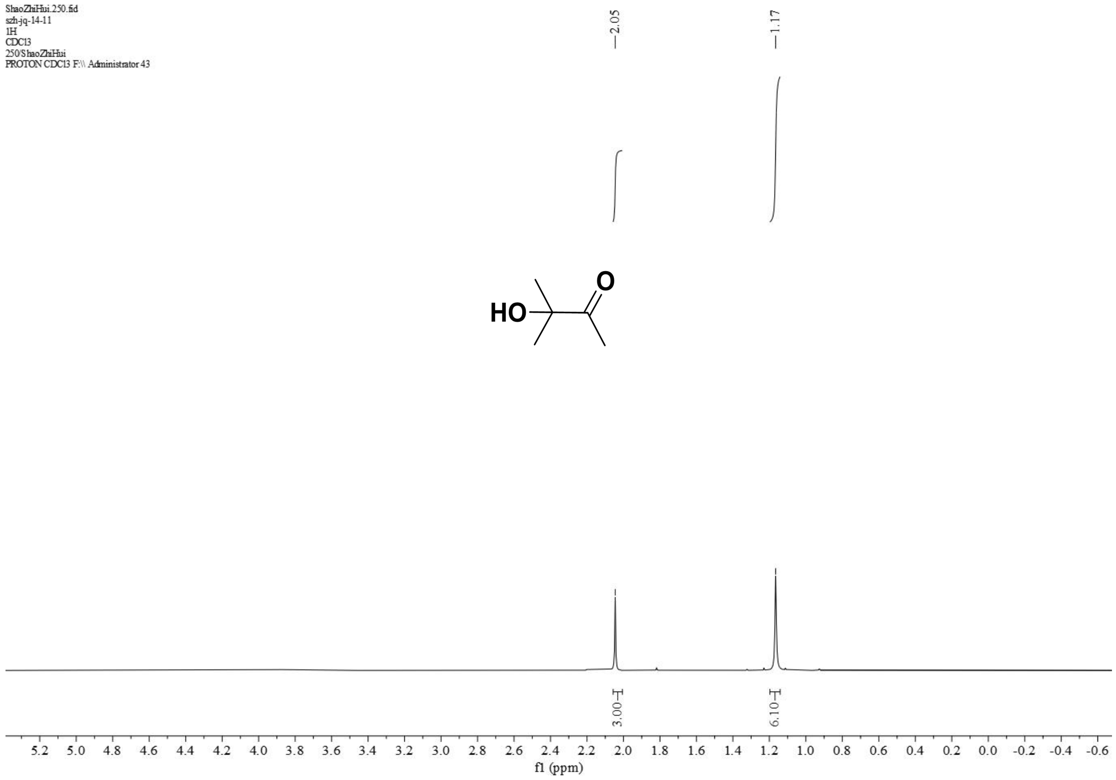

Supplementary Figure 107. ^{13}C NMR (100 MHz, CD_3CN) spectrum of 5b

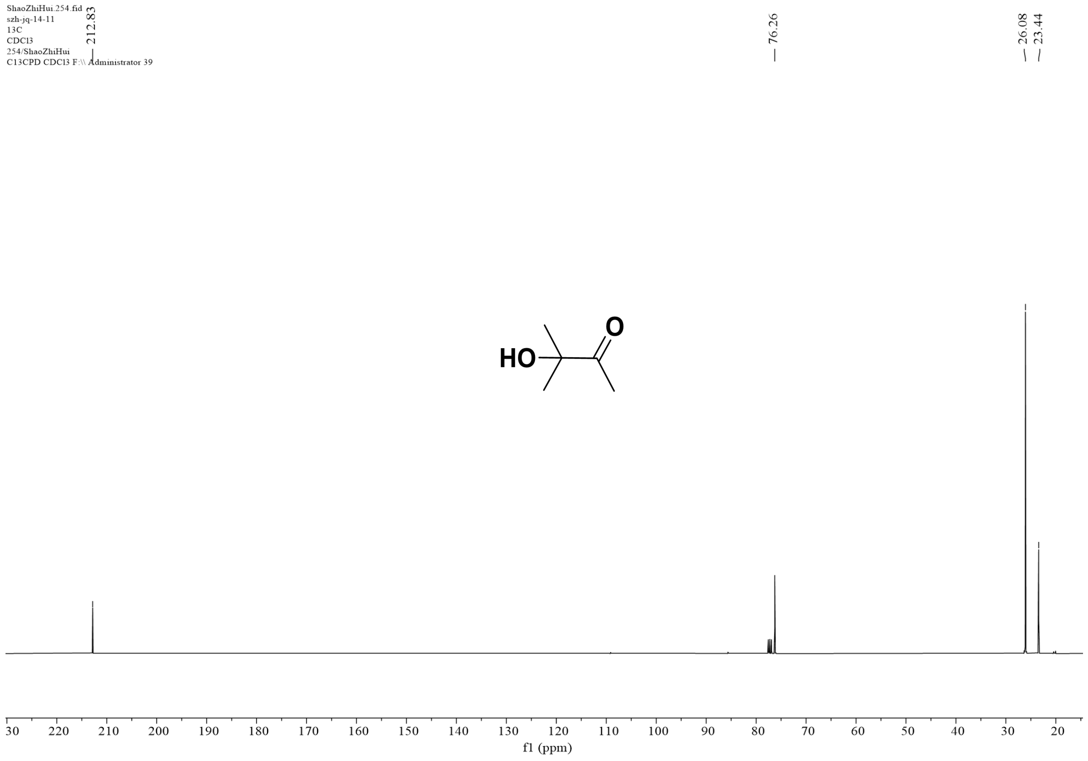

HRMS Spectra

Supplementary Figure 108. HRMS spectrum of 3b

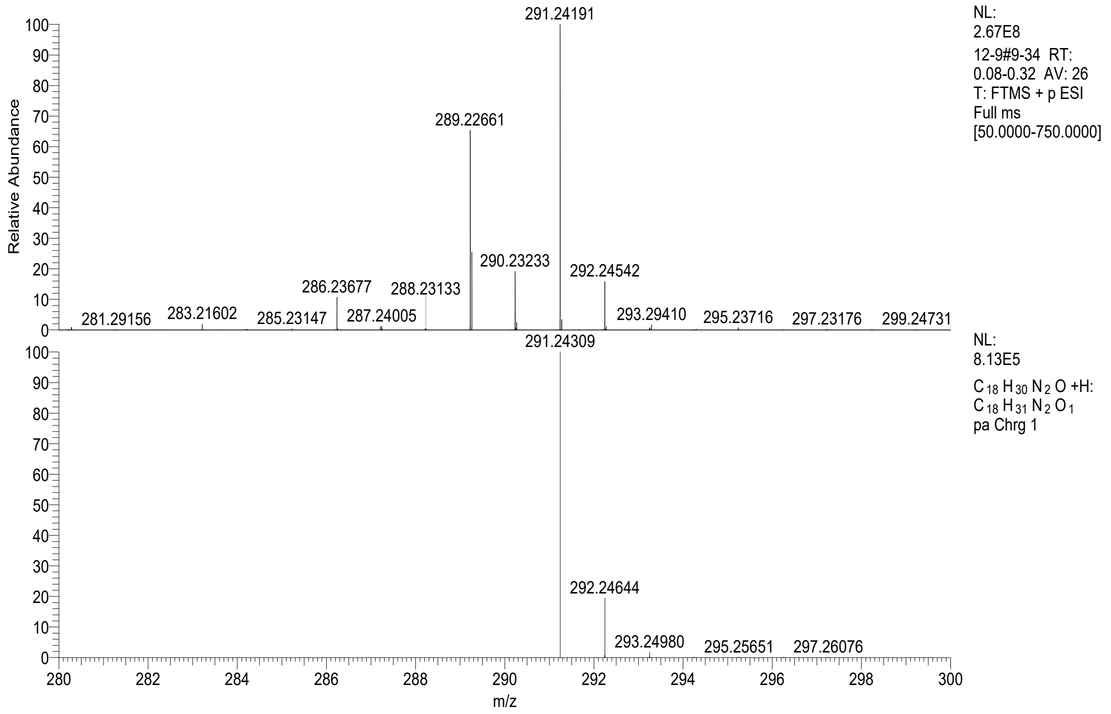

Supplementary Figure 109. HRMS spectrum of 3g

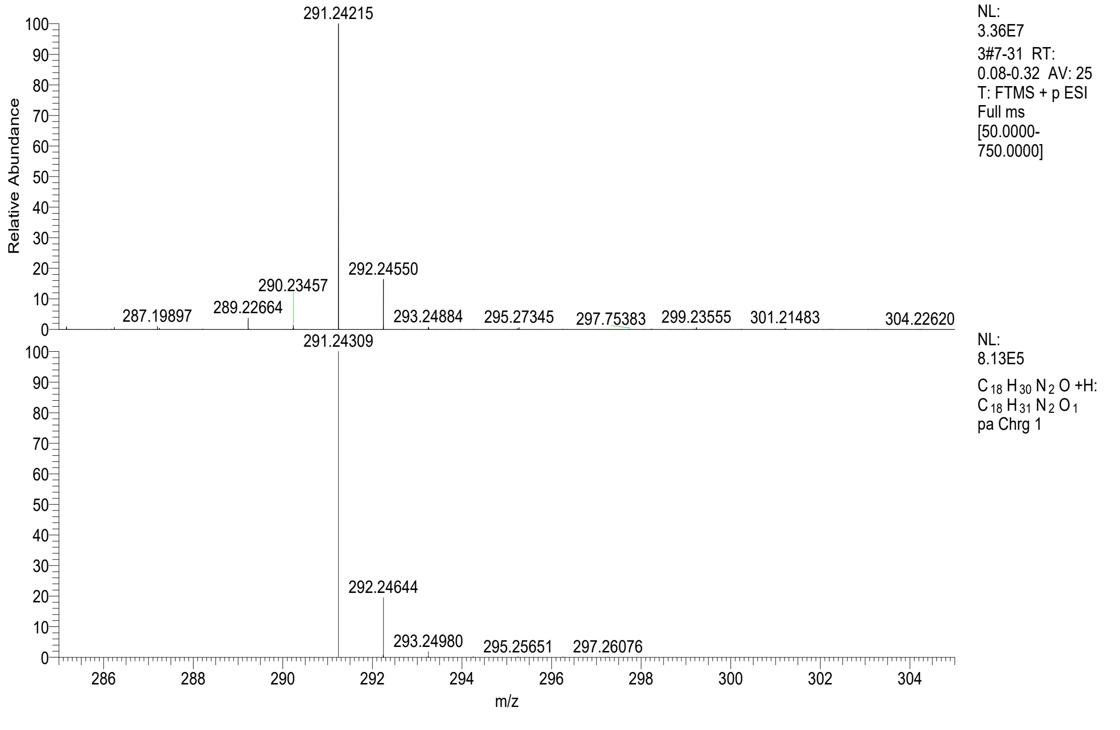

Supplementary Figure 110. HRMS spectrum of 4a

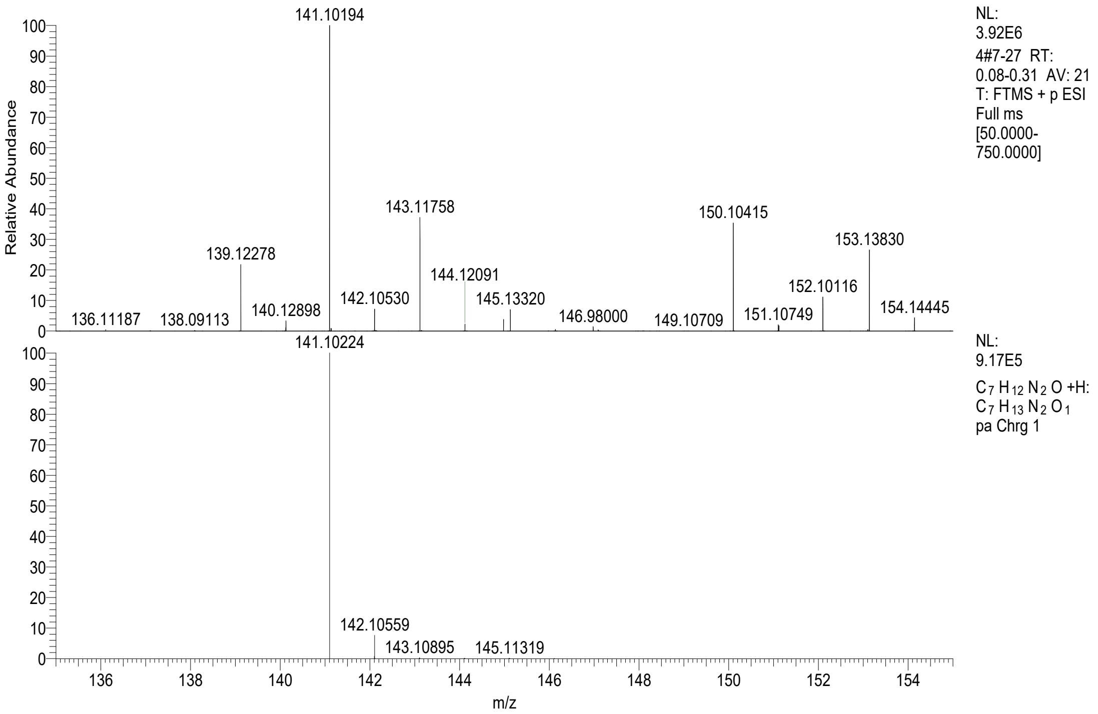

Supplementary Figure 111. HRMS spectrum of 4c

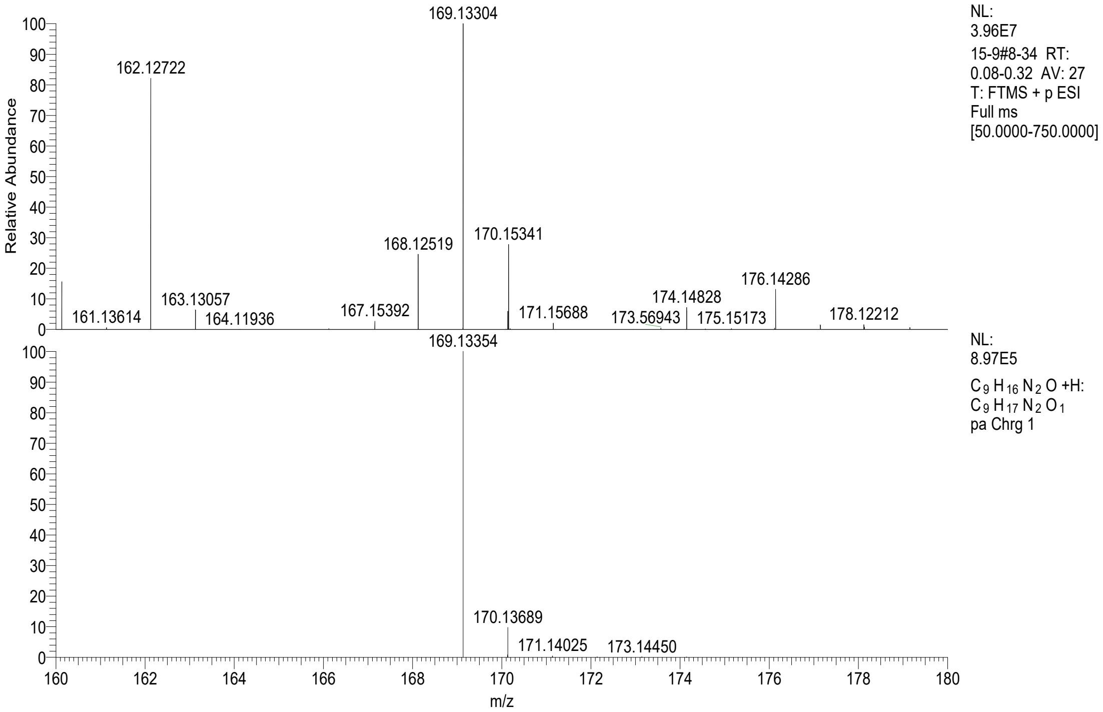

Supplementary Figure 112. HRMS spectrum of 4h

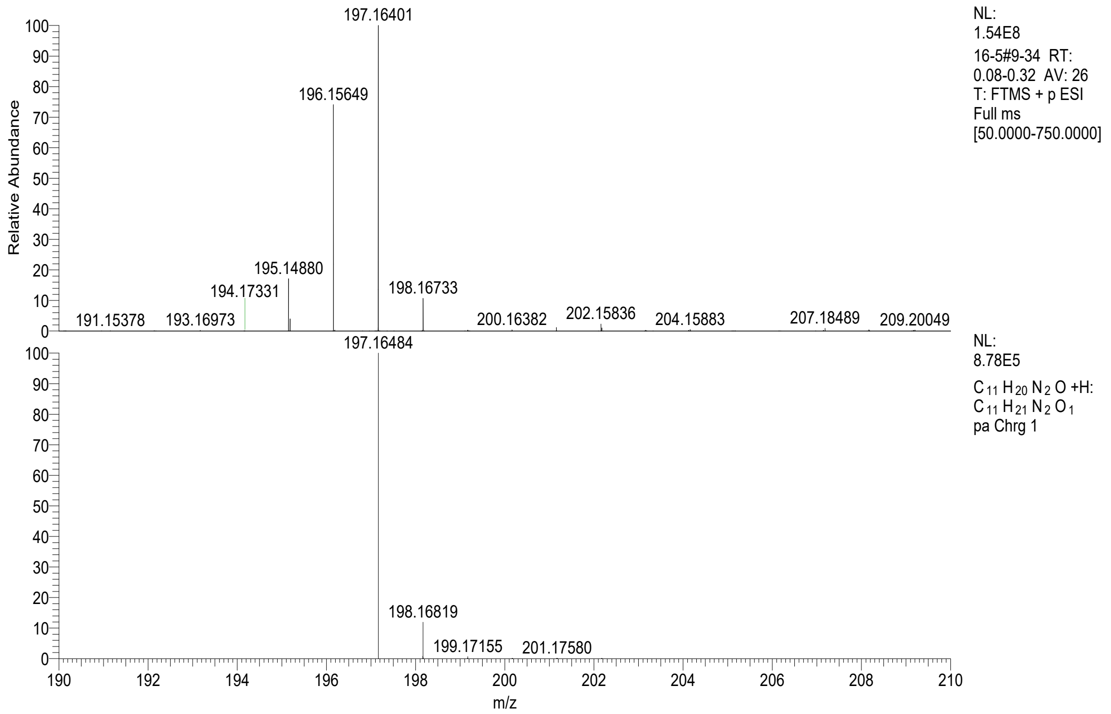

Supplementary Figure 113. HRMS spectrum of 4i

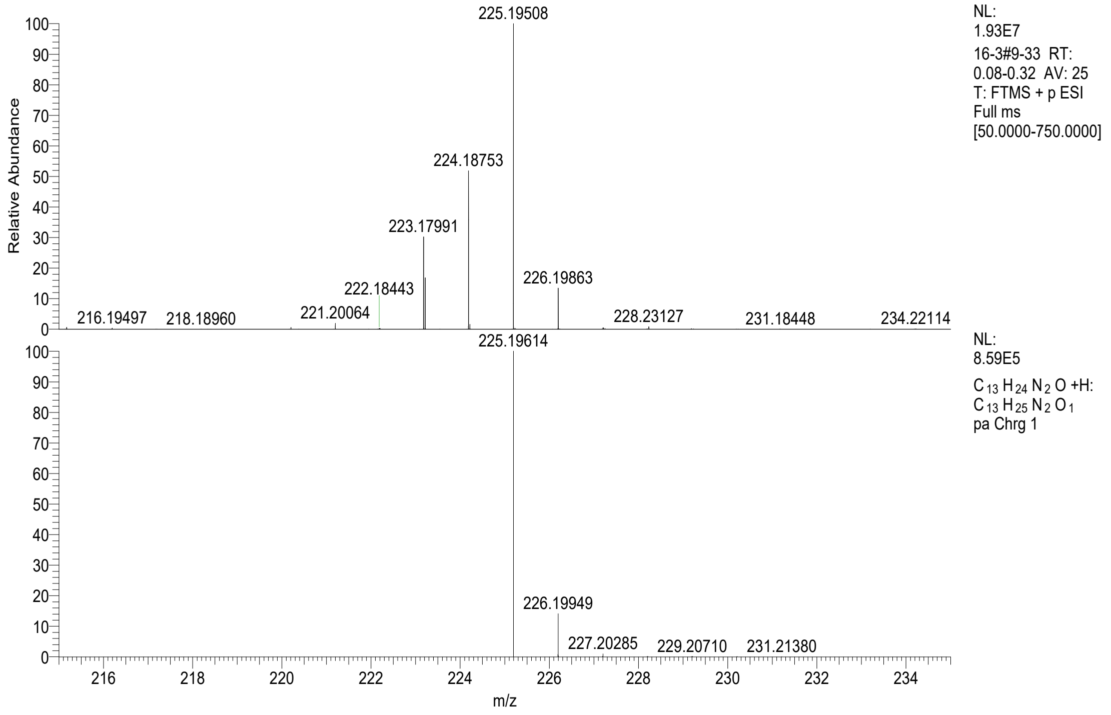

Supplementary Figure 114. HRMS spectrum of 4j

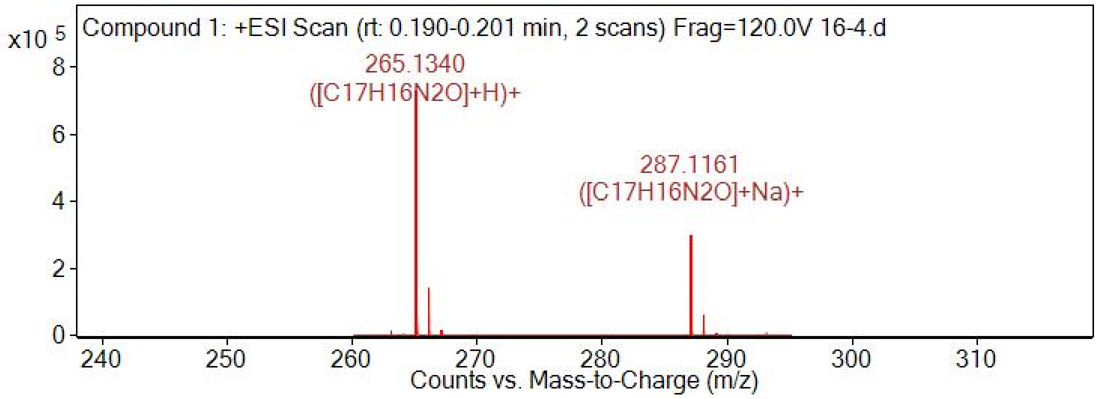

Supplementary Figure 115. HRMS spectrum of 4k

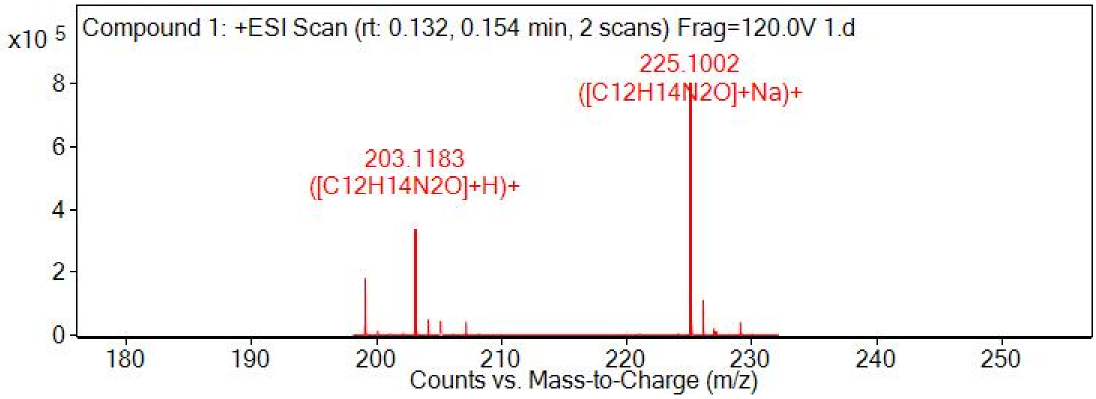

Supplementary Figure 116. HRMS spectrum of 4m

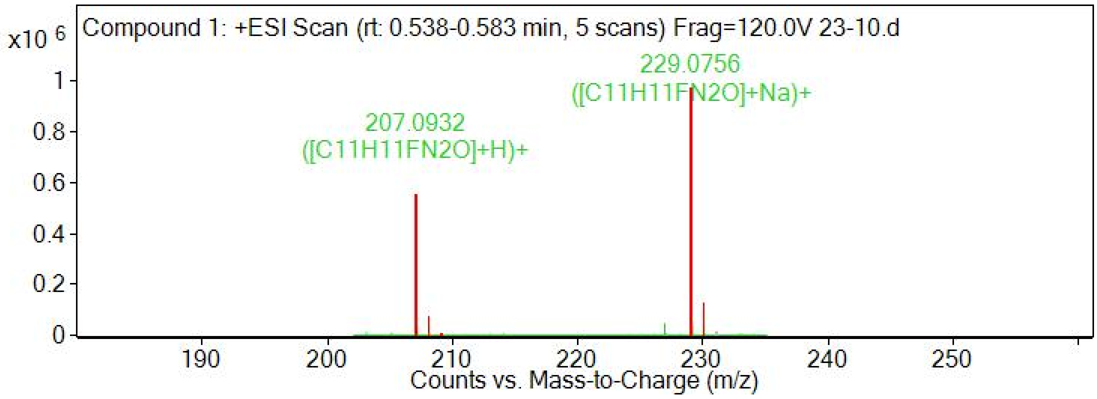

Supplementary Figure 117. HRMS spectrum of 4n

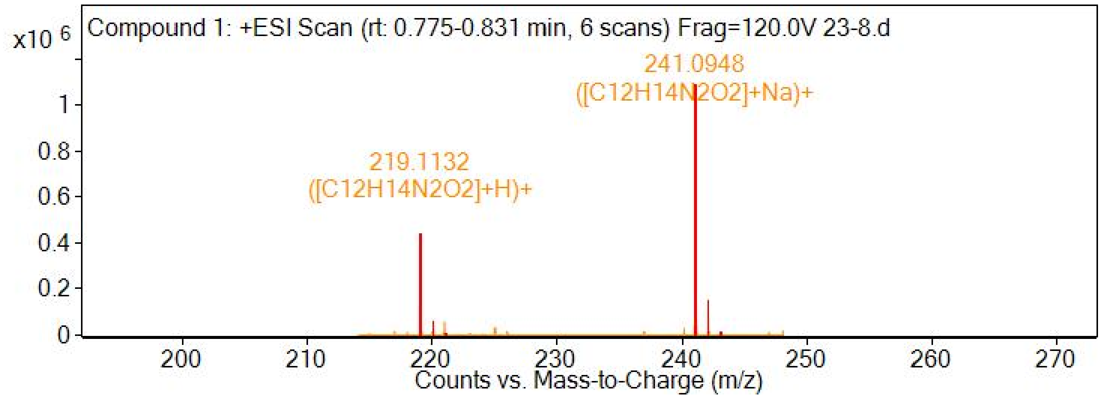

Supplementary Figure 118. HRMS spectrum of 4o

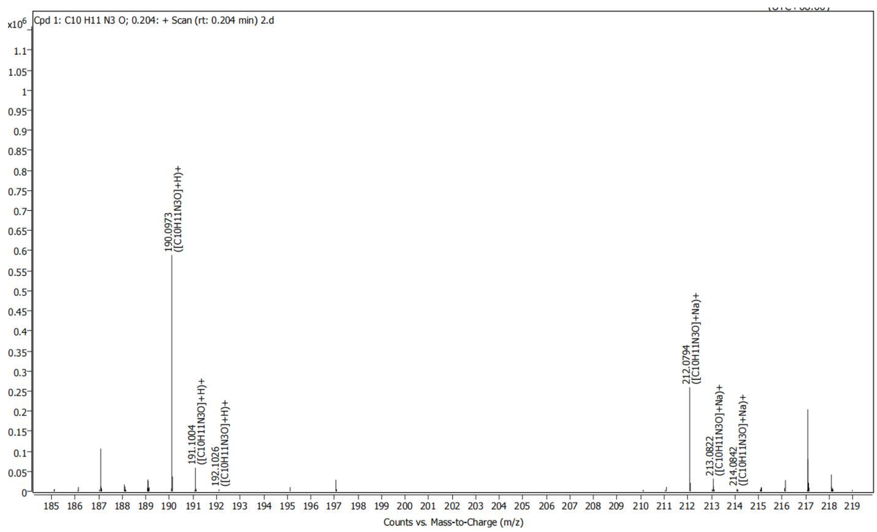

Supplementary Figure 119. HRMS spectrum of 4p

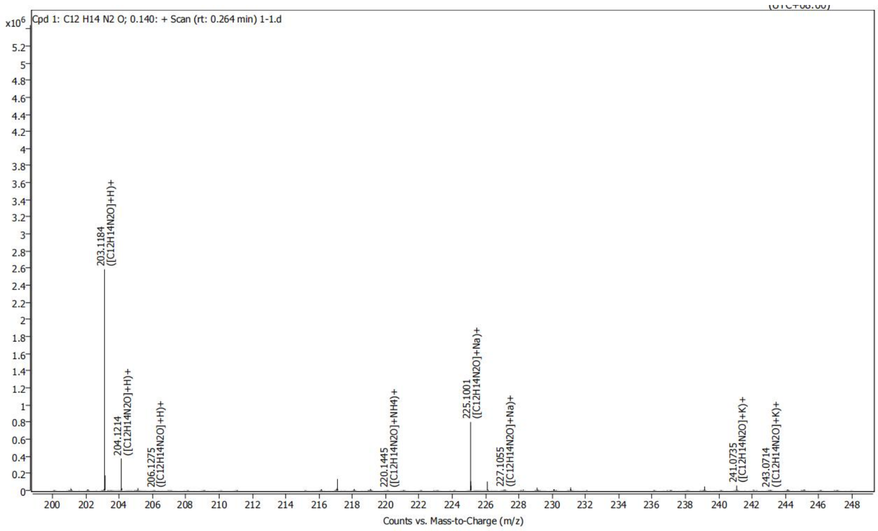

Supplementary Figure 120. HRMS spectrum of 4t

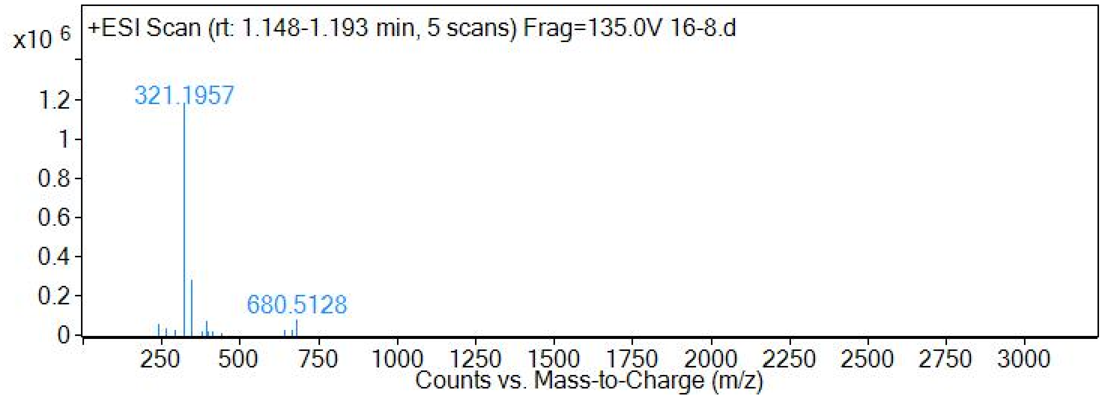

Supplementary Figure 121. HRMS spectrum of 4u

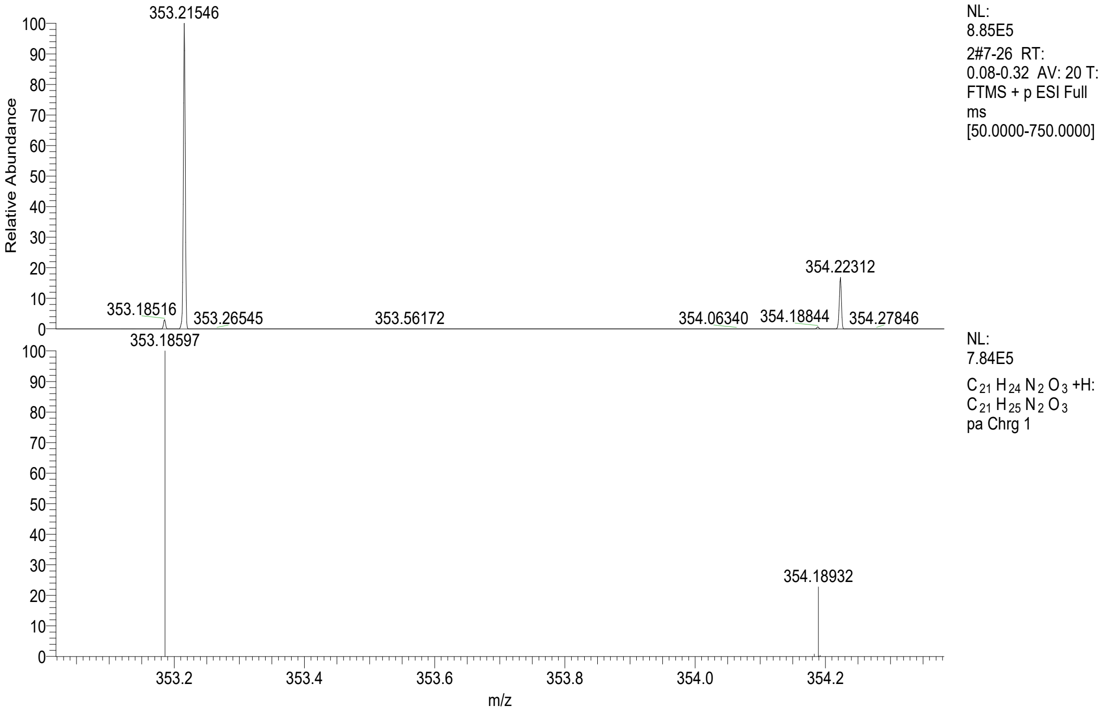

Supplementary Figure122. HRMS spectrum of 4v

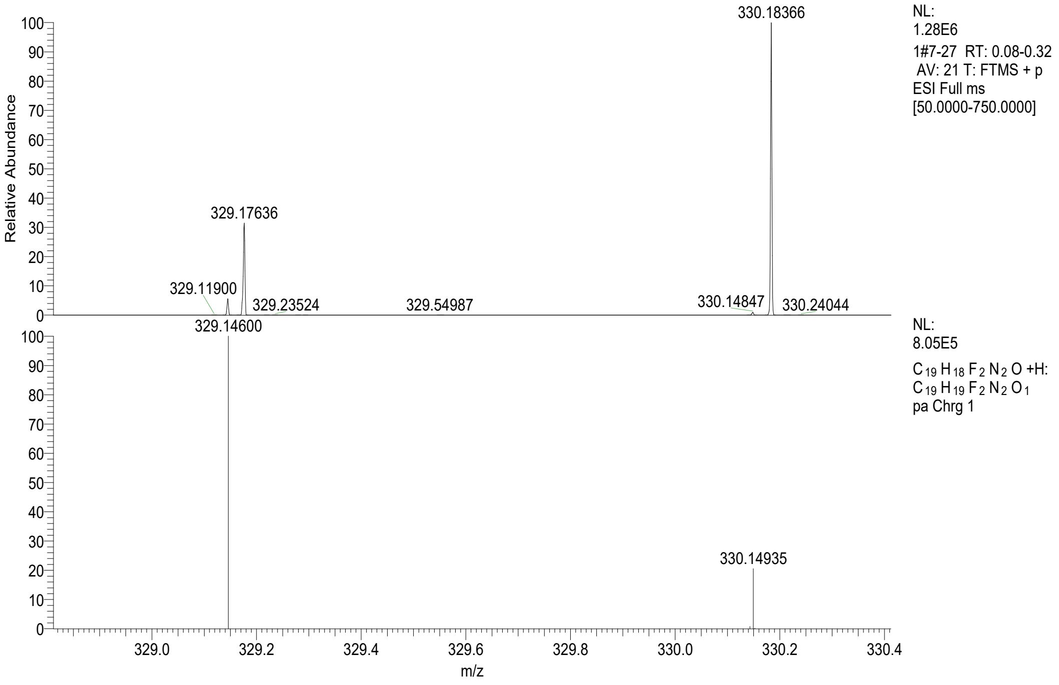

Supplementary Figure 123. HRMS spectrum of 4w

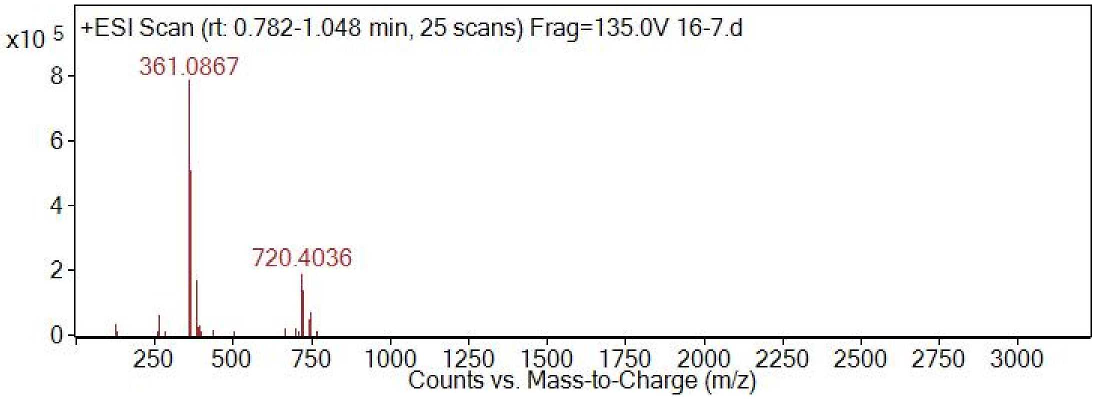

Supplementary Figure 124. HRMS spectrum of 4x

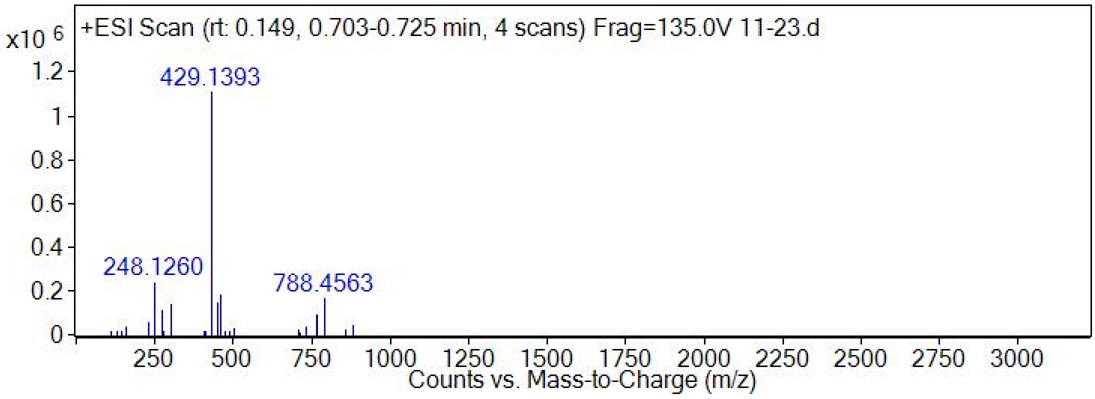

Supplementary Figure 125. HRMS spectrum of (4y+4y')

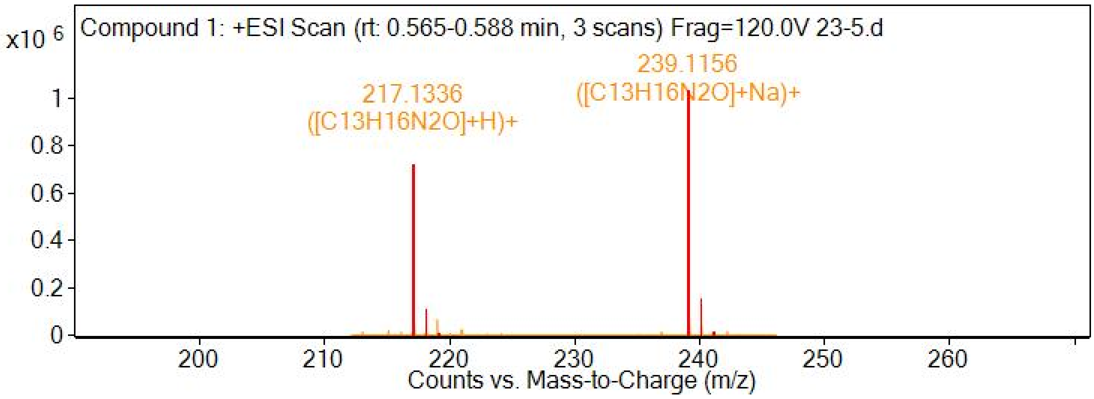

Gas collector

Gases in the gram-scale reaction after the reaction of **1a** with **2a** were collected and analyzed by GC. The evolved gas was released from the system and collected in accordance with the methods reported in our previous work[12].

GC analysis of the gas phase

GC conditions: Packed Column. Inlets: 100 °C; Detector: BID 200 °C; Carrier Gas: He; Flow: 51.4 mL/min; Oven: 35 °C, hold 2 min; 5 °C /min to 80 °C, hold 5 min.

Supplementary Figure 126. The result of GC analysis

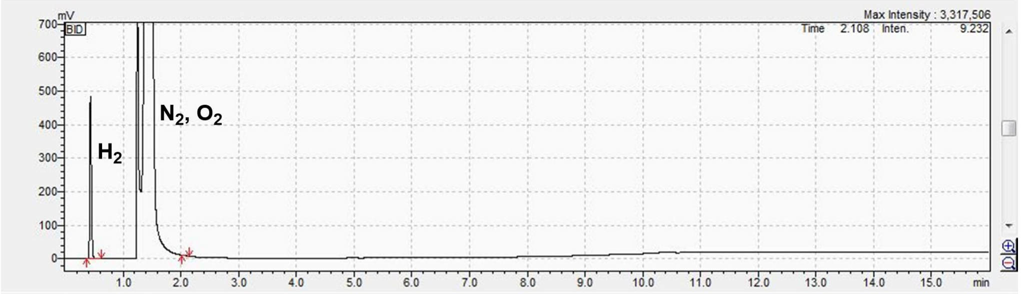

结果视图 - 峰表

峰表 | 化合物 | 组 | 校准曲线

峰号	保留时间	面积	高度	标记	浓度	浓度单位	化合物ID号	化合物名	面积%	S/N
1	0.435	951169	476386		7371.756	ppm	1	H2	99.639	3.8
2	2.033	3449	661		6.203	ppm	2	CO	0.361	0.0
总计		954618	477047		7377.959				100.000	

Kinetic studies

By monitoring the product concentration under the optimal reaction conditions at different time points, we found that the product concentration was directly proportional to time (**Figure 127**). Furthermore, we repeated the experiment using different initial solvent amounts. The results show that when the initial solvent volume was 1 mL, the relationship between the product concentration and time remained linear (**Figure 128**).

Supplementary Figure 127. Kinetic studies under the optimal reaction condition

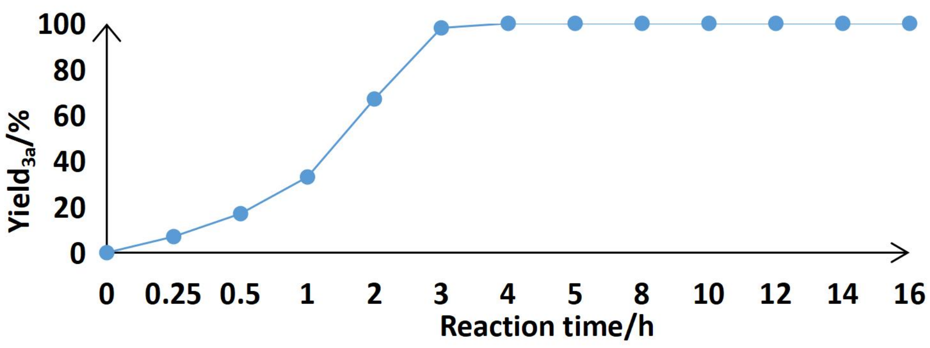

Supplementary Figure 128. Kinetic studies with 1 mL initial solvent volume

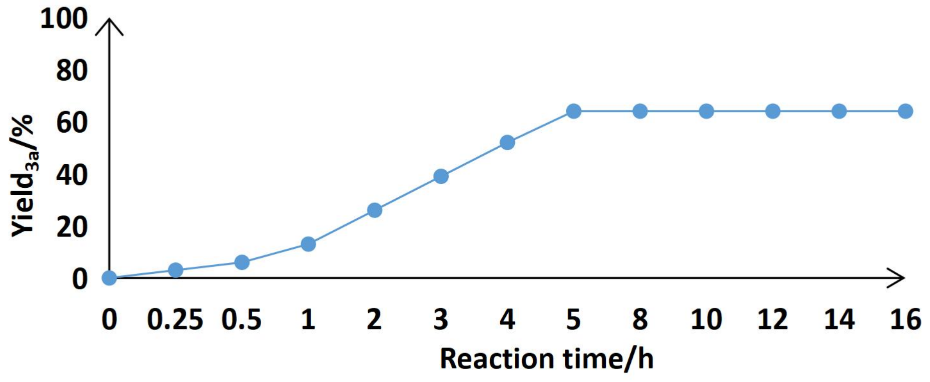

Supplementary References

[1] S. Fu, Z. Shao, Y. Wang and Q. Liu, *J. Am. Chem. Soc.* **2017**, *139*, 11941-11948.

[2] J. C. Arango-Daza, C. Lluna-Galán, L. Izquierdo-Aranda, J. R. Cabrero-Antonino and R. Adam, *ACS Catal.* **2022**, *12*, 6906–6922.

[3] J. Ariai, J. Becker and U. Gellrich, *Eur. J. Org. Chem.* **2024**, *27*, e202301252.

[4] J. Akester, J. Cui and G. Fraenkel, *J. Org. Chem.* **1997**, *62*, 431-434.

[5] T. Kondo, S. Kotachi and Y. J. Watanabe, Chem. Soc., *Chem. Commun.* **1992**, 1318-1319.

[6] N. Burford, A. D. Phillips, H. A. Spinney, K. N. Robertson, T. S. Cameron and R. McDonald, *Inorg. Chem.* **2003**, *42*, 4949–4954.

[7] B. M. Santoyo, C. González-Romero, O. Merino, R. Martínez-Palou, A. Fuentes-Benites, H. A. Jiménez-Vázquez, F. Delgado and J. Tamariz, *Eur. J. Org. Chem.* **2009**, 2505-2518.

[8] M. Wang, J. Han, X. Si, Y. Hu, J. Zhu and X. Sun, *Tetrahedron Lett.* **2018**, *59*, 1614-1618.

[9] J. Wang, H. Li, H. Wang and Y. Wu, *Org. Lett.* **2017**, *19*, 3811 - 3814.

[10] Y. Zhao, Z. Yang, B. Yu, H. Zhang, H. Xu, L. Hao, B. Han, Z. Liu, *Chem. Sci.* **2015**, *6*, 2297-2301.

[11] F. Florian, L. L. Rodrigues, S. L. Walden, B. K. Christopher, *J. Org. Chem.* **2022**, *87*, 9296-9300.

[12] Z. Shao, Y. Li, C. Liu, W. Ai, S. Luo and Q. Liu, *Nat. Commun.* **2020**, *11*, 591.
